# Dipeptidyl peptidase 4 inhibitors reduce the risk of adverse outcomes after acute kidney injury in diabetic patients

**DOI:** 10.1093/ckj/sfae385

**Published:** 2024-12-03

**Authors:** Hung-Wei Liao, Chung-Yi Cheng, Hsing-Yu Chen, Jui-Yi Chen, Heng-Chih Pan, Tao-Min Huang, Vin-Cent Wu

**Affiliations:** Division of Nephrology, Department of Internal Medicine, Wan Fang Hospital, Taipei Medical University, Taipei, Taiwan; Division of Nephrology, Department of Internal Medicine, School of Medicine, College of Medicine, Taipei Medical University, Taipei, Taiwan; Division of Nephrology, Department of Internal Medicine, Wan Fang Hospital, Taipei Medical University, Taipei, Taiwan; Division of Nephrology, Department of Internal Medicine, School of Medicine, College of Medicine, Taipei Medical University, Taipei, Taiwan; Taipei Medical University-Research Center of Urology and Kidney (RCUK), School of Medicine, College of Medicine, Taipei Medical University, Taipei, Taiwan; Graduate Institute of Clinical Medical Sciences, College of Medicine, Chang Gung University, Taoyuan, Taiwan; Division of Chinese Internal Medicine, Center for Traditional Chinese Medicine, Chang Gung Memorial Hospital, Taoyuan, Taiwan; School of Traditional Chinese Medicine, College of Medicine, Chang Gung University, Taoyuan, Taiwan; Division of Nephrology, Department of Internal Medicine, Chi Mei Medical Center, Tainan, Taiwan; Department of Health and Nutrition, Chia Nan University of Pharmacy and Science, Tainan, Taiwan; Graduate Institute of Clinical Medicine, College of Medicine, National Taiwan University, Taipei, Taiwan; Chang Gung University College of Medicine, Taoyuan, Taiwan; Division of Nephrology, Department of Internal Medicine, Keelung Chang Gung Memorial Hospital, Keelung, Taiwan; Community Medicine Research Center, Keelung Chang Gung Memorial Hospital, Keelung, Taiwan; Division of Nephrology, Department of Internal Medicine, National Taiwan University Hospital, Taipei, Taiwan; Primary Aldosteronism Center of Department of Internal Medicine, National Taiwan University Hospital, Taipei, Taiwan; NSARF (National Taiwan University Hospital Study Group of ARF), TAIPAI (Taiwan Primary Aldosteronism Investigators), and CAKS (Taiwan Consortium for Acute Kidney Injury and Renal Diseases), Taipei, Taiwan

**Keywords:** acute kidney disease, acute kidney injury, dipeptidyl peptidase 4 inhibitors, major adverse kidney events, mortality

## Abstract

**Background:**

Dipeptidyl peptidase 4 inhibitors (DPP4is) are considered safe for use in patients with diabetes mellitus and kidney dysfunction. We explored whether usage of DPP4is in patients who recovered from dialysis-requiring acute kidney injury (AKI) could reduce the risk of future cardiac and kidney events.

**Methods:**

We used the TriNetX platform to investigate whether the use of DPP4is in diabetes mellitus patients within 90 days of discharge from acute kidney disease could reduce the risk of all-cause mortality, major adverse kidney events (MAKEs), major adverse cardiovascular events (MACEs), and re-dialysis. The patients were followed for 5 years or until the occurrence of significant outcomes, with cohort data collected from 1 January 2016 to 30 September 2022.

**Results:**

The cohort utilizing DPP4is comprised 7348 patients with acute kidney disease, while the control group encompassed 229 417 individuals. After applying propensity score matching, 7343 patients (age 66.2 ± 13.4 years; male, 49.9%) who used DPP4is showed a significant reduction in the risk of all-cause mortality [adjusted hazard ratio (aHR) 0.89; E-value 1.50 , MAKEs (aHR 0.86; E-value 1.59), MACEs (aHR 0.91; E-value 1.44), and re-dialysis (aHR 0.73; E-value 2.10) after a median follow-up of 2.4 years.

**Conclusions:**

We demonstrated that in diabetes mellitus patients concurrently experiencing acute kidney disease, DPP4i usage could decrease the risk of mortality, MAKEs, MACEs, and re-dialysis. These findings emphasize the pivotal role of tailored treatment strategies involving DPP4i for acute kidney disease patients.

## Graphical Abstract

**Graphical Abstract:**
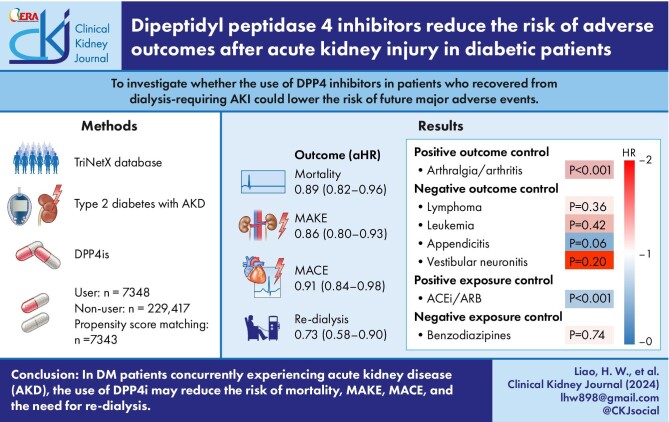

KEY LEARNING POINTS **What was known:**

- Acute kidney injury may progress to CKD. Dipeptidyl peptidase-4 inhibitors (DPP4is) are considered safe for diabetes mellitus patients with kidney dysfunction. This study investigated whether the use of DPP4is in patients who have recovered from dialysis following acute kidney injury can reduce the risk of future major adverse events.

**This study adds:**

- This study utilized the TriNetX database, which provides comprehensive real-world practice data at a large scale, to support the incorporation of DPP4is into the management regimen for type 2 diabetes patients following an episode of acute kidney injury.

**Potential impact:**

- Healthcare providers are compelled to deliberate upon the inclusion of DPP4is within the therapeutic paradigm for patients concurrently having diabetes concomitant with acute kidney disease.

## INTRODUCTION

Acute kidney injury (AKI) refers to a sudden decline in kidney function. The incidence of AKI is notably higher among hospitalized patients, with reported rates reaching ∼22% [1]. Patients who experience AKI during their hospitalization are also at an elevated risk for various adverse outcomes in the future [2, 3]. The aforementioned studies revealed that AKI-requiring RRT was associated with a 28-fold increased risk of developing subsequent stage 4 or higher CKD [4]. Acute kidney disease (AKD) refers to the ongoing progression of the disease after AKI, wherein the renal pathophysiologic processes continue. It serves as an intermediate stage between AKI and the development of CKD, within 90 days following an AKI episode [5]. AKD status was found to be associated with a higher incidence of all-cause mortality, progressive CKD, end-stage kidney disease [6] and cardiovascular morbidity [7].

The incidence of AKI in diabetic mellitus (DM) patients has been reported to be approximately five times higher than in those without DM [8]. Due to the high global prevalence of DM, there is a pressing concern within the healthcare system to prevent the progression of AKI in DM patients to AKD or other major adverse outcomes, such as death. Dipeptidyl peptidase 4 inhibitors (DPP4is) are considered safe for use in patients with kidney dysfunction, unlike some other oral hypoglycemic agents [9].

TriNetX is a global collaborative health research network platform that provides access to electronic medical records and data from diverse healthcare organizations in real-world practice. Real-world data hold great potential for designing and conducting confirmatory trials and answering questions that may not be addressed otherwise [10]. For this study, we utilized the international TriNetX platform to investigate whether the use of DPP4is could prevent AKD and long-term adverse outcomes in diabetic patients with AKD.

## MATERIALS AND METHODS

### Data collection

The TriNetX platform currently comprises 75 healthcare organizations providing data [11–13]. For our study, we utilized data obtained from the TriNetX Network. The available data included comprehensive information about patient demographics, diagnoses (based on the International Classification of Diseases, Tenth Revision, Clinical Modification, ICD-10-CM codes), procedures (coded in The International Classification of Diseases, Tenth Revision, Procedure Coding System, ICD-10-PCS, or Current Procedural Terminology, CPT), medication (coded in the Veterans Affairs National Formulary), laboratory tests (coded in Logical Observation Identifiers Names and Codes, LOINC), and healthcare utilization. The cohort examined in the current investigation encompasses data collected from 1 January 2016 to 30 September 2022 within the TriNetX Network

### Ethics statement

For this study, the Affiliated Hospital of Chi-Mei Hospital's Board of Directors granted approval for data collection through the TriNetX Network under institutional review (no. 11202-002). The collection of data from the TriNetX platform adhered to the regulations outlined by the Health Insurance Portability and Accountability Act (HIPAA) and the General Data Protection Regulation (GDPR) [14]. As the TriNetX platform only aggregates de-identified information into counts and statistical summaries, the Western Institutional Review Board granted a waiver for the right-to-know requirement for TriNetX [15]. Our study was conducted in adherence to the principles outlined in the Declaration of Helsinki.

### Cohort

The construction of the cohort utilized in this study is depicted in Supplementary Fig. S1. The age range of the participants spanned from 18 to 90 years, with a total enrollment of 90 571 386 individuals within the TriNetX platform. Our study specifically centered on patients who underwent the most severe form of dialysis requiring AKI but subsequently discontinued dialysis upon discharge. The etiologies of AKI are shown in Supplementary Table S1.

The inclusion criteria for the study were as follows: (i) patients who had ever undergone dialysis during their hospitalization before discharge, (ii) patients discharged before 30 September 2022, (iii) patients who survived and did not undergo dialysis within 3 months after discharge, and (iv) patients who received DPP4is after discharge within 3 months in the intervention group, while the control group consisted of diabetic patients without DPP4i usage after discharge. For all participants, the index date was determined as the day after their hospital discharge. Furthermore, the 3-month window helps mitigate reverse causality effects, ensuring outcomes are more accurately attributed to the DPP4 inhibitors (Supplementary Fig. S2). It also ensures data reliability, as immediate post-discharge records can be inconsistent. In this study, AKD was defined as patients who had undergone dialysis during their hospitalization for severe AKI but did not require dialysis within 3 months post-discharge. Serum markers, such as serum creatinine, may not be reliable indicators of kidney function during the recovery period of AKI [16]. Therefore, in our study, patients who experienced severe AKI requiring dialysis were considered to have AKD, regardless of their serum marker status post-discharge.

### Pre-specified outcomes

The individuals from the cohort were followed longitudinally for a duration ranging up to 5 years from the index date, allowing us to evaluate the risk of the specified outcomes of interest. For our specific analysis, we focused on predetermined outcomes, including mortality, major adverse kidney events (MAKEs), and major adverse cardiovascular events (MACEs). In this study, the definition of MAKE included total death, or the necessity for re-dialysis once again. On the other hand, the definition of MACE included total death, cardiac arrest or shock, non-fatal stroke, and non-fatal myocardial infarction. To mitigate protopathic or ascertainment bias, any events of secondary outcomes that occurred before the index date were excluded, and repeat propensity score matching was performed.

Subgroup analyses were performed to explore the variations in risk for the desired outcomes among AKD patients using DPP4is, considering baseline factors such as hypertension, heart failure, BMI, estimated eGFR, angiotensin converting enzyme inhibitor/angiotensin receptor blocker (ACEi/ARB) usage, and concurrent administration of other blood glucose control medications.

### Covariates

To account for disparities in baseline characteristics between the two groups, we included the following covariate factors in our analysis: demographic covariates (age at index, gender, race), comorbidities (ischemic heart disease and cerebrovascular disease), and medication usage, including ACEis, angiotensin II receptor blockers, and aspirin.

In addition, our analysis incorporated adjustments for various parameters, including BMI and eGFR. These adjustments were implemented to mitigate potential confounding factors and to account for disparities in baseline characteristics between the two groups.

### Pre-specified subgroup analyses

Subgroup analyses were conducted to examine variations in risk related to the desired outcomes among the DPP4i users. These pre-specified analyses considered factors such as age, hypertension, heart failure (HF), eGFR, proteinuria, enrollment period, history of DPP4i use before index day, concurrent usage of other medications for glycemic control, and renin–angiotensin–aldosterone system (RAAS) blockers.

### Statistical analyses

Propensity score matching was employed to minimize the influence of confounding factors and generate groups with balanced baseline characteristics. We utilized the TriNetX built-in statistics to match the two groups at a 1:1 ratio. Matching variables included age at index, gender, race, comorbidities, laboratory parameters, and medication usage. The balance of baseline characteristics in the propensity score-matched populations was assessed using the standardized difference, with a value <0.1 considered indicative of a small difference [17]. Adjusted hazard ratios (aHRs) were calculated to assess the impact of DPP4i usage on the desired outcomes, compared with the control group by a Cox proportional hazard model. Any instances of missing data pertaining to the outcomes or lost to follow-up were addressed by excluding the respective cases from the analysis. This exclusion criterion was applied to avoid any bias or inaccuracies that might arise due to incomplete data. In order to lessen the effect of reverse causality, the observation period was initiated 90 days following the index date and continued for a maximum duration of 5 years. E-values were calculated for pre-specified outcomes to address the unmeasured confounders [18]. Moreover, to bolster the credibility of our findings, we executed an external validation process by leveraging data sourced from the Chang Gung Research Database [19]. This validation was augmented by sensitivity assessments [19], encompassing evaluations of diverse cases over different registration periods and Cox proportional analysis with distinct covariates. Additionally, analyses scrutinizing specificity, positive outcome controls, and designated negative outcome controls were undertaken, with detailed information provided in the Supplementary appendix. Multivariable regression analysis was conducted by using TriNetX's built-in logistic regression analysis to evaluate the influence of confounding factors on the outcomes. Analytical tools employed included R software (version 3.2.2, Free Software Foundation, Inc., Boston, MA), SAS (version 9.2, SAS Inc., Cary, NC), and Stata/MP (version 16, StataCorp, College Station, TX). Statistical analyses used the Kaplan–Meier method, and statistical significance was determined as *P* < .05, considering a 95% confidence interval (95% CI).

## RESULTS

### Baseline characteristics of the participants

In the DPP4i-using group, there were 7348 patients who had experienced AKI, while in the control group there were 229 417 patients who had not used DPP4is (Supplementary Fig. S1). The prevalence of DPP4i usage was 3.1% (Table 1). The average age of the individuals taking DPP4is was 66.2 ± 13.4 years, which was higher than the average age of their counterparts (62.1 ± 15.8 years). Within the DDP4i group, ∼49.9% were males, whereas the control group consisted of 52.7% males. Among the DPP4i users, there was a higher proportion of individuals with ischemic heart disease (41.9%) compared with those who did not use DPP4is (34.1%). Moreover, the DPP4i users had a higher proportion of individuals using ACEis (44.1%) or ARBs (28.6%), in comparison with the DPP4i non-users, where the percentages were 31.1 for ACEis and 16.6 for ARBs The mean eGFRs for the DDP4i and control groups were 70.5 ± 35.5 and 73.1 ± 40.9 ml/min/1.73 m², respectively. After propensity score matching, the number of patients in both the DPP4i and control groups became equal, resulting in 7343 patients in each group (Table 1). Following the matching process, the differences in each parameter between the two groups were small or well matched, indicating successful matching and ensuring comparability between the groups.

**Table 1:** Baseline characteristics of enrolled participants.

	**Before matching**			**After matching**		
Group	AKD + DPP4i (*n* = 7 348)	Control (*n* = 229 417)	Standard difference	AKD + DPP4i (*n* = 7343)	Control (*n* = 7343)	Standard difference
Age at index						
Mean ± SD	66.2 ± 13.4	62.1 ± 15.8	0.283	66.2 ± 13.4	66.2 ± 13.3	0.003
Gender						
Male	3665 (49.9%)	118 257 (52.7%)	0.057	3664 (49.9%)	3658 (49.8%)	0.017
Race						
White	4387 (59.7%)	133 591 (59.6%)	0.003	4387 (59.7%)	4439 (60.5%)	0.014
Black or African American	1313 (17.9%)	43 895 (19.6%)	0.046	1313 (17.9%)	1273 (17.3%)	0.014
Hispanic or Latino	769 (10.5%)	22 066 (9.8%)	0.021	769 (10.5%)	622 (8.5%)	0.068
Comorbidities						
Ischemic heart diseases	3078 (41.9%)	76 409 (34.1%)	0.162	3078 (41.9%)	3074 (41.9%)	0.001
Cerebrovascular diseases	1710 (23.3%)	38 840 (17.3%)	0.149	1708 (23.3%)	1623 (22.1%)	0.033
Medication						
ACEi	3571 (44.8%)	75 177 (30.4%)	0.301	3236 (44.1%)	3241 (44.1%)	0.001
ARB	2097 (28.6%)	37 245 (16.6%)	0.289	2095 (28.5%)	2075 (28.3%)	0.006
Antiarrhythmics	3887 (52.9%)	97 090 (43.3%)	0.194	3886 (52.9%)	3621 (49.3%)	0.072
Aspirin	4274 (58.2%)	95 471 (42.6%)	0.316	4272 (58.2%)	4251 (57.9%)	0.006
Clopidogrel	1287 (17.5%)	25 562 (11.4%)	0.175	1286 (17.5%)	1147 (15.6%)	0.051
β-Blockers	4894 (66.6%)	115 704 (51.6%)	0.310	4892 (66.6%)	4652 (63.4%)	0.069
GLP-1 analogs	376 (5.1%)	6318 (2.8%)	0.118	375 (5.1%)	270 (3.7%)	0.070
SGLT-2 inhibitors	647 (8.8%)	5277 (2.3%)	0.285	646 (8.8%)	235 (3.2%)	0.246
Metformin	3637 (49.5%)	50 013 (21.8%)	0.605	3635 (49.5%)	1990 (27.1%)	0.474
Laboratory						
eGFR, ml/min/1.73 m^2^	70.5 ± 35.5	73.1 ± 40.9	0.067	70.5 ± 35.5	70.5 ± 38.2	0.001
BMI, kg/m^2^	30.9 ± 7.0	30.7 ± 7.3	0.025	30.9 ± 7.0	31.0 ± 7.2	0.016
Glucose	154.1 ± 63.1	133.6 ± 55.8	0.345	154.1 ± 63.1	133.6 ± 54.0	0.350

### Pre-specified outcomes

**Figure 1:**
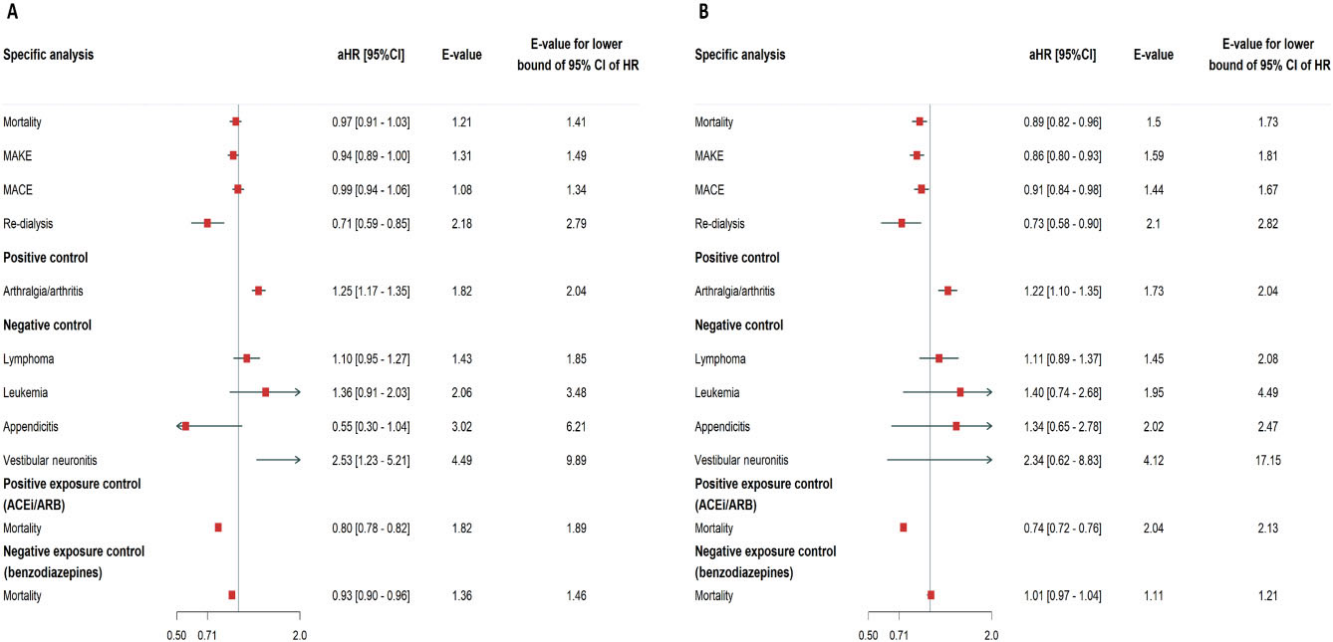
Pre-specified outcomes of DPP4i users compared with the control group before and after propensity score matching. The forest plots illustrate the adjusted HR of pre-specified outcomes of DPP4i use in DM patients after weaned-off AKI-requiring AKI. All-cause mortality, MAKEs, MACEs, and re-dialysis of DPP4i users had favorable outcomes compared with non-DPP4i users before (**A**) and after (**B**) 1:1 propensity score matching . Positive and negative controls, as well as positive exposure and negative exposure controls are also shown in the diagram.

**Figure 2:**
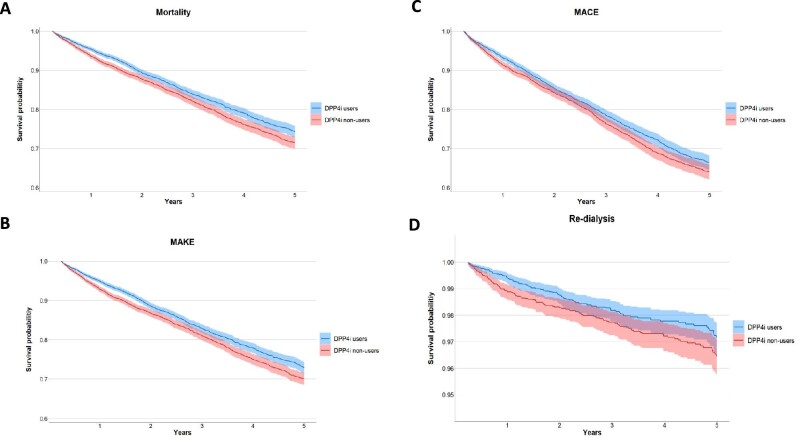
Kaplan–Meier curves of the pre-specified long-term outcomes of DPP4i users and the propensity score matching group. (**A**) All-cause mortality (log-rank *P* = .001). (**B**) MAKEs (log-rank *P* = .001). (**C**) MACEs (log-rank *P* = .022). (**D**) Re-dialysis (log-rank *P* = .034). The blue lines correspond to DPP4i users, while the red lines represent DPP4i non-users.

Within the defined outcomes, we conducted investigations of risk for mortality, MAKEs, MACEs, and re-dialysis within a duration ranging from 90 days after discharge up to 5 years. The overall cohort had a median duration of 2.4 years, bracketed by the 25th percentile at 3.7 years and the 75th at 1.2 years, with the 90th percentile reaching 0.7 years. The HRs and Kaplan–Meier curves illustrating the survival probabilities for the pre-specified outcomes associated with the use of DPP4i are presented in Figs 1 and 2, respectively. After the index date, DPP4i users had 25.7% all-cause mortality after 31 528 patient-years of follow-up, whereas DPP4i non-users had 28.5% all-cause mortality after 31 040 patient-years of follow-up. Utilizing DPP4i following discharge was linked to a decreased risk of all-cause mortality compared with the control group (aHR, 0.89; 95% CI, 0.82–0.96; E-value, 1.50) (Fig. 1, Supplementary Table S2). The utilization of DPP4i was linked to a reduced risk of MAKEs when compared with DPP4i non-users (aHR, 0.86; 95% CI, 0.80–0.93; E-value, 1.59) (Fig. 1, Supplementary Table S3). Moreover, we also examined MAKEs that necessitated dialysis intervention alone, and the use of DPP4is was associated with a decreased risk of re-dialysis (aHR, 0.73; 95% CI, 0.58–0.90; E-value, 2.10) (Fig. 1, Supplementary Table S4). Regarding cardiovascular complications, the group using DPP4is displayed a lower risk of MACEs (aHR, 0.91; 95% CI, 0.84–0.98; E-value, 1.44) (Fig. 1, Supplementary Table S5).

### Subgroup analysis

**Figure 3:**
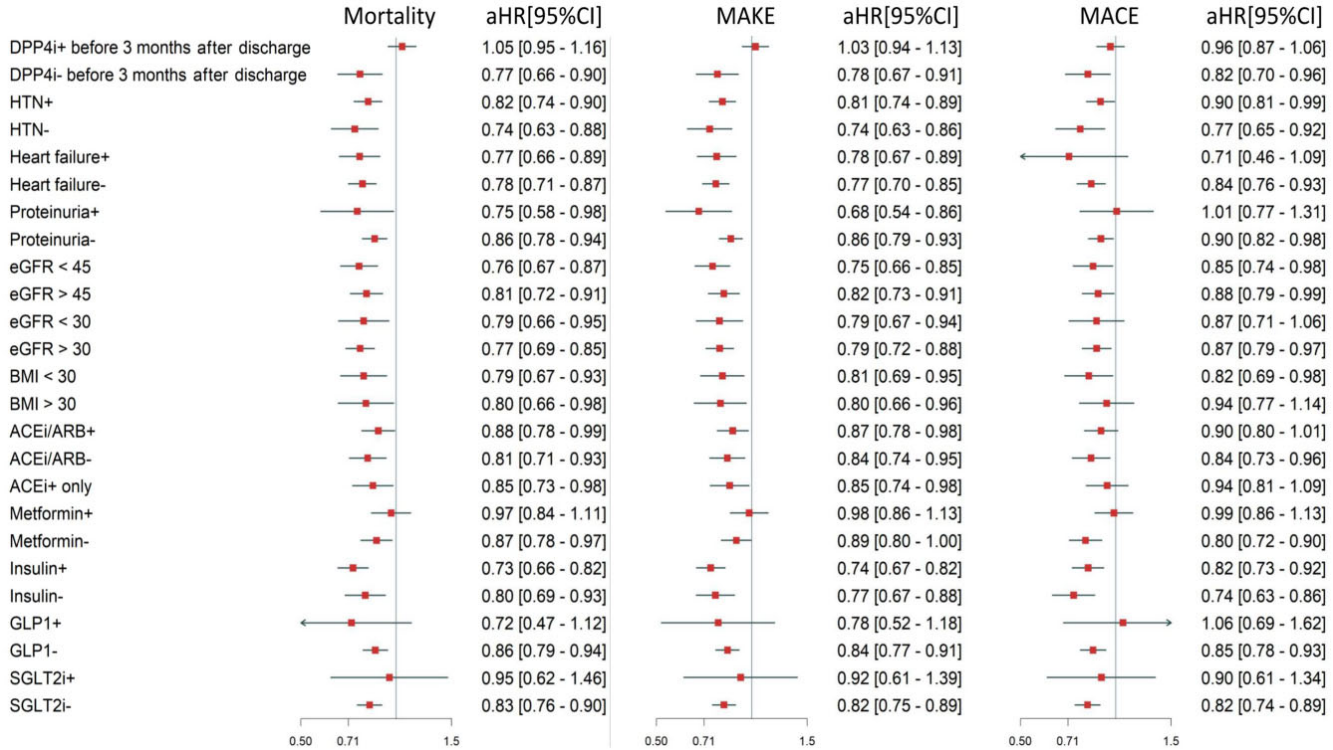
Subgroup analysis of pre-specified outcomes. The forest plots display the adjusted HRs of mortality, MAKEs, and MACEs within pre-specified subgroups of the DM patients after weaned-off dialysis-requiring AKI. HTN, hypertension; SGLT-2i, sodium–glucose cotransporter 2 inhibitor.

We performed a more in-depth analysis among patients who had not used a DPP4i before discharge. Among patients with AKD, new users were associated with a reduced risk of all-cause mortality (HR, 0.77; 95% CI, 0.66–0.90), MAKEs (HR, 0.78; 95% CI, 0.67–0.91), and MACEs (HR, 0.82; 95% CI, 0.70–0.96) (Fig. 3 and Supplementary Table S6). The utilization of DPP4is among diabetes patients with concurrent AKD was correlated with a lowered risk of mortality and MAKEs, specifically within predefined subgroups. Pertaining to MACEs, noteworthy enhancements were observed among individuals using DPP4is who had hypertension, without HF, a BMI <30 kg/m², without baseline kidney insufficiency (in terms of no proteinuria, or an eGFR surpassing 30 mL/min/1.73 m²), when contrasted with those not utilizing DPP4is (Fig. 3 and Supplementary Table S6). To minimize the effect of confounding medications on the outcomes, a subgroup analysis excluding concurrent medications and a logistic regression analysis (Supplementary Table S7) showed that DPP4is still had independently lower risks for adverse outcomes.

### Positive and negative controls

The DPP4i users had a higher risk of arthralgia/arthritis (aHR, 1.22; 95% CI, 1.10–1.35), as previously observed [20]. Subsequently, we applied a test set encompassing negative outcome controls (lymphoma, leukemia, appendicitis, and vestibular neuronitis) [21–23], where existing knowledge did not suggest any anticipated association. Consistent with our prior findings, the results unveiled no substantial association between the utilization of DPP4is in AKD patients and the specified negative outcome controls. We also conducted positive and negative exposure control analyses to scrutinize potential unmeasured confounding factors or systemic biases. In the positive exposure control, we contrasted individuals who used ACEis/ARBs within 90 days post-discharge, instead of DPP4is, and scrutinized the all-cause mortality following discharge (aHR, 0.74; 95% CI, 0.72–0.76). Conversely, within the negative exposure control group, we considered the use of benzodiazepines [24] as the exposure variable instead of DPP4is, which, as demonstrated, displayed no substantial association with all-cause mortality (aHR, 1.01; 95% CI, 0.97–1.04) (Fig. 1).

### External validation

Our results were further validated using 694 patients with type 2 diabetes concomitant with AKD in the CGRD database (Supplementary Figs S3 and S4). In line with our main finding, the DPP4i group with AKD had a significantly lower risk of mortality (HR, 0.72; 95% CI, 0.52–1.00), MAKEs (HR, 0.67; 95% CI, 0.51–0.88), MACEs (HR, 0.73; 95% CI, 0.56–0.96), and re-dialysis (HR, 0.62; 95% CI, 0.43–0.91) compared with the control group (Supplementary Figs S3 and S4).

## DISCUSSION

Our findings revealed that 28.5% of patients with type 2 diabetes and AKD who did not receive DPP4i treatment succumbed within 5 years after discontinuing dialysis due to AKI. Employing an extensive international real-world dataset, our findings underscore a decreased risk of all-cause mortality, MAKEs, MACEs, and associated with DPP4i use in AKD patients over a median follow-up of 2.4 years. Given escalating burdens of MACEs [2] and MAKEs [25] post-AKD, with AKD potentially exacerbating these conditions, especially in patients who were DPP4i new users, healthcare providers must consider DPP4is as part of a comprehensive strategy to address DM patients with concomitant AKD.

### DPP4i users associated with fewer MAKEs

In our current study, we specifically focused on patients who experienced the most severe AKI during hospitalization. A previous study indicated that DPP4i usage reduced adverse outcomes like mortality and end-stage kidney disease in diabetic patients after dialysis-requiring AKI [26]. In our current investigation, employing the global TriNetX platform, our findings highlight that DPP4i usage within 90 days post-discharge from this critical AKI phase was linked to a decreased risk of progressing to severe kidney issues, along with lower risks of all-cause mortality and MAKEs.

AKI transition to CKD is believed to be related to chronic inflammation and fibrotic pathways following kidney injury. DPP4is increase GLP-1, glucagon-like peptide-1 (GLP-1) levels, and GLP-1 receptors have been identified in various organs [27]. Additionally, DPP4is have demonstrated organ-protective effects through GLP-1-independent pathways [28], such as modulating the TGF-β signaling pathway and fibrotic factors [29]. A study by Katagiri *et al*. reported that a DPP4i had a reno-protective effect in rodent models of cisplatin-induced AKI by enhancing GLP-1 signaling [30]. DPP-4is could attenuate albuminuria and renal injury independent of their hypoglycemic effect [31]. Another study found that DPP4is can protect the kidney from diabetic nephropathy, ischemia–reperfusion injury, and chronic kidney disease [9]. Taken together, using DPP4is in DM patients with AKD could lower the risk of re-dialysis.

### DPP4i users associated with fewer MACEs

Using DPP4is could decrease the odds of all-cause mortality, newly diagnosed HF, and the risk of coronary artery bypass grafts [32]. Our subgroup analysis focusing on patients with HF revealed that DPP4i users exhibited a reduction in all-cause mortality and MAKEs but demonstrated stability in MACEs. Additionally, a specific analysis indicated that DPP4i users did not incur an increased risk of new-onset HF (Supplementary Table S8). Monami *et al*. conducted a meta-analysis of randomized controlled trials that indicated a lower risk of all-cause mortality and MACEs associated with DPP4i use [33]. DPP4is have been found to possess anti-inflammatory properties and reduce oxidative stress [34], and improve endothelial dysfunction [35]. For the aforementioned reasons, DPP4is could potentially confer beneficial effects on MACEs in CKD patients.

### Subgroup analysis

The outcomes of these assessments were concordant with the primary approach. Notably, distinct benefits were evident primarily among younger patients, who did not receive sodium-glucose co-transporter 2 (SGLT-2) inhibitors, metformin, GLP-1 receptor agonists, or RAAS blockers, particularly concerning MACEs.

Moreover, targeted scrutiny of outcome constituents unveiled substantial associations between DPP4i new users and a reduced susceptibility to various combinations of adverse events. Specifically addressing MACEs, favorable effects were evident among DPP4i users with a BMI <30 kg/m² or an eGFR >30 mL/min/1.73 m². These findings underscore the versatility of DPP4i as a comprehensive therapeutic modality for the intricate interplay of AKD and diabetes.

Despite considerable efforts to develop interventions for AKI, the clinical management of AKD remains challenging due to the persistent rise in the incidence of AKI [36] and the limited number of available treatments [37]. The promising role of DPP4is in improving the outcomes of patients with type 2 diabetes and AKD extends beyond glycemic control.

### Limitations

Our study had several limitations. Firstly, the retrospective and observational design of the study precluded randomization, leading to potential biases. We mitigated these biases through the use of validated outcome definitions and propensity score matching techniques to avoid systemic bias. We used variable positive and negative controls to mitigate the influence of latent confounders. Secondly, although we employed the code for DPP4is, we did not differentiate between different types and doses of DPP4is and their specific effects on the predefined outcomes. However, their effectivenesses seem to be similar [38].

Thirdly, another limitation of our study is the reliance on diagnostic codes for disease classification from an electronic dataset. This approach may have resulted in an underestimation of mild conditions or those that occur outside of the medical system, introducing potential ascertainment bias. The specificity test and external validation revealed no difference between patients who were DPP4i users or non-users in our negative control analysis, aiding in the removal of selection bias.

In light of these limitations, cautious interpretation of our findings is warranted, and further research is needed to validate and expand upon our observations.

## CONCLUSIONS

This study constitutes a comprehensive real-world analysis of extensive big data, wherein a noteworthy reduction in the risks of all-cause mortality, MAKEs, MACEs, and re-dialysis was ascertained among individuals contending with DM concurrent with AKD. These findings ardently endorse the incorporation of DPP4is into the therapeutic schema for DM patients following episodes of AKI necessitating dialysis.

## Supplementary Material

sfae385_Supplemental_File

## Data Availability

The datasets utilized and/or analyzed during the current study can be made available from the corresponding author upon reasonable request.
