# Supplementary material for: Dipeptidyl peptidase 4 inhibitors reduce the risk of adverse outcomes after acute kidney injury in diabetic patients
Source: Clin Kidney J. 2024 Dec 3;18(2):sfae385. doi: 10.1093/ckj/sfae385 (PMC11806628; doi:10.1093/ckj/sfae385)
Supplement: sfae385_Supplemental_File [file sfae385_supplemental_file.docx]

**Supplemental methods to “Dipeptidyl Peptidase 4 Inhibitors Reduced Risk of Adverse Outcomes in Diabetes Patients after Severe Acute Kidney Injury”**

1. Supplementary material and methods
2. Etiologies of AKI and Cohort of selected population
3. Outcome definition
4. Supplementary methods tables
5. Supplementary figures
6. **Material and methods**

Ethical approval (approval number: 11202-002) was obtained from the Institutional Review Board of Chi-Mei Hospital. Prior to enrollment in the study, written informed consent for clinical data collection and research use was obtained from all participants.

1. **TRINETX FEDERATED DATA NETWORK**

TriNetX was originally created to enhance the efficiency of collaborative industry-academia clinical trial research. Its primary aim was to empower researchers with the use of real-world data for designing trials that meet their accrual requirements. Additionally, it helps identify suitable performance sites to invite for trial participation. In 2015, TriNetX began approaching health care organizations (HCOs) with established i2b2 research repositories, inviting them to become data providers on their network. As time passed, TriNetX improved and evolved its data harmonization processes, eliminating the necessity for data sources to have an i2b2 repository. TriNetX follows a hub-and-spoke model, wherein Pharma and CRO sponsors pay a subscription fee to access aggregated counts from the HCOs in the network, which contain deidentified patient data. This business model has proven successful, attracting 14 prominent Pharma and CRO sponsors who have subscribed, along with a network of 75HCO data providers.[1, 2]

1. **NETWORK INFRASTRUCTURE**

TriNetX operates as a multitenant software-as-a-service platform built on the architecture of Amazon Web Services (AWS). The data from Health Care Organizations (HCOs) accessible through the TriNetX network is stored on an appliance situated at each HCO data center. During the onboarding process, the data is loaded onto the appliance using an extract-transform-load method, making use of the existing capabilities and scripting of the TriNetX agent. Apart from i2b2, TriNetX also supports the loading of data from various other source systems, combining its product and service capabilities to achieve this functionality.[1]

1. **SECURITY**

TriNetX is implemented on a secure virtual private cloud, hosted by AWS, that complies with the Health Insurance Portability and Accountability Act (HIPAA). This cloud infrastructure satisfies various industry-standard security certifications, including the Federal Risk and Authorization Management Program (FedRAMP), NIST 800-53, and others.To ensure secure access to TriNetX, Transport Layer Security (TLS) and a 2048-bit security certificate are employed. The services hosted behind AWS's Elastic Load Balancer are configured in accordance with the AWS Elastic Load Balancer Security Policy 2015-05.

(<https://aws.amazon.com/security/> )

The TriNetX appliance is designed with a strong emphasis on security, ensuring that there are no unnecessary processes running and maintaining a locked-down environment. All communication is initiated from within the appliance, minimizing potential vulnerabilities. To ensure the highest level of security, regular penetration and vulnerability tests are conducted on the hosted application environment. TriNetX members have access to expert attestation regarding the appliance's security, along with comprehensive documentation that provides detailed information..[1] TriNetX functions as a worldwide federated health research network, granting access to electronic medical records encompassing diagnoses, procedures, medications, laboratory values, and genomic information across a vast network of large healthcare organizations (HCOs). The data for this report was obtained from a collection of HCOs grouped under the network named "Research," which consists of 80 individual HCOs.

1. **CLINICAL DATA, CONTROLLED TERMINOLOGIES, AND SEMANTIC MAPPING**

TriNetX serves as an innovative healthcare network, offering demographic, diagnosis, procedure, medication, and laboratory data for research purposes. Recently, they have expanded their services to include tumor registry and molecular genomic data. Moreover, TriNetX plans to further enhance their offerings by incorporating vital signs and other observation data relevant to oncology and pulmonology in the near future. This comprehensive and real-world dataset of clinical data will prove invaluable in facilitating evidence-based research for the benefit of researchers. The data ingested by the TriNetX appliance varies in its origin, depending on the healthcare organization (HCO). Some HCOs directly extract data from their electronic health records (EHRs), while others utilize data warehouses with diverse common data models, such as i2b2 and observational health data sciences and informatics[3]. In the case of commercial EHRs, a plethora of proprietary code system standards and terminology standards are employed, which may vary by country. For instance, in the United States, the Clinical Modification version of the International Classification of Diseases (ICD), Tenth Revision, is utilized for coding procedures. However, other countries might have different coding standards for procedures, and there is no universally accepted standard. Proprietary drug data is incorporated by many EHRs, each with its own identifier for the same drug. Moreover, medications may be coded to national drug codes or anatomic therapeutic chemical codes, commonly used in European countries, or local codes. Additionally, standard codes like Logical Observation Identifiers Names and Codes (LOINCs) are rarely used in laboratory information systems at HCOs and commercial laboratories for test results.

1. **DATA QUALITY**

Ensuring data quality is a significant challenge when it comes to utilizing research data effectively, as it has the potential to compromise the validity of research outcomes[4]. Despite the exponential growth in the adoption of Electronic Health Records (EHRs) driven by federal incentives and meaningful use requirements, the quality of the data contained in them, and subsequently used for research, is still in need of improvement. Since EHRs are primarily designed for billing and patient care purposes, the data they provide may not be of the highest quality when used for research endeavors. To tackle this challenge, there is a requirement for a comprehensive data-quality framework and approach[5]. There is limited research on data quality, and it primarily centers on evaluating the quality of data within a single system or institution[6]. The focus is often on determining if the data is adequate for its main purpose, which is providing clinical care to patients. However, TriNetX has taken a significant step by developing a comprehensive methodology to assess the quality of the data it utilizes. This methodology, known as the four Cs—cleanliness, consistency, correctness, and completeness—takes into consideration the data extracted from the source systems. The data is then transformed, cleaned up, deduplicated, de-identified, optionally obfuscated, and semantically mapped to ensure its reliability and usefulness.

1. **ANALYSIS SPECIFICATIONS**

The Compare Outcomes Analytic provides four distinct types of analyses: Measure of Association, Survival, Number of Instances, and Lab Result Distribution. The first three analyses offer an option to "exclude patients with outcomes prior to the window." This option proves particularly valuable when examining chronic diseases, as patients who have already experienced the outcome are no longer at risk of developing it during the specified time window. When the "exclude patients with outcomes prior to the time window" option is left unchecked, all patients in the cohort will be included in the analysis, regardless of whether they had the outcome before the time window. However, if this option is selected, patients will be excluded if their medical records indicate that they had the outcome before the start of the time window. This exclusion will apply to all patients who had the outcome before the index event, and any patients who develop the outcome between the index event and the beginning of the time window will also be excluded if the time window starts some days after the index event.

1. **MEASURE OF ASSOCIATION ANALYSIS**

The Measure of Association Analysis evaluates the proportion of patients with a specific outcome. The output summary provides the number of patients in each cohort who meet the query criteria, the number of patients with the outcome in each cohort, and the risk of the outcome in each cohort. Additionally, the output includes the Risk Difference (the difference in risks between the PA cohort and the hypertensive cohort), the Risk Ratio (the ratio of risks between the PA cohort and hypertensive cohort), and the Odds Ratio (the ratio of odds between the PA cohort and hypertensive cohort).

1. **SURVIVAL ANALYSIS**

The Kaplan-Meier Analysis calculates the probability of the outcome at specific time intervals (in this analysis, daily time intervals are used). To accommodate patients who exit the cohort during the analysis period, a censoring mechanism is applied, removing these patients from the analysis after their last recorded event. The output summary provides the number of patients in each cohort (meeting the query criteria), the number of patients with the outcome within the time window, the median survival (the number of days when survival drops below 50%, indicated as "-" when survival remains above 50% during the time window), and the survival probability at the end of the window (the percentage of survival at the end of the time window). Additionally, the analysis employs the Log-Rank Test, Hazard Ratio, and a test for Proportionality.

1. **NUMBER OF INSTANCES ANALYSIS**

The Number of Instances Analysis is a method that calculates the frequency of an outcome within a specified time window. This analysis offers two settings: patients with zero instances and the definition of an instance.

When opting to exclude patients with zero instances, these patients are not considered in the calculations for mean number of instances, standard deviation, or median. The histogram, which displays the distribution of patients by the number of instances, will not have a bar for zero. Conversely, by including patients with zero instances, the mean, standard deviation, and median for the number of instances will encompass the entire patient population, including those with zero instances. The histogram will then include a bar for zero instances.

The definition of an instance impacts how the counts are analyzed. When selecting Date, each calendar date on which any of the terms selected in the outcome are recorded will be treated as one instance. For example, if the outcome is "Med A or Med B," and a patient has "Med A" on January 3, both medications on January 4, and "Med B" on January 6, then that patient is considered to have three instances, representing January 3, January 4, and January 6. It's essential to note that if an outcome occurs across multiple dates, only the start date is tracked for the purpose of counting instances. For instance, a patient who begins a hospital stay on January 1, ends on January 3, begins another stay on January 10, and ends on January 15, is considered to have two instances of the outcome.

On the other hand, selecting Visit as an instance will count any visit that includes the outcome as one instance, regardless of how many times it occurred. For instance, if a patient is administered an analgesic on each of the three days of an inpatient stay following an index event, these three administrations will represent only one instance because they are associated with the same visit.

The output summary comprises the count of patients in the cohort, the count of patients in the cohort who had the outcome within the time window, the mean, standard deviation, and median of the counts, and the median (1+ instances) when patients with zero instances are included in the analysis. Additionally, T-Test statistics, testing for the difference between the cohorts, are also included.

1. **LABORATORY RESULTS ANALYSIS**

During the analysis, only lab results relevant to the outcomes are considered. Additionally, the analysis takes into account only the most recent lab values within the specified time window.For numeric lab results, the outcome summary includes the count of patients in the cohort meeting the query criteria, the count of patients with the outcome within the time window, the mean, and the standard deviation of the lab values in the cohort. Moreover, T-Test statistics are provided to assess the differences between the cohorts. Regarding non-numeric lab results, the report presents the counts of Negatives, Positives, and Unknowns, and these percentages of the total counts are depicted in the form of a bar chart.

1. **Etiologies of AKI and Cohort of selected population**
2. **Presumptive etiologies of AKI**

**Table S1**

|  | All patients  (n=236765) | DPP4i group  (n=7,348) | Control group  (n=229,417) | P value |
| --- | --- | --- | --- | --- |
| Cardiogenic shock | 87695 (37.0%) | 3270 (44.5%) | 84425 (36.8%) | < 0.01 |
| Cardiorenal syndrome | 19449 (8.2%) | 867 (11.8%) | 18582 (8.1%) | < 0.01 |
| Sepsis without shock | 146287 (61.8 %) | 4967 (67.6%) | 141320 (61.6%) | < 0.01 |
| Septic shock | 28640 (12.1%) | 1110 (15.1%) | 27530 (12.0%) | < 0.01 |
| Hypovolemic shock | 15877 (6.7%) | 507 (6.9%) | 15370 (6.7%) | 0.46 |
| Obstructive uropathy | 8914 (3.8%) | 426 (5.8%) | 8488 (3.7%) | < 0.01 |
| Drug-related AKI or contrast nephropathy | 11586 (4.9%) | 345 (4.7%) | 11241 (4.9%) | 0.55 |
| Others* | 12822 (5.4%) | 434 (5.9%) | 12388 (5.4%) | 0.21 |

Abbreviation: AKI, acute kidney injury; DPP4i, dipeptidyl peptidase 4 inhibitor

*Others: hypertension crisis, postpartum AKI, etc.

1. **Selected of studied population**

**Query Criteria for AKD with DPP4i patients**

This query was run on the network Research with 75 HCO(s) queried and 75 HCO(s) responded.

| Ungrouped terms | | | | | |
| --- | --- | --- | --- | --- | --- |
|  | must have |  | demographics | Age | Age (between 18 and 90 years (most recent occurrence)) |
| Group 1 | | | | | |
|  | **Group 1A Discharge** | | | | |
|  | must have | any of | procedure | UMLS:CPT:99217 | Observation care discharge day management (This code is to be utilized to report all services provided to a patient on discharge from outpatient hospital "observation status" if the discharge is on other than the initial date of "observation status." To report services to a patient designated as "observation status" or "inpatient status" and discharged on the same date, use the codes for Observation or Inpatient Care Services [including Admission and Discharge Services, 99234-99236 as appropriate.]) |
|  |  |  | procedure | UMLS:CPT:1013682 | Hospital Discharge Services |
|  |  |  | procedure | UMLS:CPT:99238 | Hospital discharge day management; 30 minutes or less |
|  |  |  | procedure | UMLS:CPT:99239 | Hospital discharge day management; more than 30 minutes |
|  | date constraint | | The terms in this group occurred on or before Sep 30, 2022 | | |
|  | event relationship | | Any instance of dialysis occurred within 3 months before or up to 1 day after any instance of Discharge | | |
|  | **Group 1B dialysis** | | | | |
|  | must have | any of | procedure | UMLS:CPT:90937 | Hemodialysis procedure requiring repeated evaluation(s) with or without substantial revision of dialysis prescription |
|  |  |  | procedure | UMLS:CPT:90947 | Dialysis procedure other than hemodialysis (eg, peritoneal dialysis, hemofiltration, or other continuous renal replacement therapies) requiring repeated evaluations by a physician or other qualified health care professional, with or without substantial revision of dialysis prescription |
|  |  |  | procedure | UMLS:CPT:1012752 | Hemodialysis Procedures |
|  |  |  | diagnosis | UMLS:ICD10CM:E87 | Other disorders of fluid, electrolyte and acid-base balance |
|  |  |  | procedure | UMLS:CPT:90935 | Hemodialysis procedure with single evaluation by a physician or other qualified health care professional |
|  |  |  | procedure | UMLS:CPT:90945 | Dialysis procedure other than hemodialysis (eg, peritoneal dialysis, hemofiltration, or other continuous renal replacement therapies), with single evaluation by a physician or other qualified health care professional |
|  |  |  | procedure | UMLS:CPT:1006747 | Hemodialysis Access, Intervascular Cannulation for Extracorporeal Circulation, or Shunt Insertion Procedures on Arteries and Veins |
|  |  |  | procedure | UMLS:SNOMED:302497006 | Hemodialysis |
|  |  |  | procedure | UMLS:ICD9CM:39.95 | Hemodialysis |
|  |  |  | diagnosis | UMLS:ICD10CM:Z99.2 | Dependence on renal dialysis |
|  |  |  | procedure | UMLS:HCPCS:C1752 | Catheter, hemodialysis/peritoneal, short-term |
|  |  |  | procedure | UMLS:CPT:1012740 | Dialysis Services and Procedures |
|  |  | and | diagnosis | UMLS:ICD10CM:E11 | Type 2 diabetes mellitus |
| Group 2 | | | | | |
|  | **Group 2A Discharge** | | | | |
|  | must have | any of | procedure | UMLS:CPT:99217 | Observation care discharge day management (This code is to be utilized to report all services provided to a patient on discharge from outpatient hospital "observation status" if the discharge is on other than the initial date of "observation status." To report services to a patient designated as "observation status" or "inpatient status" and discharged on the same date, use the codes for Observation or Inpatient Care Services [including Admission and Discharge Services, 99234-99236 as appropriate.]) |
|  |  |  | procedure | UMLS:CPT:1013682 | Hospital Discharge Services |
|  |  |  | procedure | UMLS:CPT:99238 | Hospital discharge day management; 30 minutes or less |
|  |  |  | procedure | UMLS:CPT:99239 | Hospital discharge day management; more than 30 minutes |
|  | date constraint | | The terms in this group occurred at any time | | |
|  | event relationship | | Any instance of AKD occurred within 1 day and 3 months after any instance of Discharge | | |
|  | **Group 2B AKD** | | | | |
|  | must have |  | medication | NLM:ATC:A10BH | Dipeptidyl peptidase 4 (DPP-4) inhibitors |
|  | cannot have |  | diagnosis | UMLS:ICD10CM:R69 | Illness, unspecified |
|  |  | or | diagnosis | UMLS:ICD10CM:R99-R99 | Ill-defined and unknown cause of mortality (R99) |
|  |  | or | demographics | Deceased | Deceased |
|  |  | or | diagnosis | UMLS:ICD10CM:R99 | Ill-defined and unknown cause of mortality |
|  |  | or | procedure | UMLS:CPT:90937 | Hemodialysis procedure requiring repeated evaluation(s) with or without substantial revision of dialysis prescription |
|  |  | or | procedure | UMLS:CPT:90947 | Dialysis procedure other than hemodialysis (eg, peritoneal dialysis, hemofiltration, or other continuous renal replacement therapies) requiring repeated evaluations by a physician or other qualified health care professional, with or without substantial revision of dialysis prescription |
|  |  | or | procedure | UMLS:CPT:90945 | Dialysis procedure other than hemodialysis (eg, peritoneal dialysis, hemofiltration, or other continuous renal replacement therapies), with single evaluation by a physician or other qualified health care professional |
|  |  | or | procedure | UMLS:CPT:1006747 | Hemodialysis Access, Intervascular Cannulation for Extracorporeal Circulation, or Shunt Insertion Procedures on Arteries and Veins |
|  |  | or | procedure | UMLS:CPT:1012752 | Hemodialysis Procedures |
|  |  | or | diagnosis | UMLS:ICD10CM:E87 | Other disorders of fluid, electrolyte and acid-base balance |
|  |  | or | procedure | UMLS:ICD9CM:39.95 | Hemodialysis |
|  |  | or | procedure | UMLS:CPT:90935 | Hemodialysis procedure with single evaluation by a physician or other qualified health care professional |
|  |  | or | procedure | UMLS:SNOMED:302497006 | Hemodialysis |
|  |  | or | procedure | UMLS:CPT:1012740 | Dialysis Services and Procedures |
|  |  | or | procedure | UMLS:HCPCS:C1750 | Catheter, hemodialysis/peritoneal, long-term |
|  |  | or | diagnosis | UMLS:ICD10CM:Z99.2 | Dependence on renal dialysis |
|  |  | or | procedure | UMLS:HCPCS:C1752 | Catheter, hemodialysis/peritoneal, short-term |

**Query Criteria for AKD without DPP4i patients**

This query was run on the network Research with 75 HCO(s) queried and 75 HCO(s) responded.

| Ungrouped terms | | | | | |
| --- | --- | --- | --- | --- | --- |
|  | must have |  | demographics | Age | Age (between 18 and 90 years (most recent occurrence)) |
| Group 1 | | | | | |
|  | **Group 1A Discharge** | | | | |
|  | must have | any of | procedure | UMLS:CPT:99217 | Observation care discharge day management (This code is to be utilized to report all services provided to a patient on discharge from outpatient hospital "observation status" if the discharge is on other than the initial date of "observation status." To report services to a patient designated as "observation status" or "inpatient status" and discharged on the same date, use the codes for Observation or Inpatient Care Services [including Admission and Discharge Services, 99234-99236 as appropriate.]) |
|  |  |  | procedure | UMLS:CPT:1013682 | Hospital Discharge Services |
|  |  |  | procedure | UMLS:CPT:99238 | Hospital discharge day management; 30 minutes or less |
|  |  |  | procedure | UMLS:CPT:99239 | Hospital discharge day management; more than 30 minutes |
|  | date constraint | | The terms in this group occurred on or before Sep 30, 2022 | | |
|  | event relationship | | Any instance of dialysis occurred within 3 months before or up to 1 day after any instance of Discharge | | |
|  | **Group 1B dialysis** | | | | |
|  | must have | any of | procedure | UMLS:CPT:90937 | Hemodialysis procedure requiring repeated evaluation(s) with or without substantial revision of dialysis prescription |
|  |  |  | procedure | UMLS:CPT:90947 | Dialysis procedure other than hemodialysis (eg, peritoneal dialysis, hemofiltration, or other continuous renal replacement therapies) requiring repeated evaluations by a physician or other qualified health care professional, with or without substantial revision of dialysis prescription |
|  |  |  | procedure | UMLS:CPT:1012752 | Hemodialysis Procedures |
|  |  |  | diagnosis | UMLS:ICD10CM:E87 | Other disorders of fluid, electrolyte and acid-base balance |
|  |  |  | procedure | UMLS:CPT:90935 | Hemodialysis procedure with single evaluation by a physician or other qualified health care professional |
|  |  |  | procedure | UMLS:CPT:90945 | Dialysis procedure other than hemodialysis (eg, peritoneal dialysis, hemofiltration, or other continuous renal replacement therapies), with single evaluation by a physician or other qualified health care professional |
|  |  |  | procedure | UMLS:CPT:1006747 | Hemodialysis Access, Intervascular Cannulation for Extracorporeal Circulation, or Shunt Insertion Procedures on Arteries and Veins |
|  |  |  | procedure | UMLS:SNOMED:302497006 | Hemodialysis |
|  |  |  | procedure | UMLS:ICD9CM:39.95 | Hemodialysis |
|  |  |  | diagnosis | UMLS:ICD10CM:Z99.2 | Dependence on renal dialysis |
|  |  |  | procedure | UMLS:HCPCS:C1752 | Catheter, hemodialysis/peritoneal, short-term |
|  |  |  | procedure | UMLS:CPT:1012740 | Dialysis Services and Procedures |
|  |  | and | diagnosis | UMLS:ICD10CM:E11 | Type 2 diabetes mellitus |
| Group 2 | | | | | |
|  | **Group 2A Discharge** | | | | |
|  | must have | any of | procedure | UMLS:CPT:99217 | Observation care discharge day management (This code is to be utilized to report all services provided to a patient on discharge from outpatient hospital "observation status" if the discharge is on other than the initial date of "observation status." To report services to a patient designated as "observation status" or "inpatient status" and discharged on the same date, use the codes for Observation or Inpatient Care Services [including Admission and Discharge Services, 99234-99236 as appropriate.]) |
|  |  |  | procedure | UMLS:CPT:1013682 | Hospital Discharge Services |
|  |  |  | procedure | UMLS:CPT:99238 | Hospital discharge day management; 30 minutes or less |
|  |  |  | procedure | UMLS:CPT:99239 | Hospital discharge day management; more than 30 minutes |
|  | date constraint | | The terms in this group occurred at any time | | |
|  | event relationship | | Any instance of AKD occurred within 1 day and 3 months after any instance of Discharge | | |
|  | **Group 2B AKD** | | | | |
|  | cannot have |  | diagnosis | UMLS:ICD10CM:R69 | Illness, unspecified |
|  |  | or | diagnosis | UMLS:ICD10CM:R99-R99 | Ill-defined and unknown cause of mortality (R99) |
|  |  | or | demographics | Deceased | Deceased |
|  |  | or | diagnosis | UMLS:ICD10CM:R99 | Ill-defined and unknown cause of mortality |
|  |  | or | procedure | UMLS:CPT:90937 | Hemodialysis procedure requiring repeated evaluation(s) with or without substantial revision of dialysis prescription |
|  |  | or | procedure | UMLS:CPT:90947 | Dialysis procedure other than hemodialysis (eg, peritoneal dialysis, hemofiltration, or other continuous renal replacement therapies) requiring repeated evaluations by a physician or other qualified health care professional, with or without substantial revision of dialysis prescription |
|  |  | or | procedure | UMLS:CPT:90945 | Dialysis procedure other than hemodialysis (eg, peritoneal dialysis, hemofiltration, or other continuous renal replacement therapies), with single evaluation by a physician or other qualified health care professional |
|  |  | or | procedure | UMLS:CPT:1006747 | Hemodialysis Access, Intervascular Cannulation for Extracorporeal Circulation, or Shunt Insertion Procedures on Arteries and Veins |
|  |  | or | procedure | UMLS:CPT:1012752 | Hemodialysis Procedures |
|  |  | or | diagnosis | UMLS:ICD10CM:E87 | Other disorders of fluid, electrolyte and acid-base balance |
|  |  | or | procedure | UMLS:ICD9CM:39.95 | Hemodialysis |
|  |  | or | procedure | UMLS:CPT:90935 | Hemodialysis procedure with single evaluation by a physician or other qualified health care professional |
|  |  | or | procedure | UMLS:SNOMED:302497006 | Hemodialysis |
|  |  | or | procedure | UMLS:CPT:1012740 | Dialysis Services and Procedures |
|  |  | or | procedure | UMLS:HCPCS:C1750 | Catheter, hemodialysis/peritoneal, long-term |
|  |  | or | diagnosis | UMLS:ICD10CM:Z99.2 | Dependence on renal dialysis |
|  |  | or | procedure | UMLS:HCPCS:C1752 | Catheter, hemodialysis/peritoneal, short-term |
|  |  | or | medication | NLM:ATC:A10BH | Dipeptidyl peptidase 4 (DPP-4) inhibitors |

1. **Positive exposure control cohort**

**Query Criteria for DM+AKD+ACEi/ARB patients**

|  | | | | | |
| --- | --- | --- | --- | --- | --- |
| Group 1 | | | | | |
|  | **Group 1A Discharge** | | | | |
|  | must have | any of | procedure | UMLS:CPT:99217 | Observation care discharge day management (This code is to be utilized to report all services provided to a patient on discharge from outpatient hospital "observation status" if the discharge is on other than the initial date of "observation status." To report services to a patient designated as "observation status" or "inpatient status" and discharged on the same date, use the codes for Observation or Inpatient Care Services [including Admission and Discharge Services, 99234-99236 as appropriate.]) |
|  |  |  | procedure | UMLS:CPT:1013682 | Hospital Discharge Services |
|  |  |  | procedure | UMLS:CPT:99238 | Hospital discharge day management; 30 minutes or less |
|  |  |  | procedure | UMLS:CPT:99239 | Hospital discharge day management; more than 30 minutes |
|  | date constraint | | The terms in this group occurred at any time | | |
|  | event relationship | | Any instance of AKD occurred within 1 day and 3 months after any instance of Discharge | | |
|  | **Group 1B AKD** | | | | |
|  | must have | any of | medication | NLM:VA:CV800 | ACE INHIBITORS |
|  |  |  | medication | NLM:VA:CV805 | ANGIOTENSIN II INHIBITOR |
|  | cannot have |  | diagnosis | UMLS:ICD10CM:R69 | Illness, unspecified |
|  |  | or | diagnosis | UMLS:ICD10CM:R99-R99 | Ill-defined and unknown cause of mortality (R99) |
|  |  | or | demographics | Deceased | Deceased |
|  |  | or | diagnosis | UMLS:ICD10CM:R99 | Ill-defined and unknown cause of mortality |
|  |  | or | procedure | UMLS:CPT:90937 | Hemodialysis procedure requiring repeated evaluation(s) with or without substantial revision of dialysis prescription |
|  |  | or | procedure | UMLS:CPT:90947 | Dialysis procedure other than hemodialysis (eg, peritoneal dialysis, hemofiltration, or other continuous renal replacement therapies) requiring repeated evaluations by a physician or other qualified health care professional, with or without substantial revision of dialysis prescription |
|  |  | or | procedure | UMLS:CPT:90945 | Dialysis procedure other than hemodialysis (eg, peritoneal dialysis, hemofiltration, or other continuous renal replacement therapies), with single evaluation by a physician or other qualified health care professional |
|  |  | or | procedure | UMLS:CPT:1006747 | Hemodialysis Access, Intervascular Cannulation for Extracorporeal Circulation, or Shunt Insertion Procedures on Arteries and Veins |
|  |  | or | procedure | UMLS:CPT:1012752 | Hemodialysis Procedures |
|  |  | or | diagnosis | UMLS:ICD10CM:E87 | Other disorders of fluid, electrolyte and acid-base balance |
|  |  | or | procedure | UMLS:ICD9CM:39.95 | Hemodialysis |
|  |  | or | procedure | UMLS:CPT:90935 | Hemodialysis procedure with single evaluation by a physician or other qualified health care professional |
|  |  | or | procedure | UMLS:SNOMED:302497006 | Hemodialysis |
|  |  | or | procedure | UMLS:CPT:1012740 | Dialysis Services and Procedures |
|  |  | or | procedure | UMLS:HCPCS:C1750 | Catheter, hemodialysis/peritoneal, long-term |
|  |  | or | diagnosis | UMLS:ICD10CM:Z99.2 | Dependence on renal dialysis |
|  |  | or | procedure | UMLS:HCPCS:C1752 | Catheter, hemodialysis/peritoneal, short-term |

**Query Criteria for DM+AKD without ACEi/ARB patients**

|  | | | | | |
| --- | --- | --- | --- | --- | --- |
| Group 1 | | | | | |
|  | **Group 1A Discharge** | | | | |
|  | must have | any of | procedure | UMLS:CPT:99217 | Observation care discharge day management (This code is to be utilized to report all services provided to a patient on discharge from outpatient hospital "observation status" if the discharge is on other than the initial date of "observation status." To report services to a patient designated as "observation status" or "inpatient status" and discharged on the same date, use the codes for Observation or Inpatient Care Services [including Admission and Discharge Services, 99234-99236 as appropriate.]) |
|  |  |  | procedure | UMLS:CPT:1013682 | Hospital Discharge Services |
|  |  |  | procedure | UMLS:CPT:99238 | Hospital discharge day management; 30 minutes or less |
|  |  |  | procedure | UMLS:CPT:99239 | Hospital discharge day management; more than 30 minutes |
|  | date constraint | | The terms in this group occurred at any time | | |
|  | event relationship | | Any instance of AKD occurred within 1 day and 3 months after any instance of Discharge | | |
|  | **Group 1B AKD** | | | | |
|  | cannot have |  | diagnosis | UMLS:ICD10CM:R69 | Illness, unspecified |
|  |  | or | diagnosis | UMLS:ICD10CM:R99-R99 | Ill-defined and unknown cause of mortality (R99) |
|  |  | or | demographics | Deceased | Deceased |
|  |  | or | diagnosis | UMLS:ICD10CM:R99 | Ill-defined and unknown cause of mortality |
|  |  | or | procedure | UMLS:CPT:90937 | Hemodialysis procedure requiring repeated evaluation(s) with or without substantial revision of dialysis prescription |
|  |  | or | procedure | UMLS:CPT:90947 | Dialysis procedure other than hemodialysis (eg, peritoneal dialysis, hemofiltration, or other continuous renal replacement therapies) requiring repeated evaluations by a physician or other qualified health care professional, with or without substantial revision of dialysis prescription |
|  |  | or | procedure | UMLS:CPT:90945 | Dialysis procedure other than hemodialysis (eg, peritoneal dialysis, hemofiltration, or other continuous renal replacement therapies), with single evaluation by a physician or other qualified health care professional |
|  |  | or | procedure | UMLS:CPT:1006747 | Hemodialysis Access, Intervascular Cannulation for Extracorporeal Circulation, or Shunt Insertion Procedures on Arteries and Veins |
|  |  | or | procedure | UMLS:CPT:1012752 | Hemodialysis Procedures |
|  |  | or | diagnosis | UMLS:ICD10CM:E87 | Other disorders of fluid, electrolyte and acid-base balance |
|  |  | or | procedure | UMLS:ICD9CM:39.95 | Hemodialysis |
|  |  | or | procedure | UMLS:CPT:90935 | Hemodialysis procedure with single evaluation by a physician or other qualified health care professional |
|  |  | or | procedure | UMLS:SNOMED:302497006 | Hemodialysis |
|  |  | or | procedure | UMLS:CPT:1012740 | Dialysis Services and Procedures |
|  |  | or | procedure | UMLS:HCPCS:C1750 | Catheter, hemodialysis/peritoneal, long-term |
|  |  | or | diagnosis | UMLS:ICD10CM:Z99.2 | Dependence on renal dialysis |
|  |  | or | procedure | UMLS:HCPCS:C1752 | Catheter, hemodialysis/peritoneal, short-term |
|  |  | or | medication | NLM:VA:CV800 | ACE INHIBITORS |
|  |  | or | medication | NLM:VA:CV805 | ANGIOTENSIN II INHIBITOR |

1. **Negative exposure controls**

**Query Criteria for DM+AKD patients with benzodiazepines**

| Group 1 | | | | | |
| --- | --- | --- | --- | --- | --- |
|  | **Group 1A Discharge** | | | | |
|  | must have | any of | procedure | UMLS:CPT:99217 | Observation care discharge day management (This code is to be utilized to report all services provided to a patient on discharge from outpatient hospital "observation status" if the discharge is on other than the initial date of "observation status." To report services to a patient designated as "observation status" or "inpatient status" and discharged on the same date, use the codes for Observation or Inpatient Care Services [including Admission and Discharge Services, 99234-99236 as appropriate.]) |
|  |  |  | procedure | UMLS:CPT:1013682 | Hospital Discharge Services |
|  |  |  | procedure | UMLS:CPT:99238 | Hospital discharge day management; 30 minutes or less |
|  |  |  | procedure | UMLS:CPT:99239 | Hospital discharge day management; more than 30 minutes |
|  | date constraint | | The terms in this group occurred at any time | | |
|  | event relationship | | Any instance of AKD occurred within 1 day and 3 months after any instance of Discharge | | |
|  | **Group 1B AKD** | | | | |
|  | must have |  | medication | NLM:VA:CN302 | BENZODIAZEPINE DERIVATIVE SEDATIVES/HYPNOTICS |
|  | cannot have |  | diagnosis | UMLS:ICD10CM:R69 | Illness, unspecified |
|  |  | or | diagnosis | UMLS:ICD10CM:R99-R99 | Ill-defined and unknown cause of mortality (R99) |
|  |  | or | demographics | Deceased | Deceased |
|  |  | or | diagnosis | UMLS:ICD10CM:R99 | Ill-defined and unknown cause of mortality |
|  |  | or | procedure | UMLS:CPT:90937 | Hemodialysis procedure requiring repeated evaluation(s) with or without substantial revision of dialysis prescription |
|  |  | or | procedure | UMLS:CPT:90947 | Dialysis procedure other than hemodialysis (eg, peritoneal dialysis, hemofiltration, or other continuous renal replacement therapies) requiring repeated evaluations by a physician or other qualified health care professional, with or without substantial revision of dialysis prescription |
|  |  | or | procedure | UMLS:CPT:90945 | Dialysis procedure other than hemodialysis (eg, peritoneal dialysis, hemofiltration, or other continuous renal replacement therapies), with single evaluation by a physician or other qualified health care professional |
|  |  | or | procedure | UMLS:CPT:1006747 | Hemodialysis Access, Intervascular Cannulation for Extracorporeal Circulation, or Shunt Insertion Procedures on Arteries and Veins |
|  |  | or | procedure | UMLS:CPT:1012752 | Hemodialysis Procedures |
|  |  | or | diagnosis | UMLS:ICD10CM:E87 | Other disorders of fluid, electrolyte and acid-base balance |
|  |  | or | procedure | UMLS:ICD9CM:39.95 | Hemodialysis |
|  |  | or | procedure | UMLS:CPT:90935 | Hemodialysis procedure with single evaluation by a physician or other qualified health care professional |
|  |  | or | procedure | UMLS:SNOMED:302497006 | Hemodialysis |
|  |  | or | procedure | UMLS:CPT:1012740 | Dialysis Services and Procedures |
|  |  | or | procedure | UMLS:HCPCS:C1750 | Catheter, hemodialysis/peritoneal, long-term |
|  |  | or | diagnosis | UMLS:ICD10CM:Z99.2 | Dependence on renal dialysis |
|  |  | or | procedure | UMLS:HCPCS:C1752 | Catheter, hemodialysis/peritoneal, short-term |

**Query Criteria for DM+AKD patients without benzodiazipines**

| Group 1 | | | | | |
| --- | --- | --- | --- | --- | --- |
|  | **Group 1A Discharge** | | | | |
|  | must have | any of | procedure | UMLS:CPT:99217 | Observation care discharge day management (This code is to be utilized to report all services provided to a patient on discharge from outpatient hospital "observation status" if the discharge is on other than the initial date of "observation status." To report services to a patient designated as "observation status" or "inpatient status" and discharged on the same date, use the codes for Observation or Inpatient Care Services [including Admission and Discharge Services, 99234-99236 as appropriate.]) |
|  |  |  | procedure | UMLS:CPT:1013682 | Hospital Discharge Services |
|  |  |  | procedure | UMLS:CPT:99238 | Hospital discharge day management; 30 minutes or less |
|  |  |  | procedure | UMLS:CPT:99239 | Hospital discharge day management; more than 30 minutes |
|  | date constraint | | The terms in this group occurred at any time | | |
|  | event relationship | | Any instance of AKD occurred within 1 day and 3 months after any instance of Discharge | | |
|  | **Group 1B AKD** | | | | |
|  | cannot have |  | diagnosis | UMLS:ICD10CM:R69 | Illness, unspecified |
|  |  | or | diagnosis | UMLS:ICD10CM:R99-R99 | Ill-defined and unknown cause of mortality (R99) |
|  |  | or | demographics | Deceased | Deceased |
|  |  | or | diagnosis | UMLS:ICD10CM:R99 | Ill-defined and unknown cause of mortality |
|  |  | or | procedure | UMLS:CPT:90937 | Hemodialysis procedure requiring repeated evaluation(s) with or without substantial revision of dialysis prescription |
|  |  | or | procedure | UMLS:CPT:90947 | Dialysis procedure other than hemodialysis (eg, peritoneal dialysis, hemofiltration, or other continuous renal replacement therapies) requiring repeated evaluations by a physician or other qualified health care professional, with or without substantial revision of dialysis prescription |
|  |  | or | procedure | UMLS:CPT:90945 | Dialysis procedure other than hemodialysis (eg, peritoneal dialysis, hemofiltration, or other continuous renal replacement therapies), with single evaluation by a physician or other qualified health care professional |
|  |  | or | procedure | UMLS:CPT:1006747 | Hemodialysis Access, Intervascular Cannulation for Extracorporeal Circulation, or Shunt Insertion Procedures on Arteries and Veins |
|  |  | or | procedure | UMLS:CPT:1012752 | Hemodialysis Procedures |
|  |  | or | diagnosis | UMLS:ICD10CM:E87 | Other disorders of fluid, electrolyte and acid-base balance |
|  |  | or | procedure | UMLS:ICD9CM:39.95 | Hemodialysis |
|  |  | or | procedure | UMLS:CPT:90935 | Hemodialysis procedure with single evaluation by a physician or other qualified health care professional |
|  |  | or | procedure | UMLS:SNOMED:302497006 | Hemodialysis |
|  |  | or | procedure | UMLS:CPT:1012740 | Dialysis Services and Procedures |
|  |  | or | procedure | UMLS:HCPCS:C1750 | Catheter, hemodialysis/peritoneal, long-term |
|  |  | or | diagnosis | UMLS:ICD10CM:Z99.2 | Dependence on renal dialysis |
|  |  | or | procedure | UMLS:HCPCS:C1752 | Catheter, hemodialysis/peritoneal, short-term |
|  |  | or | medication | NLM:VA:CN302 | BENZODIAZEPINE DERIVATIVE SEDATIVES/HYPNOTICS |

1. **Outcome Definitions**

| Mortality | | | | |
| --- | --- | --- | --- | --- |
| Outcome definition | | | |  |
|  | Demographics | Deceased | Deceased | |
|  | Diagnosis | UMLS:ICD10CM:R99 | Ill-defined and unknown cause of mortality | |
|  | Diagnosis | UMLS:ICD10CM:R99-R99 | Ill-defined and unknown cause of mortality (R99) | |
| MAKEs | | | | |
| Outcome definition | | | |  |
|  | Demographics | Deceased | Deceased | |
|  | Diagnosis | UMLS:ICD10CM:R99 | Ill-defined and unknown cause of mortality | |
|  | Diagnosis | UMLS:ICD10CM:R99-R99 | Ill-defined and unknown cause of mortality (R99) | |
|  | Diagnosis | UMLS:ICD10CM:R69 | Illness, unspecified | |
|  | Procedure | UMLS:CPT:1012740 | Dialysis Services and Procedures | |
|  | Procedure | UMLS:CPT:90945 | Dialysis procedure other than hemodialysis (eg, peritoneal dialysis, hemofiltration, or other continuous renal replacement therapies), with single evaluation by a physician or other qualified health care professional | |
|  | Procedure | UMLS:CPT:1029674 | Dialysis Circuit Procedures | |
|  | Procedure | UMLS:CPT:90947 | Dialysis procedure other than hemodialysis (eg, peritoneal dialysis, hemofiltration, or other continuous renal replacement therapies) requiring repeated evaluations by a physician or other qualified health care professional, with or without substantial revision of dialysis prescription | |
| Re-dialysis | | | | |
| Outcome definition | | | |  |
|  | Procedure | UMLS:CPT:1012740 | Dialysis Services and Procedures | |
|  | Procedure | UMLS:CPT:90945 | Dialysis procedure other than hemodialysis (eg, peritoneal dialysis, hemofiltration, or other continuous renal replacement therapies), with single evaluation by a physician or other qualified health care professional | |
|  | Procedure | UMLS:CPT:1029674 | Dialysis Circuit Procedures | |
|  | Procedure | UMLS:CPT:90947 | Dialysis procedure other than hemodialysis (eg, peritoneal dialysis, hemofiltration, or other continuous renal replacement therapies) requiring repeated evaluations by a physician or other qualified health care professional, with or without substantial revision of dialysis prescription | |
| MACEs | | | | |
| Outcome definition | | | |  |
|  | Diagnosis | UMLS:ICD10CM:I46 | Cardiac arrest | |
|  | Diagnosis | UMLS:ICD10CM:I63 | Cerebral infarction | |
|  | Diagnosis | UMLS:ICD10CM:I61 | Nontraumatic intracerebral hemorrhage | |
|  | Diagnosis | UMLS:ICD10CM:I62 | Other and unspecified nontraumatic intracranial hemorrhage | |
|  | Diagnosis | UMLS:ICD10CM:I63.5 | Cerebral infarction due to unspecified occlusion or stenosis of cerebral arteries | |
|  | Diagnosis | UMLS:ICD10CM:I21.02 | ST elevation (STEMI) myocardial infarction involving left anterior descending coronary artery | |
|  | Diagnosis | UMLS:ICD10CM:I21.9 | Acute myocardial infarction, unspecified | |
|  | Diagnosis | UMLS:ICD10CM:I21.19 | ST elevation (STEMI) myocardial infarction involving other coronary artery of inferior wall | |
|  | Diagnosis | UMLS:ICD10CM:I21 | Acute myocardial infarction | |
|  | Diagnosis | UMLS:ICD10CM:I21.09 | ST elevation (STEMI) myocardial infarction involving other coronary artery of anterior wall | |
|  | Diagnosis | UMLS:ICD10CM:I21.2 | ST elevation (STEMI) myocardial infarction of other sites | |
|  | Diagnosis | UMLS:ICD10CM:I21.29 | ST elevation (STEMI) myocardial infarction involving other sites | |
|  | Diagnosis | UMLS:ICD10CM:I21.21 | ST elevation (STEMI) myocardial infarction involving left circumflex coronary artery | |
|  | Diagnosis | UMLS:ICD10CM:I21.11 | ST elevation (STEMI) myocardial infarction involving right coronary artery | |
|  | Procedure | UMLS:SNOMED:281572005 | Direct current cardiac shock | |
|  | Demographics | Deceased | Deceased | |

| arthritis/arthralgia | | | | |
| --- | --- | --- | --- | --- |
| Outcome definition | | | |  |
|  | Diagnosis | UMLS:ICD10CM:M12 | Other and unspecified arthropathy | |
|  | Diagnosis | UMLS:ICD10CM:M25.5 | Pain in joint | |
|  | Diagnosis | UMLS:ICD10CM:M25.50 | Pain in unspecified joint | |

| Lymphoma | | | | |
| --- | --- | --- | --- | --- |
|  | **Outcome definition** | | | |
|  | | Diagnosis | UMLS:ICD10CM:C81-C96 | Malignant neoplasms of lymphoid, hematopoietic and related tissue |
|  | | Diagnosis | UMLS:ICD10CM:C85 | Other specified and unspecified types of non-Hodgkin lymphoma |
|  | | Diagnosis | UMLS:ICD10CM:C85.8 | Other specified types of non-Hodgkin lymphoma |

| Leukemia | | | | |
| --- | --- | --- | --- | --- |
| Outcome definition | | | |  |
|  | Diagnosis | UMLS:ICD10CM:C95 | Leukemia of unspecified cell type | |
|  | Diagnosis | UMLS:ICD10CM:C95.9 | Leukemia, unspecified | |
|  | Diagnosis | UMLS:ICD10CM:C95.90 | Leukemia, unspecified not having achieved remission | |
|  | Diagnosis | UMLS:ICD10CM:C91 | Lymphoid leukemia | |
|  | Diagnosis | UMLS:ICD10CM:C92 | Myeloid leukemia | |

| Appendicitis | | | | |
| --- | --- | --- | --- | --- |
| Outcome definition | | | |  |
|  | Diagnosis | UMLS:ICD10CM:K37 | Unspecified appendicitis | |
|  | Diagnosis | UMLS:ICD10CM:K35.8 | Other and unspecified acute appendicitis | |
|  | Diagnosis | UMLS:ICD10CM:K35 | Acute appendicitis | |

| Vestibular neuroniitis | | | | |
| --- | --- | --- | --- | --- |
| Outcome definition | | | |  |
|  | Diagnosis | UMLS:ICD10CM:H81.2 | Vestibular neuronitis | |

1. **Supplementary methods tables**

Results are summarized in the table below. Analysis was performed on the cohorts after propensity score matching.

**Table S2**. **All-cause mortality**

| **1 Mortality** | | | | | | | | |
| --- | --- | --- | --- | --- | --- | --- | --- | --- |
| **Risk analysis excluding patients with outcome prior to the time window** | | | | | | | |  |
| Patients in cohort | Patients with outcome | Risk | | | |  |  |  |
| 6,983 | 1,033 | 0.148 | | | |  |  |  |
| 6,881 | 1,085 | 0.158 | | | |  |  |  |
|  | | | | | | | | |
|  | 95% CI | z | p |  |  |  |  |  |
| -0.010 | (-0.022, 0.002) | -1.595 | 0.111 |  |  |  |  |  |
| 0.938 | (0.867, 1.015) | N/A | N/A |  |  |  |  |  |
| 0.927 | (0.845, 1.017) | N/A | N/A |  |  |  |  |  |
|  | | | | | | | | |
| 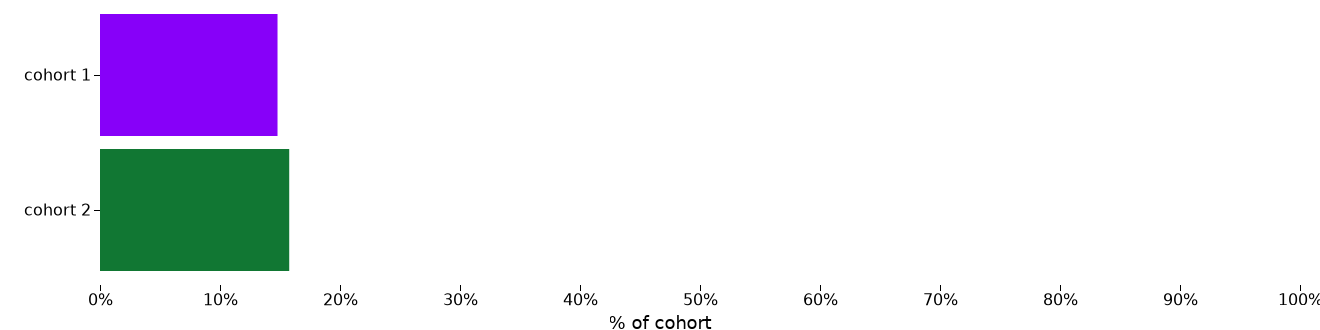 | | | | | | |  |  |
|  | | | | | | |  |  |
| **Kaplan - Meier survival analysis excluding patients with outcome prior to the time window** | | | | | | | |  |
| Patients in cohort | Patients with outcome | Median survival (days) | Survival probability at end of time window | | |  |  |  |
| 6,983 | 1,033 | -- | 74.29% | | |  |  |  |
| 6,881 | 1,085 | -- | 71.52% | | |  |  |  |
|  | | | | | | | | |
| χ^2^ | df | p |  |  |  |  |  |  |
| 11.130 | 1 | 0.001 |  |  |  |  |  |  |
|  | | | | | | | | |
| Hazard Ratio | 95% CI | χ^2^ | df | p | |  |  |  |
| 0.865 | (0.794, 0.942) | 2.385 | 1 | 0.123 | |  |  |  |
|  | | | | | | | | |
|  | | | | | | |  |  |
| **Number of instances excluding patients with outcome prior to the time window** | | | | | | | |  |
| Patients in cohort | Patients with outcome | Mean | Standard Deviation | Median | |  |  |  |
| 6,983 | 1,033 | 1.204 | 0.500 | 1 | |  |  |  |
| 6,881 | 1,085 | 1.183 | 0.404 | 1 | |  |  |  |
|  | | | | | | | | |
| t | df | p |  |  |  |  |  |  |
| 1.059 | 2116 | 0.290 |  |  |  |  |  |  |
|  | | | | | | | | |
| 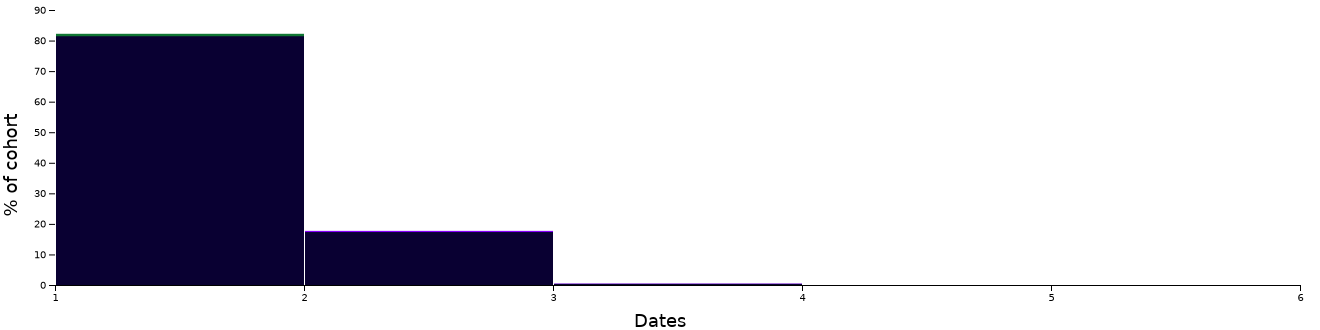 | | | | | | |  |  |
| **Table S3. MAKEs** | | | | | | |  |  |
| **2 MAKEs** | | | | | | | | |
| **Risk analysis** | | | | | | | |  |
| Patients in cohort | Patients with outcome | Risk | | | |  |  |  |
| 7,343 | 1,150 | 0.157 | | | |  |  |  |
| 7,343 | 1,193 | 0.162 | | | |  |  |  |
|  | | | | | | | | |
|  | 95% CI | z | p |  |  |  |  |  |
| -0.006 | (-0.018, 0.006) | -0.969 | 0.333 |  |  |  |  |  |
| 0.964 | (0.895, 1.038) | N/A | N/A |  |  |  |  |  |
| 0.957 | (0.876, 1.046) | N/A | N/A |  |  |  |  |  |
|  | | | | | | | | |
| 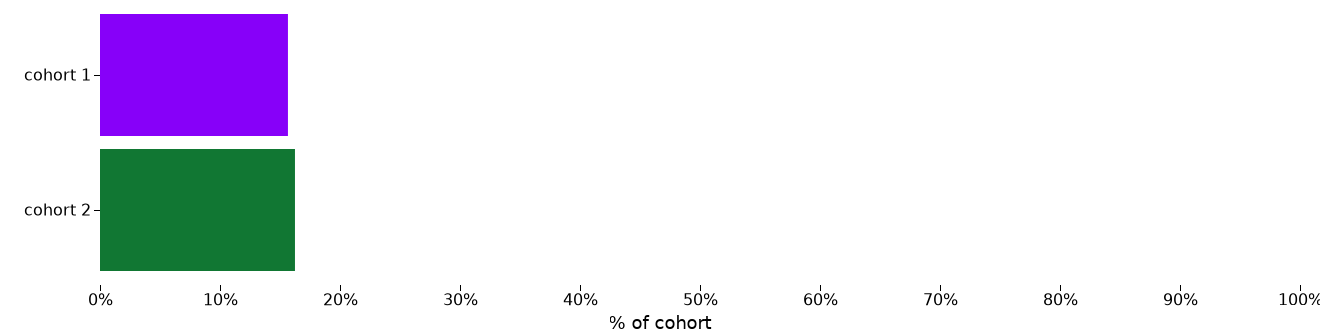 | | | | | | |  |  |
| **Kaplan - Meier survival analysis** | | | | | | | |  |
| Patients in cohort | Patients with outcome | Median survival (days) | Survival probability at end of time window | | |  |  |  |
| 7,343 | 1,150 | -- | 72.90% | | |  |  |  |
| 7,343 | 1,193 | -- | 70.08% | | |  |  |  |
|  | | | | | | | | |
| χ^2^ | df | p |  |  |  |  |  |  |
| 12.061 | 1 | 0.001 |  |  |  |  |  |  |
|  | | | | | | | | |
| Hazard Ratio | 95% CI | χ^2^ | df | p | |  |  |  |
| 0.866 | (0.799, 0.939) | 3.108 | 1 | 0.078 | |  |  |  |
|  | | | | | | | | |
| **Table S4. Re-dialysis** | | | | | | |  |  |
| **3 Re-dialysis** | | | | | | |  |  |

| **Risk analysis** | | | | | | | |  |
| --- | --- | --- | --- | --- | --- | --- | --- | --- |
| Patients in cohort | Patients with outcome | Risk | | | |  |  |  |
| 7,343 | 112 | 0.015 | | | |  |  |  |
| 7,343 | 131 | 0.018 | | | |  |  |  |
|  | | | | | | | | |
|  | 95% CI | z | p |  |  |  |  |  |
| -0.003 | (-0.007, 0.002) | -1.229 | 0.219 |  |  |  |  |  |
| 0.855 | (0.666, 1.098) | N/A | N/A |  |  |  |  |  |
| 0.853 | (0.661, 1.100) | N/A | N/A |  |  |  |  |  |
|  | | | | | | | | |
| 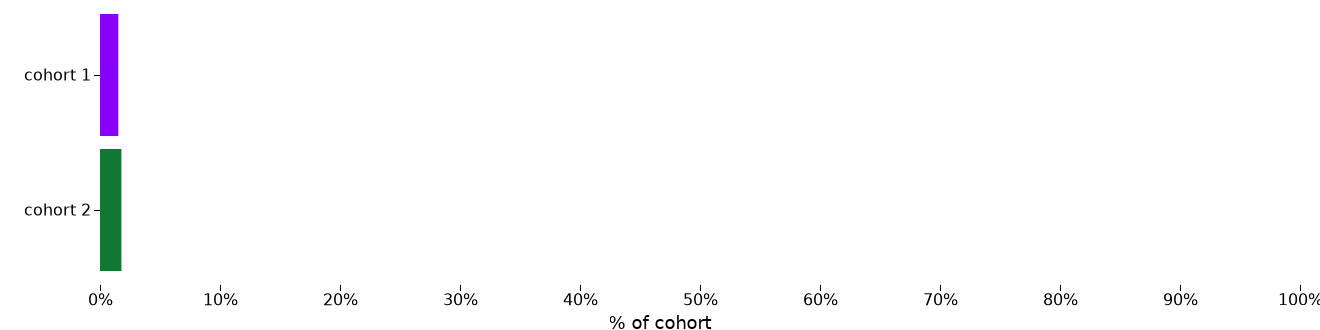 | | | | | | |  |  |
| **Kaplan - Meier survival analysis** | | | | | | | |  |
| Patients in cohort | Patients with outcome | Median survival (days) | Survival probability at end of time window | | |  |  |  |
| 7,343 | 112 | -- | 97.16% | | |  |  |  |
| 7,343 | 131 | -- | 96.47% | | |  |  |  |
|  | | | | | | | | |
| χ^2^ | df | p |  |  |  |  |  |  |
| 4.520 | 1 | 0.034 |  |  |  |  |  |  |
|  | | | | | | | | |
| Hazard Ratio | 95% CI | χ^2^ | df | p | |  |  |  |
| 0.761 | (0.591, 0.980) | 1.251 | 1 | 0.263 | |  |  |  |
|  | | | | | | | | |
|  | | | | | | |  |  |
| **Number of instances** | | | | | | | |  |
| Patients in cohort | Patients with outcome | Mean | Standard Deviation | Median | |  |  |  |
| 7,343 | 112 | 6.402 | 18.468 | 3 | |  |  |  |
| 7,343 | 131 | 4.412 | 7.197 | 3 | |  |  |  |
|  | | | | | | | | |
| t | df | p |  |  |  |  |  |  |
| 1.137 | 241 | 0.257 |  |  |  |  |  |  |
|  | | | | | | | | |
| 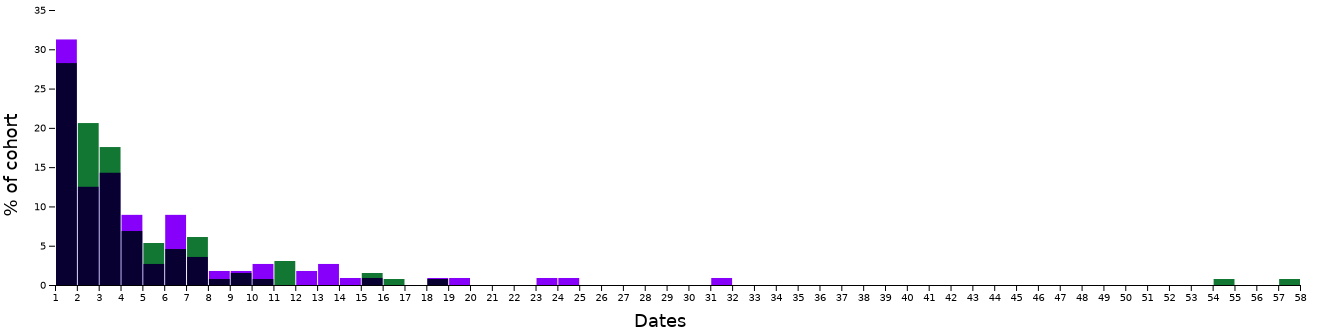 | | | | | | |  |  |
|  | | | | | | |  |  |
| **Table S5. MACEs** | | | | | | |  |  |
| **4 MACEs** | | | | | | | | |
| **Risk analysis excluding patients with outcome prior to the time window** | | | | | | | |  |
| Patients in cohort | Patients with outcome | Risk | | | |  |  |  |
| 4,954 | 999 | 0.202 | | | |  |  |  |
| 5,044 | 1,037 | 0.206 | | | |  |  |  |
|  | | | | | | | | |
|  | 95% CI | z | p |  |  |  |  |  |
| -0.004 | (-0.020, 0.012) | -0.489 | 0.625 |  |  |  |  |  |
| 0.981 | (0.908, 1.060) | N/A | N/A |  |  |  |  |  |
| 0.976 | (0.885, 1.076) | N/A | N/A |  |  |  |  |  |
|  | | | | | | | | |
| 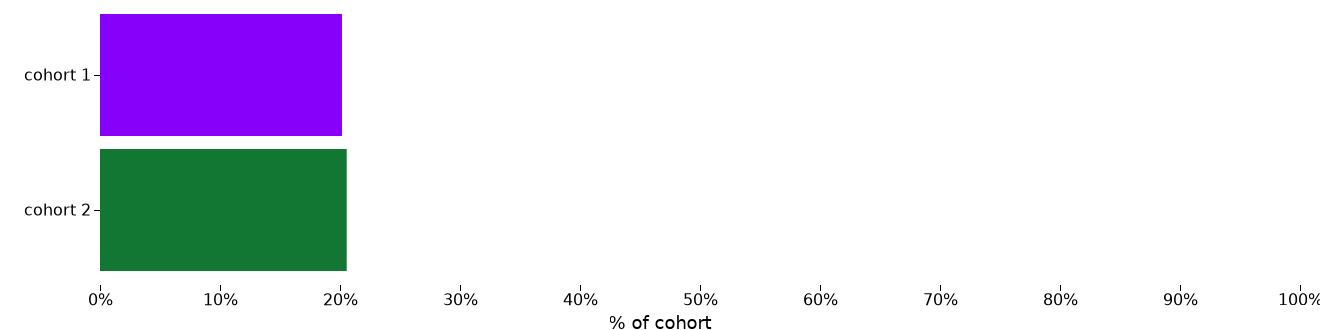 | | | | | | |  |  |
|  | | | | | | |  |  |
| **Kaplan - Meier survival analysis excluding patients with outcome prior to the time window** | | | | | | | |  |
| Patients in cohort | Patients with outcome | Median survival (days) | Survival probability at end of time window | | |  |  |  |
| 4,954 | 999 | -- | 66.42% | | |  |  |  |
| 5,044 | 1,037 | -- | 64.07% | | |  |  |  |
|  | | | | | | | | |
| χ^2^ | df | p |  |  |  |  |  |  |
| 5.270 | 1 | 0.022 |  |  |  |  |  |  |
|  | | | | | | | | |
| Hazard Ratio | 95% CI | χ^2^ | df | p | |  |  |  |
| 0.903 | (0.828, 0.985) | 0.895 | 1 | 0.344 | |  |  |  |
|  | | | | | | | | |
|  | | | | | | |  |  |
|  | | | | | | |  |  |
| **Number of instances excluding patients with outcome prior to the time window** | | | | | | | |  |
| Patients in cohort | Patients with outcome | Mean | Standard Deviation | Median | |  |  |  |
| 4,954 | 999 | 3.085 | 8.623 | 1 | |  |  |  |
| 5,044 | 1,037 | 2.637 | 5.837 | 1 | |  |  |  |
|  | | | | | | | | |
| t | df | p |  |  |  |  |  |  |
| 1.376 | 2034 | 0.169 |  |  |  |  |  |  |
|  | | | | | | | | |
| 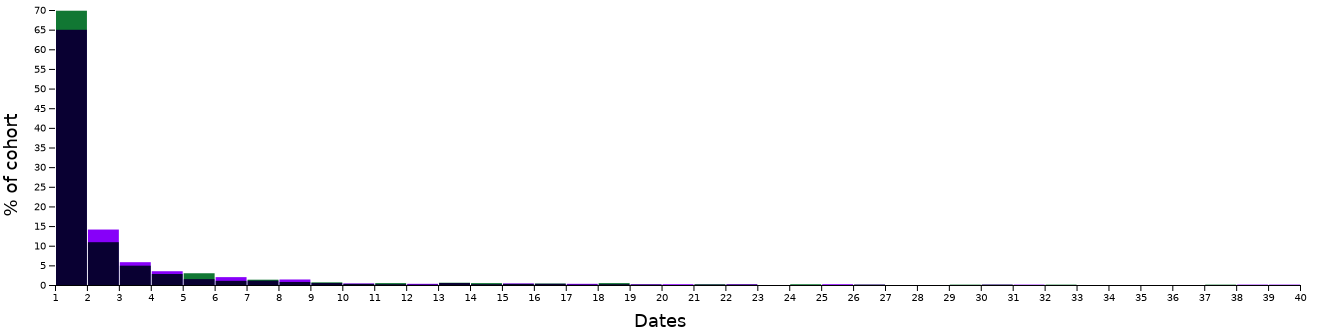 | | | | | | |  |  |
| **Table S6. Subgroup analysis**  **Ever-users**   \| **1 Mortality** \| \| \| \| \| \| \| \| \| \| \| \| \| \| \| \| \| --- \| --- \| --- \| --- \| --- \| --- \| --- \| --- \| --- \| --- \| --- \| --- \| --- \| --- \| --- \| --- \| \| **Risk analysis excluding patients with outcome prior to the time window** \| \| \| \| \| \| \| \| \| \| \| \| \| \| \|  \| \| Cohort \| \| \| Patients in cohort \| \| Patients with outcome \| \| Risk \| \| \| \| \| \| \|  \|  \| \| 1 \| DM+AKD+DPP4i with DPP4i usage 3 months before discharge \| \| 5,248 \| \| 821 \| \| 0.156 \| \| \| \| \| \| \|  \|  \| \| 2 \| DM+AKD without DPP4i with DPP4i usage before 3 months discharge \| \| 5,221 \| \| 733 \| \| 0.140 \| \| \| \| \| \| \|  \| \| \| \| \| \| \| \| \| \| \| \| \| \| \| \| \|  \|  \| \|  \| \| \|  \| \| 95% CI \| \| z \| p \|  \|  \| \| \| \|  \|  \| \| **Risk Difference** \| \| \| 0.016 \| \| (0.002, 0.030) \| \| 2.309 \| 0.021 \|  \|  \| \| \| \|  \|  \| \| **Risk Ratio** \| \| \| 1.114 \| \| (1.016, 1.222) \| \| N/A \| N/A \|  \|  \| \| \| \|  \|  \| \| **Odds Ratio** \| \| \| 1.135 \| \| (1.019, 1.265) \| \| N/A \| N/A \|  \|  \| \| \| \|  \| \| \| \| \| \| \| \| \| \| \| \| \| \| \| \| \| **Kaplan - Meier survival analysis excluding patients with outcome prior to the time window** \| \| \| \| \| \| \| \| \| \| \| \| \| \| \|  \|  \| \| Cohort \| \| \| Patients in cohort \| \| Patients with outcome \| \| Median survival (days) \| Survival probability at end of time window \| \| \| \| \| \|  \|  \| \| 1 \| DM+AKD+DPP4i with DPP4i usage 3 months before discharge \| \| 5,248 \| \| 821 \| \| -- \| 73.36% \| \| \| \| \| \|  \|  \| \| 2 \| DM+AKD without DPP4i with DPP4i usage before 3 months discharge \| \| 5,221 \| \| 733 \| \| -- \| 74.77% \| \| \| \| \| \|  \| \| \| \| \| \| \| \| \| \| \| \| \| \| \| \| \|  \|  \| \|  \| \| \| χ^2^ \| \| df \| \| p \|  \|  \|  \| \| \| \|  \|  \| \| **Log-Rank Test** \| \| \| 0.770 \| \| 1 \| \| 0.380 \|  \|  \|  \| \| \| \|  \| \| \| \| \| \| \| \| \| \| \| \| \| \| \| \| \|  \|  \| \|  \| \| \| Hazard Ratio \| \| 95% CI \| \| χ^2^ \| df \| p \| \| \| \| \|  \|  \| \| **Hazard Ratio and Proportionality** \| \| \| 1.046 \| \| (0.946, 1.155) \| \| 0.948 \| 1 \| 0.330 \| \| \| \| \|  \| \| \| \| \| \| \| \| \| \| \| \| \| \| \| \| \| **Number of instances excluding patients with outcome prior to the time window** \| \| \| \| \| \| \| \| \| \| \| \| \| \| \|  \|  \| \| Cohort \| \| \| Patients in cohort \| \| Patients with outcome \| \| Mean \| Standard Deviation \| Median \| \| \| \| \|  \|  \| \| 1 \| DM+AKD+DPP4i with DPP4i usage 3 months before discharge \| \| 5,248 \| \| 821 \| \| 1.200 \| 0.592 \| 1 \| \| \| \| \|  \|  \| \| 2 \| DM+AKD without DPP4i with DPP4i usage before 3 months discharge \| \| 5,221 \| \| 733 \| \| 1.150 \| 0.387 \| 1 \| \| \| \| \|  \| \| \| \| \| \| \| \| \| \| \| \| \| \| \| \| \|  \|  \| \|  \| \| \| t \| \| df \| \| p \|  \|  \|  \| \| \| \|  \|  \| \| **Test Statistics** \| \| \| 1.934 \| \| 1552 \| \| 0.053 \|  \|  \|  \| \| \| \|  \| \| \| \| \| \| \| \| \| \| \| \| \| \| \| \| \| **2 MAKE** \| \| \| \| \| \| \| \| \| \| \| \| \| \| \| \| \| **Risk analysis** \| \| \| \| \| \| \| \| \| \| \| \| \| \| \|  \| \| Cohort \| \| \| Patients in cohort \| \| Patients with outcome \| \| Risk \| \| \| \| \| \| \|  \|  \| \| 1 \| DM+AKD+DPP4i with DPP4i usage 3 months before discharge \| \| 5,493 \| \| 911 \| \| 0.166 \| \| \| \| \| \| \|  \|  \| \| 2 \| DM+AKD without DPP4i with DPP4i usage before 3 months discharge \| \| 5,493 \| \| 813 \| \| 0.148 \| \| \| \| \| \| \|  \| \| \| \| \| \| \| \| \| \| \| \| \| \| \| \| \|  \|  \| \|  \| \| \|  \| \| 95% CI \| \| z \| p \|  \|  \| \| \| \|  \|  \| \| **Risk Difference** \| \| \| 0.018 \| \| (0.004, 0.031) \| \| 2.571 \| 0.010 \|  \|  \| \| \| \|  \|  \| \| **Risk Ratio** \| \| \| 1.121 \| \| (1.027, 1.222) \| \| N/A \| N/A \|  \|  \| \| \| \|  \|  \| \| **Odds Ratio** \| \| \| 1.145 \| \| (1.033, 1.269) \| \| N/A \| N/A \|  \|  \| \| \| \|  \| \| \| \| \| \| \| \| \| \| \| \| \| \| \| \| \| **Kaplan - Meier survival analysis** \| \| \| \| \| \| \| \| \| \| \| \| \| \| \|  \|  \| \| Cohort \| \| \| Patients in cohort \| \| Patients with outcome \| \| Median survival (days) \| Survival probability at end of time window \| \| \| \| \| \|  \|  \| \| 1 \| DM+AKD+DPP4i with DPP4i usage 3 months before discharge \| \| 5,493 \| \| 911 \| \| -- \| 71.88% \| \| \| \| \| \|  \|  \| \| 2 \| DM+AKD without DPP4i with DPP4i usage before 3 months discharge \| \| 5,493 \| \| 813 \| \| -- \| 73.02% \| \| \| \| \| \|  \| \| \| \| \| \| \| \| \| \| \| \| \| \| \| \| \|  \|  \| \|  \| \| \| χ^2^ \| \| df \| \| p \|  \|  \|  \| \| \| \|  \|  \| \| **Log-Rank Test** \| \| \| 0.327 \| \| 1 \| \| 0.567 \|  \|  \|  \| \| \| \|  \| \| \| \| \| \| \| \| \| \| \| \| \| \| \| \| \|  \|  \| \|  \| \| \| Hazard Ratio \| \| 95% CI \| \| χ^2^ \| df \| p \| \| \| \| \|  \|  \| \| **Hazard Ratio and Proportionality** \| \| \| 1.028 \| \| (0.935, 1.130) \| \| 1.415 \| 1 \| 0.234 \| \| \| \| \|  \| \| \| \| \| \| \| \| \| \| \| \| \| \| \| \| \| **3 MACE** \| \| \| \| \| \| \| \| \| \| \| \| \| \| \| \| \| **Risk analysis excluding patients with outcome prior to the time window** \| \| \| \| \| \| \| \| \| \| \| \| \| \| \|  \| \| Cohort \| \| \| Patients in cohort \| \| Patients with outcome \| \| Risk \| \| \| \| \| \| \|  \|  \| \| 1 \| DM+AKD+DPP4i with DPP4i usage 3 months before discharge \| \| 3,635 \| \| 779 \| \| 0.214 \| \| \| \| \| \| \|  \|  \| \| 2 \| DM+AKD without DPP4i with DPP4i usage before 3 months discharge \| \| 3,814 \| \| 777 \| \| 0.204 \| \| \| \| \| \| \|  \| \| \| \| \| \| \| \| \| \| \| \| \| \| \| \| \|  \|  \| \|  \| \| \|  \| \| 95% CI \| \| z \| p \|  \|  \| \| \| \|  \|  \| \| **Risk Difference** \| \| \| 0.011 \| \| (-0.008, 0.029) \| \| 1.123 \| 0.261 \|  \|  \| \| \| \|  \|  \| \| **Risk Ratio** \| \| \| 1.052 \| \| (0.963, 1.149) \| \| N/A \| N/A \|  \|  \| \| \| \|  \|  \| \| **Odds Ratio** \| \| \| 1.066 \| \| (0.953, 1.192) \| \| N/A \| N/A \|  \|  \| \| \| \|  \| \| \| \| \| \| \| \| \| \| \| \| \| \| \| \| \| **Kaplan - Meier survival analysis excluding patients with outcome prior to the time window** \| \| \| \| \| \| \| \| \| \| \| \| \| \| \|  \|  \| \| Cohort \| \| \| Patients in cohort \| \| Patients with outcome \| \| Median survival (days) \| Survival probability at end of time window \| \| \| \| \| \|  \|  \| \| 1 \| DM+AKD+DPP4i with DPP4i usage 3 months before discharge \| \| 3,635 \| \| 779 \| \| -- \| 64.96% \| \| \| \| \| \|  \|  \| \| 2 \| DM+AKD without DPP4i with DPP4i usage before 3 months discharge \| \| 3,814 \| \| 777 \| \| -- \| 64.71% \| \| \| \| \| \|  \| \| \| \| \| \| \| \| \| \| \| \| \| \| \| \| \|  \|  \| \|  \| \| \| χ^2^ \| \| df \| \| p \|  \|  \|  \| \| \| \|  \|  \| \| **Log-Rank Test** \| \| \| 0.550 \| \| 1 \| \| 0.458 \|  \|  \|  \| \| \| \|  \| \| \| \| \| \| \| \| \| \| \| \| \| \| \| \| \|  \|  \| \|  \| \| \| Hazard Ratio \| \| 95% CI \| \| χ^2^ \| df \| p \| \| \| \| \|  \|  \| \| **Hazard Ratio and Proportionality** \| \| \| 0.963 \| \| (0.872, 1.064) \| \| 1.276 \| 1 \| 0.259 \| \| \| \| \|  \| \| \| \| \| \| \| \| \| \| \| \| \| \| \| \| \| **Number of instances excluding patients with outcome prior to the time window** \| \| \| \| \| \| \| \| \| \| \| \| \| \| \|  \|  \| \| Cohort \| \| \| Patients in cohort \| \| Patients with outcome \| \| Mean \| Standard Deviation \| Median \| \| \| \| \|  \|  \| \| 1 \| DM+AKD+DPP4i with DPP4i usage 3 months before discharge \| \| 3,635 \| \| 779 \| \| 3.118 \| 9.085 \| 1 \| \| \| \| \|  \|  \| \| 2 \| DM+AKD without DPP4i with DPP4i usage before 3 months discharge \| \| 3,814 \| \| 777 \| \| 2.353 \| 3.806 \| 1 \| \| \| \| \|  \| \| \| \| \| \| \| \| \| \| \| \| \| \| \| \| \|  \|  \| \|  \| \| \| t \| \| df \| \| p \|  \|  \|  \| \| \| \|  \|  \| \| **Test Statistics** \| \| \| 2.166 \| \| 1554 \| \| 0.030 \|  \|  \|  \| \| \| \|  \| \| \| \| \| \| \| \| \| \| \| \| \| \| \| \|   **New-users**   \| **1 Mortality** \| \| \| \| \| \| \| \| \| \| \| \| \| \| \| \| \| \| \| \| \| \| \| \| \| --- \| --- \| --- \| --- \| --- \| --- \| --- \| --- \| --- \| --- \| --- \| --- \| --- \| --- \| --- \| --- \| --- \| --- \| --- \| --- \| --- \| --- \| --- \| --- \| \| **Risk analysis excluding patients with outcome prior to the time window** \| \| \| \| \| \| \| \| \| \| \| \| \| \| \| \| \| \| \| \| \| \| \|  \| \| \| Cohort \| \| \| \| Patients in cohort \| \| \| Patients with outcome \| \| \| Risk \| \| \| \| \| \| \| \| \| \| \|  \| \|  \| \| 1 \| DM+AKD+DPP4i but without 3 months before discharge \| \| \| 2,139 \| \| \| 268 \| \| \| 0.125 \| \| \| \| \| \| \| \| \| \| \|  \| \|  \| \| 2 \| DM+AKD without DPP4i but without DPP4i 3 months before discharge \| \| \| 2,122 \| \| \| 335 \| \| \| 0.158 \| \| \| \| \| \| \| \| \| \| \|  \| \| \| \| \| \| \| \| \| \| \| \| \| \| \| \| \| \| \| \| \| \| \| \| \|  \| \|  \| \|  \| \| \| \|  \| \| \| 95% CI \| \| \| z \| \| p \| \| \|  \|  \| \| \| \| \|  \| \|  \| \| **Risk Difference** \| \| \| \| -0.033 \| \| \| (-0.053, -0.012) \| \| \| -3.051 \| \| 0.002 \| \| \|  \|  \| \| \| \| \|  \| \|  \| \| **Risk Ratio** \| \| \| \| 0.794 \| \| \| (0.684, 0.921) \| \| \| N/A \| \| N/A \| \| \|  \|  \| \| \| \| \|  \| \|  \| \| **Odds Ratio** \| \| \| \| 0.764 \| \| \| (0.643, 0.909) \| \| \| N/A \| \| N/A \| \| \|  \|  \| \| \| \| \| **Kaplan - Meier survival analysis excluding patients with outcome prior to the time window** \| \| \| \| \| \| \| \| \| \| \| \| \| \| \| \| \| \| \| \| \| \| \| Cohort \| \| \| \| \| \| Patients in cohort \| \| \| Patients with outcome \| \| \| Median survival (days) \| \| \| Survival probability at end of time window \| \| \| \| \| \| \| 1 \| DM+AKD+DPP4i but without 3 months before discharge \| \| \| \| \| 2,139 \| \| \| 268 \| \| \| -- \| \| \| 77.25% \| \| \| \| \| \| \| 2 \| DM+AKD without DPP4i but without DPP4i 3 months before discharge \| \| \| \| \| 2,122 \| \| \| 335 \| \| \| -- \| \| \| 72.21% \| \| \| \| \| \| \|  \| \| \| \| \| \| \| \| \| \| \| \| \| \| \| \| \| \| \| \| \| \| \| \| \|  \| \| \| \| \| \| χ^2^ \| \| \| df \| \| \| p \| \| \|  \| \|  \|  \| \| \| \| **Log-Rank Test** \| \| \| \| \| \| 10.290 \| \| \| 1 \| \| \| 0.001 \| \| \|  \| \|  \|  \| \| \| \|  \| \| \| \| \| \| \| \| \| \| \| \| \| \| \| \| \| \| \| \| \| \| \| \| \|  \| \| \| \| \| \| Hazard Ratio \| \| \| 95% CI \| \| \| χ^2^ \| \| \| df \| \| p \| \| \| \| \| **Hazard Ratio and Proportionality** \| \| \| \| \| \| 0.769 \| \| \| (0.655, 0.903) \| \| \| 0.862 \| \| \| 1 \| \| 0.353 \| \| \| \| \|  \| \| \| \| \| \| \| \| \| \| \| \| \| \| \| \| \| \| \| \| \| \| \| \| \| **Number of instances excluding patients with outcome prior to the time window** \| \| \| \| \| \| \| \| \| \| \| \| \| \| \| \| \| \| \| \| \| \| \| Cohort \| \| \| \| \| \| Patients in cohort \| \| \| Patients with outcome \| \| \| Mean \| \| \| Standard Deviation \| \| Median \| \| \| \| \| 1 \| DM+AKD+DPP4i but without 3 months before discharge \| \| \| \| \| 2,139 \| \| \| 268 \| \| \| 1.190 \| \| \| 0.455 \| \| 1 \| \| \| \| \| 2 \| DM+AKD without DPP4i but without DPP4i 3 months before discharge \| \| \| \| \| 2,122 \| \| \| 335 \| \| \| 1.182 \| \| \| 0.599 \| \| 1 \| \| \| \| \|  \| \| \| \| \| \| \| \| \| \| \| \| \| \| \| \| \| \| \| \| \| \| \| \| \|  \| \| \| \| \| \| t \| \| \| df \| \| \| p \| \| \|  \| \|  \|  \| \| \| \| **Test Statistics** \| \| \| \| \| \| 0.186 \| \| \| 601 \| \| \| 0.853 \| \| \|  \| \|  \|  \| \| \|  \| **2 MAKE** \| \| \| \| \| \| \| \| \| \| \| \| \| \| \| \| \| --- \| --- \| --- \| --- \| --- \| --- \| --- \| --- \| --- \| --- \| --- \| --- \| --- \| --- \| --- \| --- \| \| **Risk analysis** \| \| \| \| \| \| \| \| \| \| \| \| \| \| \|  \| \| Cohort \| \| \| Patients in cohort \| \| Patients with outcome \| \| Risk \| \| \| \| \| \| \|  \|  \| \| 1 \| DM+AKD+DPP4i but without 3 months before discharge \| \| 2,268 \| \| 304 \| \| 0.134 \| \| \| \| \| \| \|  \|  \| \| 2 \| DM+AKD without DPP4i but without DPP4i 3 months before discharge \| \| 2,268 \| \| 368 \| \| 0.162 \| \| \| \| \| \| \|  \| \| \| \| \| \| \| \| \| \| \| \| \| \| \| \| \|  \|  \| \|  \| \| \|  \| \| 95% CI \| \| z \| p \|  \|  \| \| \| \|  \|  \| \| **Risk Difference** \| \| \| -0.028 \| \| (-0.049, -0.008) \| \| -2.675 \| 0.007 \|  \|  \| \| \| \|  \|  \| \| **Risk Ratio** \| \| \| 0.826 \| \| (0.718, 0.951) \| \| N/A \| N/A \|  \|  \| \| \| \|  \|  \| \| **Odds Ratio** \| \| \| 0.799 \| \| (0.678, 0.942) \| \| N/A \| N/A \|  \|  \| \| \| \| **Kaplan - Meier survival analysis** \| \| \| \| \| \| \| \| \| \| \| \| \| \| \|  \|  \| \| Cohort \| \| \| Patients in cohort \| \| Patients with outcome \| \| Median survival (days) \| Survival probability at end of time window \| \| \| \| \| \|  \|  \| \| 1 \| DM+AKD+DPP4i but without 3 months before discharge \| \| 2,268 \| \| 304 \| \| -- \| 75.95% \| \| \| \| \| \|  \|  \| \| 2 \| DM+AKD without DPP4i but without DPP4i 3 months before discharge \| \| 2,268 \| \| 368 \| \| -- \| 70.72% \| \| \| \| \| \|  \| \| \| \| \| \| \| \| \| \| \| \| \| \| \| \| \|  \|  \| \|  \| \| \| χ^2^ \| \| df \| \| p \|  \|  \|  \| \| \| \|  \|  \| \| **Log-Rank Test** \| \| \| 10.403 \| \| 1 \| \| 0.001 \|  \|  \|  \| \| \| \|  \| \| \| \| \| \| \| \| \| \| \| \| \| \| \| \| \|  \|  \| \|  \| \| \| Hazard Ratio \| \| 95% CI \| \| χ^2^ \| df \| p \| \| \| \| \|  \|  \| \| **Hazard Ratio and Proportionality** \| \| \| 0.779 \| \| (0.669, 0.907) \| \| 0.456 \| 1 \| 0.499 \| \| \| \| \|  \| \| \| \| \| \| \| \| \| \| \| \| \| \| \| \| \| **3 MACE** \| \| \| \| \| \| \| \| \| \| \| \| \| \| \| \| \| **Risk analysis excluding patients with outcome prior to the time window** \| \| \| \| \| \| \| \| \| \| \| \| \| \| \|  \| \| Cohort \| \| \| Patients in cohort \| \| Patients with outcome \| \| Risk \| \| \| \| \| \| \|  \|  \| \| 1 \| DM+AKD+DPP4i but without 3 months before discharge \| \| 1,536 \| \| 273 \| \| 0.178 \| \| \| \| \| \| \|  \|  \| \| 2 \| DM+AKD without DPP4i but without DPP4i 3 months before discharge \| \| 1,539 \| \| 322 \| \| 0.209 \| \| \| \| \| \| \|  \| \| \| \| \| \| \| \| \| \| \| \| \| \| \| \| \|  \|  \| \|  \| \| \|  \| \| 95% CI \| \| z \| p \|  \|  \| \| \| \|  \|  \| \| **Risk Difference** \| \| \| -0.031 \| \| (-0.059, -0.004) \| \| -2.210 \| 0.027 \|  \|  \| \| \| \|  \|  \| \| **Risk Ratio** \| \| \| 0.849 \| \| (0.735, 0.982) \| \| N/A \| N/A \|  \|  \| \| \| \|  \|  \| \| **Odds Ratio** \| \| \| 0.817 \| \| (0.683, 0.978) \| \| N/A \| N/A \|  \|  \| \| \| \|  \| \| \| \| \| \| \| \| \| \| \| \| \| \| \| \| \| **Kaplan - Meier survival analysis excluding patients with outcome prior to the time window** \| \| \| \| \| \| \| \| \| \| \| \| \| \| \|  \|  \| \| Cohort \| \| \| Patients in cohort \| \| Patients with outcome \| \| Median survival (days) \| Survival probability at end of time window \| \| \| \| \| \|  \|  \| \| 1 \| DM+AKD+DPP4i but without 3 months before discharge \| \| 1,536 \| \| 273 \| \| -- \| 69.31% \| \| \| \| \| \|  \|  \| \| 2 \| DM+AKD without DPP4i but without DPP4i 3 months before discharge \| \| 1,539 \| \| 322 \| \| -- \| 64.38% \| \| \| \| \| \|  \| \| \| \| \| \| \| \| \| \| \| \| \| \| \| \| \|  \|  \| \|  \| \| \| χ^2^ \| \| df \| \| p \|  \|  \|  \| \| \| \|  \|  \| \| **Log-Rank Test** \| \| \| 6.082 \| \| 1 \| \| 0.014 \|  \|  \|  \| \| \| \|  \| \| \| \| \| \| \| \| \| \| \| \| \| \| \| \| \|  \|  \| \|  \| \| \| Hazard Ratio \| \| 95% CI \| \| χ^2^ \| df \| p \| \| \| \| \|  \|  \| \| **Hazard Ratio and Proportionality** \| \| \| 0.817 \| \| (0.695, 0.960) \| \| 0.378 \| 1 \| 0.539 \| \| \| \| \|  \| \| \| \| \| \| \| \| \| \| \| \| \| \| \| \| \| **Number of instances excluding patients with outcome prior to the time window** \| \| \| \| \| \| \| \| \| \| \| \| \| \| \|  \|  \| \| Cohort \| \| \| Patients in cohort \| \| Patients with outcome \| \| Mean \| Standard Deviation \| Median \| \| \| \| \|  \|  \| \| 1 \| DM+AKD+DPP4i but without 3 months before discharge \| \| 1,536 \| \| 273 \| \| 2.535 \| 6.087 \| 1 \| \| \| \| \|  \|  \| \| 2 \| DM+AKD without DPP4i but without DPP4i 3 months before discharge \| \| 1,539 \| \| 322 \| \| 2.491 \| 4.425 \| 1 \| \| \| \| \|  \| \| \| \| \| \| \| \| \| \| \| \| \| \| \| \| \|  \|  \| \|  \| \| \| t \| \| df \| \| p \|  \|  \|  \| \| \| \|  \|  \| \| **Test Statistics** \| \| \| 0.102 \| \| 593 \| \| 0.919 \|  \|  \|  \| \| \| \|  \| \| \| \| \| \| \| \| \| \| \| \| \| \| \| \|   **Hypertension (ICD10CM:I10)**   \| **1 Mortality** \| \| \| \| \| \| \| \| \| \| \| \| \| \| \| \| \| \| \| \| \| \| \| \| \| --- \| --- \| --- \| --- \| --- \| --- \| --- \| --- \| --- \| --- \| --- \| --- \| --- \| --- \| --- \| --- \| --- \| --- \| --- \| --- \| --- \| --- \| --- \| --- \| \| **Risk analysis excluding patients with outcome prior to the time window** \| \| \| \| \| \| \| \| \| \| \| \| \| \| \| \| \| \| \| \| \| \| \|  \| \| \| Cohort \| \| \| \| Patients in cohort \| \| \| Patients with outcome \| \| \| Risk \| \| \| \| \| \| \| \| \| \| \|  \| \|  \| \| 1 \| DM+AKD+DPP4i with HTN 1 \| \| \| 4,640 \| \| \| 704 \| \| \| 0.152 \| \| \| \| \| \| \| \| \| \| \|  \| \|  \| \| 2 \| DM+AKD without DPP4i with HTN 1 \| \| \| 4,647 \| \| \| 812 \| \| \| 0.175 \| \| \| \| \| \| \| \| \| \| \|  \| \| \| \| \| \| \| \| \| \| \| \| \| \| \| \| \| \| \| \| \| \| \| \| \|  \| \|  \| \|  \| \| \| \|  \| \| \| 95% CI \| \| \| z \| \| p \| \| \|  \|  \| \| \| \| \|  \| \|  \| \| **Risk Difference** \| \| \| \| -0.023 \| \| \| (-0.038, -0.008) \| \| \| -3.000 \| \| 0.003 \| \| \|  \|  \| \| \| \| \|  \| \|  \| \| **Risk Ratio** \| \| \| \| 0.868 \| \| \| (0.792, 0.952) \| \| \| N/A \| \| N/A \| \| \|  \|  \| \| \| \| \|  \| \|  \| \| **Odds Ratio** \| \| \| \| 0.845 \| \| \| (0.757, 0.943) \| \| \| N/A \| \| N/A \| \| \|  \|  \| \| \| \| \| **Kaplan - Meier survival analysis excluding patients with outcome prior to the time window** \| \| \| \| \| \| \| \| \| \| \| \| \| \| \| \| \| \| \| \| \| \| \| Cohort \| \| \| \| \| \| Patients in cohort \| \| \| Patients with outcome \| \| \| Median survival (days) \| \| \| Survival probability at end of time window \| \| \| \| \| \| \| 1 \| DM+AKD+DPP4i with HTN 1 \| \| \| \| \| 4,640 \| \| \| 704 \| \| \| -- \| \| \| 77.01% \| \| \| \| \| \| \| 2 \| DM+AKD without DPP4i with HTN 1 \| \| \| \| \| 4,647 \| \| \| 812 \| \| \| -- \| \| \| 72.69% \| \| \| \| \| \| \|  \| \| \| \| \| \| \| \| \| \| \| \| \| \| \| \| \| \| \| \| \| \| \| \| \|  \| \| \| \| \| \| χ^2^ \| \| \| df \| \| \| p \| \| \|  \| \|  \|  \| \| \| \| **Log-Rank Test** \| \| \| \| \| \| 15.682 \| \| \| 1 \| \| \| 0.000 \| \| \|  \| \|  \|  \| \| \| \|  \| \| \| \| \| \| \| \| \| \| \| \| \| \| \| \| \| \| \| \| \| \| \| \| \|  \| \| \| \| \| \| Hazard Ratio \| \| \| 95% CI \| \| \| χ^2^ \| \| \| df \| \| p \| \| \| \| \| **Hazard Ratio and Proportionality** \| \| \| \| \| \| 0.816 \| \| \| (0.737, 0.902) \| \| \| 0.056 \| \| \| 1 \| \| 0.813 \| \| \| \| \| **Number of instances excluding patients with outcome prior to the time window** \| \| \| \| \| \| \| \| \| \| \| \| \| \| \| \| \| \| \| \| \| \| \| Cohort \| \| \| \| \| \| Patients in cohort \| \| \| Patients with outcome \| \| \| Mean \| \| \| Standard Deviation \| \| Median \| \| \| \| \| 1 \| DM+AKD+DPP4i with HTN 1 \| \| \| \| \| 4,640 \| \| \| 704 \| \| \| 1.226 \| \| \| 0.524 \| \| 1 \| \| \| \| \| 2 \| DM+AKD without DPP4i with HTN 1 \| \| \| \| \| 4,647 \| \| \| 812 \| \| \| 1.201 \| \| \| 0.428 \| \| 1 \| \| \| \| \|  \| \| \| \| \| \| \| \| \| \| \| \| \| \| \| \| \| \| \| \| \| \| \| \| \|  \| \| \| \| \| \| t \| \| \| df \| \| \| p \| \| \|  \| \|  \|  \| \| \| \| **Test Statistics** \| \| \| \| \| \| 1.027 \| \| \| 1514 \| \| \| 0.305 \| \| \|  \| \|  \|  \| \| \|  \| **2 MAKE** \| \| \| \| \| \| \| \| \| \| \| \| \| \| \| \| \| \| \| \| \| \| \| \| \| \| \| --- \| --- \| --- \| --- \| --- \| --- \| --- \| --- \| --- \| --- \| --- \| --- \| --- \| --- \| --- \| --- \| --- \| --- \| --- \| --- \| --- \| --- \| --- \| --- \| --- \| --- \| \| **Risk analysis** \| \| \| \| \| \| \| \| \| \| \| \| \| \| \| \| \| \| \| \| \| \| \| \| \|  \| \| \| \| \| Cohort \| \| \| \| Patients in cohort \| \| \| Patients with outcome \| \| \| Risk \| \| \| \| \| \| \| \| \| \| \|  \| \| \| \|  \| \| 1 \| DM+AKD+DPP4i with HTN 1 \| \| \| 4,882 \| \| \| 790 \| \| \| 0.162 \| \| \| \| \| \| \| \| \| \| \|  \| \| \| \|  \| \| 2 \| DM+AKD without DPP4i with HTN 1 \| \| \| 4,882 \| \| \| 917 \| \| \| 0.188 \| \| \| \| \| \| \| \| \| \| \|  \| \| \| \| \| \| \| \| \| \| \| \| \| \| \| \| \| \| \| \| \| \| \| \| \| \| \|  \| \| \| \|  \| \|  \| \| \| \|  \| \| \| 95% CI \| \| \| z \| \| p \| \| \|  \|  \| \| \| \| \|  \| \| \| \|  \| \| **Risk Difference** \| \| \| \| -0.026 \| \| \| (-0.041, -0.011) \| \| \| -3.384 \| \| 0.001 \| \| \|  \|  \| \| \| \| \|  \| \| \| \|  \| \| **Risk Ratio** \| \| \| \| 0.862 \| \| \| (0.790, 0.939) \| \| \| N/A \| \| N/A \| \| \|  \|  \| \| \| \| \|  \| \| \| \|  \| \| **Odds Ratio** \| \| \| \| 0.835 \| \| \| (0.752, 0.927) \| \| \| N/A \| \| N/A \| \| \|  \|  \| \| \| \| \| **Kaplan - Meier survival analysis** \| \| \| \| \| \| \| \| \| \| \| \| \| \| \| \| \| \| \| \| \| \| \| \| \| Cohort \| \| \| \| \| \| \| \| Patients in cohort \| \| \| Patients with outcome \| \| \| Median survival (days) \| \| \| Survival probability at end of time window \| \| \| \| \| \| \| 1 \| \| DM+AKD+DPP4i with HTN 1 \| \| \| \| \| \| 4,882 \| \| \| 790 \| \| \| -- \| \| \| 75.49% \| \| \| \| \| \| \| 2 \| \| DM+AKD without DPP4i with HTN 1 \| \| \| \| \| \| 4,882 \| \| \| 917 \| \| \| -- \| \| \| 70.94% \| \| \| \| \| \| \|  \| \| \| \| \| \| \| \| \| \| \| \| \| \| \| \| \| \| \| \| \| \| \| \| \| \| \|  \| \| \| \| \| \| \| \| χ^2^ \| \| \| df \| \| \| p \| \| \|  \| \|  \|  \| \| \| \| **Log-Rank Test** \| \| \| \| \| \| \| \| 19.399 \| \| \| 1 \| \| \| 0.000 \| \| \|  \| \|  \|  \| \| \| \|  \| \| \| \| \| \| \| \| \| \| \| \| \| \| \| \| \| \| \| \| \| \| \| \| \| \| \|  \| \| \| \| \| \| \| \| Hazard Ratio \| \| \| 95% CI \| \| \| χ^2^ \| \| \| df \| \| p \| \| \| \| \| **Hazard Ratio and Proportionality** \| \| \| \| \| \| \| \| 0.808 \| \| \| (0.735, 0.888) \| \| \| 0.689 \| \| \| 1 \| \| 0.406 \| \| \| \| \| **3 MACE** \| \| \| \| \| \| \| \| \| \| \| \| \| \| \| \| \| \| \| \| \| \| \| \| \| \| \|  \| \| \| **Risk analysis excluding patients with outcome prior to the time window** \| \| \| \| \| \| \| \| \| \| \| \| \| \| \| \| \| \| \| \| \| \| \| \|  \|  \| \| \| \| \| Cohort \| \| \| \| Patients in cohort \| \| \| Patients with outcome \| \| \| Risk \| \| \| \| \| \| \| \| \| \| \|  \| \| \| \|  \| \| 1 \| DM+AKD+DPP4i with HTN 1 \| \| \| 3,207 \| \| \| 730 \| \| \| 0.228 \| \| \| \| \| \| \| \| \| \| \|  \| \| \| \|  \| \| 2 \| DM+AKD without DPP4i with HTN 1 \| \| \| 3,254 \| \| \| 775 \| \| \| 0.238 \| \| \| \| \| \| \| \| \| \| \|  \| \| \| \| \| \| \| \| \| \| \| \| \| \| \| \| \| \| \| \| \| \| \| \| \| \| \|  \| \| \| \|  \| \|  \| \| \| \|  \| \| \| 95% CI \| \| \| z \| \| p \| \| \|  \|  \| \| \| \| \|  \| \| \| \|  \| \| **Risk Difference** \| \| \| \| -0.011 \| \| \| (-0.031, 0.010) \| \| \| -1.002 \| \| 0.316 \| \| \|  \|  \| \| \| \| \|  \| \| \| \|  \| \| **Risk Ratio** \| \| \| \| 0.956 \| \| \| (0.875, 1.044) \| \| \| N/A \| \| N/A \| \| \|  \|  \| \| \| \| \|  \| \| \| \|  \| \| **Odds Ratio** \| \| \| \| 0.943 \| \| \| (0.840, 1.058) \| \| \| N/A \| \| N/A \| \| \|  \|  \| \| \| \| \|  \| \| \| \| \| \| \| \| \| \| \| \| \| \| \| \| \| \| \| \| \| \| \| \| \| \| \|  \| \| \| **Kaplan - Meier survival analysis excluding patients with outcome prior to the time window** \| \| \| \| \| \| \| \| \| \| \| \| \| \| \| \| \| \| \| \| \| \| \| \|  \| \| \| \|  \| \| Cohort \| \| \| \| Patients in cohort \| \| \| Patients with outcome \| \| \| Median survival (days) \| \| Survival probability at end of time window \| \| \| \| \| \| \| \| \|  \| \| \| \|  \| \| 1 \| DM+AKD+DPP4i with HTN 1 \| \| \| 3,207 \| \| \| 730 \| \| \| -- \| \| 66.97% \| \| \| \| \| \| \| \| \|  \| \| \| \|  \| \| 2 \| DM+AKD without DPP4i with HTN 1 \| \| \| 3,254 \| \| \| 775 \| \| \| -- \| \| 64.63% \| \| \| \| \| \| \| \| \|  \| \| \| \| \| \| \| \| \| \| \| \| \| \| \| \| \| \| \| \| \| \| \| \| \| \| \|  \| \| \| \|  \| \|  \| \| \| \| χ^2^ \| \| \| df \| \| \| p \| \|  \| \| \|  \|  \| \| \| \| \|  \| \| \| \|  \| \| **Log-Rank Test** \| \| \| \| 4.520 \| \| \| 1 \| \| \| 0.034 \| \|  \| \| \|  \|  \| \| \| \| \|  \| \| \| \| \| \| \| \| \| \| \| \| \| \| \| \| \| \| \| \| \| \| \| \| \| \| \|  \| \| \| \|  \| \|  \| \| \| \| Hazard Ratio \| \| \| 95% CI \| \| \| χ^2^ \| \| df \| \| \| p \| \| \| \| \| \|  \| \| \| \|  \| \| **Hazard Ratio and Proportionality** \| \| \| \| 0.896 \| \| \| (0.810, 0.992) \| \| \| 1.784 \| \| 1 \| \| \| 0.182 \| \| \| \| \| \|  \| \| \| \| \| \| \| \| \| \| \| \| \| \| \| \| \| \| \| \| \| \| \| \| \| \| \|  \| \| \| **Number of instances excluding patients with outcome prior to the time window** \| \| \| \| \| \| \| \| \| \| \| \| \| \| \| \| \| \| \| \| \| \| \| \|  \| \| \| \|  \| \| Cohort \| \| \| \| Patients in cohort \| \| \| Patients with outcome \| \| \| Mean \| \| Standard Deviation \| \| \| Median \| \| \| \| \| \|  \| \| \| \|  \| \| 1 \| DM+AKD+DPP4i with HTN 1 \| \| \| 3,207 \| \| \| 730 \| \| \| 3.908 \| \| 8.602 \| \| \| 1 \| \| \| \| \| \|  \| \| \| \|  \| \| 2 \| DM+AKD without DPP4i with HTN 1 \| \| \| 3,254 \| \| \| 775 \| \| \| 2.823 \| \| 5.977 \| \| \| 1 \| \| \| \| \| \|  \| \| \| \| \| \| \| \| \| \| \| \| \| \| \| \| \| \| \| \| \| \| \| \| \| \| \|  \| \| \| \|  \| \|  \| \| \| \| t \| \| \| df \| \| \| p \| \|  \| \| \|  \|  \| \| \| \| \|  \| \| \| \|  \| \| **Test Statistics** \| \| \| \| 2.855 \| \| \| 1503 \| \| \| 0.004 \| \|  \| \| \|  \|  \| \| \| \| \|  \| \| \| \| \| \| \| \| \| \| \| \| \| \| \| \| \| \| \| \| \| \| \| \| \| \|   **Without hypertension**   \| **1 Mortality** \| \| \| \| \| \| \| \| \| \| \| \| \| \| \| \| \| \| \| \| --- \| --- \| --- \| --- \| --- \| --- \| --- \| --- \| --- \| --- \| --- \| --- \| --- \| --- \| --- \| --- \| --- \| --- \| --- \| \| **Risk analysis excluding patients with outcome prior to the time window** \| \| \| \| \| \| \| \| \| \| \| \| \| \| \| \| \| \|  \| \| \| \| \| Cohort \| \| \| Patients in cohort \| \| Patients with outcome \| \| Risk \| \| \| \| \| \| \|  \| \| \|  \| \| \| 1 \| DM+AKD+DPP4i without HTN 1 \| \| 1,878 \| \| 248 \| \| 0.132 \| \| \| \| \| \| \|  \| \| \|  \| \| \| 2 \| DM+AKD without DPP4i without HTN 1 \| \| 1,822 \| \| 291 \| \| 0.160 \| \| \| \| \| \| \|  \| \| \| \| \| \| \| \| \| \| \| \| \| \| \| \| \| \| \| \|  \| \| \|  \| \| \|  \| \| \|  \| \| 95% CI \| \| z \| p \|  \|  \| \| \| \|  \| \| \|  \| \| \| **Risk Difference** \| \| \| -0.028 \| \| (-0.050, -0.005) \| \| -2.384 \| 0.017 \|  \|  \| \| \| \|  \| \| \|  \| \| \| **Risk Ratio** \| \| \| 0.827 \| \| (0.707, 0.967) \| \| N/A \| N/A \|  \|  \| \| \| \|  \| \| \|  \| \| \| **Odds Ratio** \| \| \| 0.800 \| \| (0.666, 0.961) \| \| N/A \| N/A \|  \|  \| \| \| \|  \| \| \| \|  \| \| \| \| \| \| \| \| \| \| \| \| \| \| **Kaplan - Meier survival analysis excluding patients with outcome prior to the time window** \| \| \| \| \| \| \| \| \| \| \| \| \| \| \| \| \| \|  \| \| \|  \| \| \| Cohort \| \| \| Patients in cohort \| \| Patients with outcome \| \| Median survival (days) \| Survival probability at end of time window \| \| \| \| \| \|  \| \| \|  \| \| \| 1 \| DM+AKD+DPP4i without HTN 1 \| \| 1,878 \| \| 248 \| \| -- \| 72.25% \| \| \| \| \| \|  \| \| \|  \| \| \| 2 \| DM+AKD without DPP4i without HTN 1 \| \| 1,822 \| \| 291 \| \| -- \| 66.27% \| \| \| \| \| \|  \| \| \| \| \| \| \| \| \| \| \| \| \| \| \| \| \| \| \| \|  \| \| \|  \| \| \|  \| \| \| χ^2^ \| \| df \| \| p \|  \|  \|  \| \| \| \|  \| \| \|  \| \| \| **Log-Rank Test** \| \| \| 12.197 \| \| 1 \| \| 0.000 \|  \|  \|  \| \| \| \|  \| \| \| \| \| \| \| \| \| \| \| \| \| \| \| \| \| \| \| \|  \| \| \|  \| \| \|  \| \| \| Hazard Ratio \| \| 95% CI \| \| χ^2^ \| df \| p \| \| \| \| \|  \| \| \|  \| \| \| **Hazard Ratio and Proportionality** \| \| \| 0.740 \| \| (0.625, 0.877) \| \| 2.907 \| 1 \| 0.088 \| \| \| \| \|  \| \| \| \| \| \| \| \| \| \| \| \| \| \| \| \| \| \| \| \| **Number of instances excluding patients with outcome prior to the time window** \| \| \| \| \| \| \| \| \| \| \| \| \| \| \| \| \| \|  \| \| \|  \| \| \| Cohort \| \| \| Patients in cohort \| \| Patients with outcome \| \| Mean \| Standard Deviation \| Median \| \| \| \| \|  \| \| \|  \| \| \| 1 \| DM+AKD+DPP4i without HTN 1 \| \| 1,878 \| \| 248 \| \| 1.173 \| 0.466 \| 1 \| \| \| \| \|  \| \| \|  \| \| \| 2 \| DM+AKD without DPP4i without HTN 1 \| \| 1,822 \| \| 291 \| \| 1.182 \| 0.413 \| 1 \| \| \| \| \|  \| \| \| \| \| \| \| \| \| \| \| \| \| \| \| \| \| \| \| \|  \| \| \|  \| \| \|  \| \| \| t \| \| df \| \| p \|  \|  \|  \| \| \| \|  \| \| \|  \| \| \| **Test Statistics** \| \| \| -0.231 \| \| 537 \| \| 0.817 \|  \|  \|  \| \| \| \|  \| \| \| \| \| \| \| \| \| \| \| \| \| \| \| \| \| \| \| \| **2 MAKE** \| \| \| \| \| \| \| \| \| \| \| \| \| \| \| \| \| \| \| \| **Risk analysis** \| \| \| \| \| \| \| \| \| \| \| \| \| \| \| \| \| \|  \| \| \| \| \| Cohort \| \| \| Patients in cohort \| \| Patients with outcome \| \| Risk \| \| \| \| \| \| \|  \| \| \|  \| \| \| 1 \| DM+AKD+DPP4i without HTN 1 \| \| 1,971 \| \| 277 \| \| 0.141 \| \| \| \| \| \| \|  \| \| \|  \| \| \| 2 \| DM+AKD without DPP4i without HTN 1 \| \| 1,971 \| \| 323 \| \| 0.164 \| \| \| \| \| \| \|  \| \| \| \| \| \| \| \| \| \| \| \| \| \| \| \| \| \| \| \|  \| \| \|  \| \| \|  \| \| \|  \| \| 95% CI \| \| z \| p \|  \|  \| \| \| \|  \| \| \|  \| \| \| **Risk Difference** \| \| \| -0.023 \| \| (-0.046, -0.001) \| \| -2.040 \| 0.041 \|  \|  \| \| \| \|  \| \| \|  \| \| \| **Risk Ratio** \| \| \| 0.858 \| \| (0.740, 0.994) \| \| N/A \| N/A \|  \|  \| \| \| \|  \| \| \|  \| \| \| **Odds Ratio** \| \| \| 0.834 \| \| (0.701, 0.993) \| \| N/A \| N/A \|  \|  \| \| \| \| **Kaplan - Meier survival analysis** \| \| \| \| \| \| \| \| \| \| \| \| \| \| \| \| \| \|  \| \| \|  \| \| \| Cohort \| \| \| Patients in cohort \| \| Patients with outcome \| \| Median survival (days) \| Survival probability at end of time window \| \| \| \| \| \|  \| \| \|  \| \| \| 1 \| DM+AKD+DPP4i without HTN 1 \| \| 1,971 \| \| 277 \| \| -- \| 70.49% \| \| \| \| \| \|  \| \| \|  \| \| \| 2 \| DM+AKD without DPP4i without HTN 1 \| \| 1,971 \| \| 323 \| \| -- \| 63.77% \| \| \| \| \| \|  \| \| \| \| \| \| \| \| \| \| \| \| \| \| \| \| \| \| \| \|  \| \| \|  \| \| \|  \| \| \| χ^2^ \| \| df \| \| p \|  \|  \|  \| \| \| \|  \| \| \|  \| \| \| **Log-Rank Test** \| \| \| 14.126 \| \| 1 \| \| 0.000 \|  \|  \|  \| \| \| \|  \| \| \| \| \| \| \| \| \| \| \| \| \| \| \| \| \| \| \| \|  \| \| \|  \| \| \|  \| \| \| Hazard Ratio \| \| 95% CI \| \| χ^2^ \| df \| p \| \| \| \| \|  \| \| \|  \| \| \| **Hazard Ratio and Proportionality** \| \| \| 0.736 \| \| (0.627, 0.864) \| \| 2.472 \| 1 \| 0.116 \| \| \| \| \| **3 MACE** \| \| \| \| \| \| \| \| \| \| \| \| \| \| \| \| \| \| \| \|  \| \| **Risk analysis excluding patients with outcome prior to the time window** \| \| \| \| \| \| \| \| \| \| \| \| \| \| \| \| \| \|  \|  \| \| \| \| \| Cohort \| \| \| Patients in cohort \| \| Patients with outcome \| \| Risk \| \| \| \| \| \| \|  \| \| \|  \| \| \| 1 \| DM+AKD+DPP4i without HTN 1 \| \| 1,469 \| \| 228 \| \| 0.155 \| \| \| \| \| \| \|  \| \| \|  \| \| \| 2 \| DM+AKD without DPP4i without HTN 1 \| \| 1,430 \| \| 259 \| \| 0.181 \| \| \| \| \| \| \|  \| \| \| \| \| \| \| \| \| \| \| \| \| \| \| \| \| \| \| \|  \| \| \|  \| \| \|  \| \| \|  \| \| 95% CI \| \| z \| p \|  \|  \| \| \| \|  \| \| \|  \| \| \| **Risk Difference** \| \| \| -0.026 \| \| (-0.053, 0.001) \| \| -1.866 \| 0.062 \|  \|  \| \| \| \|  \| \| \|  \| \| \| **Risk Ratio** \| \| \| 0.857 \| \| (0.728, 1.008) \| \| N/A \| N/A \|  \|  \| \| \| \|  \| \| \|  \| \| \| **Odds Ratio** \| \| \| 0.831 \| \| (0.683, 1.010) \| \| N/A \| N/A \|  \|  \| \| \| \|  \| \| **Kaplan - Meier survival analysis excluding patients with outcome prior to the time window** \| \| \| \| \| \| \| \| \| \| \| \| \| \| \| \| \| \|  \| \| \|  \| \| \| Cohort \| \| \| Patients in cohort \| \| Patients with outcome \| \| Median survival (days) \| Survival probability at end of time window \| \| \| \| \| \|  \| \| \|  \| \| \| 1 \| DM+AKD+DPP4i without HTN 1 \| \| 1,469 \| \| 228 \| \| -- \| 68.85% \| \| \| \| \| \|  \| \| \|  \| \| \| 2 \| DM+AKD without DPP4i without HTN 1 \| \| 1,430 \| \| 259 \| \| -- \| 63.70% \| \| \| \| \| \|  \| \| \| \| \| \| \| \| \| \| \| \| \| \| \| \| \| \| \| \|  \| \| \|  \| \| \|  \| \| \| χ^2^ \| \| df \| \| p \|  \|  \|  \| \| \| \|  \| \| \|  \| \| \| **Log-Rank Test** \| \| \| 8.090 \| \| 1 \| \| 0.004 \|  \|  \|  \| \| \| \|  \| \| \| \| \| \| \| \| \| \| \| \| \| \| \| \| \| \| \| \|  \| \| \|  \| \| \|  \| \| \| Hazard Ratio \| \| 95% CI \| \| χ^2^ \| df \| p \| \| \| \| \|  \| \| \|  \| \| \| **Hazard Ratio and Proportionality** \| \| \| 0.773 \| \| (0.647, 0.923) \| \| 2.501 \| 1 \| 0.114 \| \| \| \| \|  \| \| \| \| \| \| \| \| \| \| \| \| \| \| \| \| \| \| \| \|  \| \| **Number of instances excluding patients with outcome prior to the time window** \| \| \| \| \| \| \| \| \| \| \| \| \| \| \| \| \| \|  \| \| \|  \| \| \| Cohort \| \| \| Patients in cohort \| \| Patients with outcome \| \| Mean \| Standard Deviation \| Median \| \| \| \| \|  \| \| \|  \| \| \| 1 \| DM+AKD+DPP4i without HTN 1 \| \| 1,469 \| \| 228 \| \| 1.697 \| 2.756 \| 1 \| \| \| \| \|  \| \| \|  \| \| \| 2 \| DM+AKD without DPP4i without HTN 1 \| \| 1,430 \| \| 259 \| \| 1.703 \| 2.415 \| 1 \| \| \| \| \|  \| \| \| \| \| \| \| \| \| \| \| \| \| \| \| \| \| \| \| \|  \| \| \|  \| \| \|  \| \| \| t \| \| df \| \| p \|  \|  \|  \| \| \| \|  \| \| \|  \| \| \| **Test Statistics** \| \| \| -0.023 \| \| 485 \| \| 0.982 \|  \|  \|  \| \| \|   **Heart failure (ICD10CM:I50)**   \| **1 Mortality** \| \| \| \| \| \| \| \| \| \| \| \| \| \| --- \| --- \| --- \| --- \| --- \| --- \| --- \| --- \| --- \| --- \| --- \| --- \| --- \| \|  \| \| **Risk analysis excluding patients with outcome prior to the time window** \| \| \| \| \| \| \| \| \| \| \| \|  \|  \| \| \| Cohort \| \| \| Patients in cohort \| Patients with outcome \| Risk \| \| \| \| \|  \| \| \|  \| 1 \| \| DM+AKD+DPP4i heart failure 1 \| 1,541 \| 331 \| 0.215 \| \| \| \| \|  \| \| \|  \| 2 \| \| DM+AKD without DPP4i with heart failure 1 \| 1,549 \| 382 \| 0.247 \| \| \| \| \|  \| \| \| \| \| \| \| \| \| \| \| \| \| \|  \| \| \|  \|  \| \| \|  \| 95% CI \| z \| p \|  \|  \| \|  \| \| \|  \| **Risk Difference** \| \| \| -0.032 \| (-0.062, -0.002) \| -2.099 \| 0.036 \|  \|  \| \|  \| \| \|  \| **Risk Ratio** \| \| \| 0.871 \| (0.765, 0.991) \| N/A \| N/A \|  \|  \| \|  \| \| \|  \| **Odds Ratio** \| \| \| 0.836 \| (0.707, 0.988) \| N/A \| N/A \|  \|  \| \|  \| \| \| \| \| \| \| \| \| \| \| \| \| \|  \| \| **Kaplan - Meier survival analysis excluding patients with outcome prior to the time window** \| \| \| \| \| \| \| \| \| \| \| \|  \| \| \|  \| Cohort \| \| \| Patients in cohort \| Patients with outcome \| Median survival (days) \| Survival probability at end of time window \| \| \| \|  \| \| \|  \| 1 \| \| DM+AKD+DPP4i heart failure 1 \| 1,541 \| 331 \| -- \| 67.34% \| \| \| \|  \| \| \|  \| 2 \| \| DM+AKD without DPP4i with heart failure 1 \| 1,549 \| 382 \| -- \| 60.75% \| \| \| \|  \| \| \| \| \| \| \| \| \| \| \| \| \| \|  \| \| \|  \|  \| \| \| χ^2^ \| df \| p \|  \|  \|  \| \|  \| \| \|  \| **Log-Rank Test** \| \| \| 12.585 \| 1 \| 0.000 \|  \|  \|  \| \|  \| \| \| \| \| \| \| \| \| \| \| \| \| \|  \| \| \|  \|  \| \| \| Hazard Ratio \| 95% CI \| χ^2^ \| df \| p \| \| \|  \| \| \|  \| **Hazard Ratio and Proportionality** \| \| \| 0.767 \| (0.662, 0.888) \| 1.657 \| 1 \| 0.198 \| \| \|  \| \| \| \| \| \| \| \| \| \| \| \| \| \|  \| \| **Number of instances excluding patients with outcome prior to the time window** \| \| \| \| \| \| \| \| \| \| \| \|  \| \| \|  \| Cohort \| \| \| Patients in cohort \| Patients with outcome \| Mean \| Standard Deviation \| Median \| \| \|  \| \| \|  \| 1 \| \| DM+AKD+DPP4i heart failure 1 \| 1,541 \| 331 \| 1.263 \| 0.628 \| 1 \| \| \|  \| \| \|  \| 2 \| \| DM+AKD without DPP4i with heart failure 1 \| 1,549 \| 382 \| 1.196 \| 0.442 \| 1 \| \| \|  \| \| \| \| \| \| \| \| \| \| \| \| \| \|  \| \| \|  \|  \| \| \| t \| df \| p \|  \|  \|  \| \|  \| \| \|  \| **Test Statistics** \| \| \| 1.652 \| 711 \| 0.099 \|  \|  \|  \| \|  \| \| \| \| \| \| \| \| \| \| \| \| \| \| **2 MAKE** \| \| \| \| \| \| \| \| \| \| \| \| \| \|  \| \| **Risk analysis** \| \| \| \| \| \| \| \| \| \| \| \|  \|  \| \| \| Cohort \| \| \| Patients in cohort \| Patients with outcome \| Risk \| \| \| \| \|  \| \| \|  \| 1 \| \| DM+AKD+DPP4i heart failure 1 \| 1,623 \| 371 \| 0.229 \| \| \| \| \|  \| \| \|  \| 2 \| \| DM+AKD without DPP4i with heart failure 1 \| 1,623 \| 421 \| 0.259 \| \| \| \| \|  \| \| \| \| \| \| \| \| \| \| \| \| \| \|  \| \| \|  \|  \| \| \|  \| 95% CI \| z \| p \|  \|  \| \|  \| \| \|  \| **Risk Difference** \| \| \| -0.031 \| (-0.060, -0.001) \| -2.043 \| 0.041 \|  \|  \| \|  \| \| \|  \| **Risk Ratio** \| \| \| 0.881 \| (0.780, 0.995) \| N/A \| N/A \|  \|  \| \|  \| \| \|  \| **Odds Ratio** \| \| \| 0.846 \| (0.721, 0.993) \| N/A \| N/A \|  \|  \| \|  \| \| **Kaplan - Meier survival analysis** \| \| \| \| \| \| \| \| \| \| \| \|  \| \| \|  \| Cohort \| \| \| Patients in cohort \| Patients with outcome \| Median survival (days) \| Survival probability at end of time window \| \| \| \|  \| \| \|  \| 1 \| \| DM+AKD+DPP4i heart failure 1 \| 1,623 \| 371 \| -- \| 65.15% \| \| \| \|  \| \| \|  \| 2 \| \| DM+AKD without DPP4i with heart failure 1 \| 1,623 \| 421 \| -- \| 58.74% \| \| \| \|  \| \| \| \| \| \| \| \| \| \| \| \| \| \|  \| \| \|  \|  \| \| \| χ^2^ \| df \| p \|  \|  \|  \| \|  \| \| \|  \| **Log-Rank Test** \| \| \| 12.831 \| 1 \| 0.000 \|  \|  \|  \| \|  \| \| \| \| \| \| \| \| \| \| \| \| \| \|  \| \| \|  \|  \| \| \| Hazard Ratio \| 95% CI \| χ^2^ \| df \| p \| \| \|  \| \| \|  \| **Hazard Ratio and Proportionality** \| \| \| 0.775 \| (0.674, 0.891) \| 2.001 \| 1 \| 0.157 \| \| \| **3 MACE** \| \| \| \| \| \| \| \| \| \| \| \| \| \|  \| \| **Risk analysis excluding patients with outcome prior to the time window** \| \| \| \| \| \| \| \| \| \| \| \|  \|  \| \| \| Cohort \| \| \| Patients in cohort \| Patients with outcome \| Risk \| \| \| \| \|  \| \| \|  \| 1 \| \| DM+AKD+DPP4i heart failure 1 \| 915 \| 304 \| 0.332 \| \| \| \| \|  \| \| \|  \| 2 \| \| DM+AKD without DPP4i with heart failure 1 \| 910 \| 298 \| 0.327 \| \| \| \| \|  \| \| \| \| \| \| \| \| \| \| \| \| \| \|  \| \| \|  \|  \| \| \|  \| 95% CI \| z \| p \|  \|  \| \|  \| \| \|  \| **Risk Difference** \| \| \| 0.005 \| (-0.038, 0.048) \| 0.217 \| 0.829 \|  \|  \| \|  \| \| \|  \| **Risk Ratio** \| \| \| 1.015 \| (0.890, 1.156) \| N/A \| N/A \|  \|  \| \|  \| \| \|  \| **Odds Ratio** \| \| \| 1.022 \| (0.841, 1.242) \| N/A \| N/A \|  \|  \| \|  \| \| \| \| \| \| \| \| \| \| \| \| \| \|  \| \|  \| \| \|  \| \| \| \| \| \| \| \| \|  \| \|  \| \| \| 708 patients in Cohort 1 and 713 patients in Cohort 2 were excluded from results because they had the outcome prior to the time window. \| \| \| \| \| \| \| \| \|  \| \| **Kaplan - Meier survival analysis excluding patients with outcome prior to the time window** \| \| \| \| \| \| \| \| \| \| \| \|  \| \| \|  \| Cohort \| \| \| Patients in cohort \| Patients with outcome \| Median survival (days) \| Survival probability at end of time window \| \| \| \|  \| \| \|  \| 1 \| \| DM+AKD+DPP4i heart failure 1 \| 915 \| 304 \| -- \| 51.99% \| \| \| \|  \| \| \|  \| 2 \| \| DM+AKD without DPP4i with heart failure 1 \| 910 \| 298 \| -- \| 50.24% \| \| \| \|  \| \| \| \| \| \| \| \| \| \| \| \| \| \|  \| \| \|  \|  \| \| \| χ^2^ \| df \| p \|  \|  \|  \| \|  \| \| \|  \| **Log-Rank Test** \| \| \| 2.195 \| 1 \| 0.138 \|  \|  \|  \| \|  \| \| \| \| \| \| \| \| \| \| \| \| \| \|  \| \| \|  \|  \| \| \| Hazard Ratio \| 95% CI \| χ^2^ \| df \| p \| \| \|  \| \| \|  \| **Hazard Ratio and Proportionality** \| \| \| 0.886 \| (0.755, 1.040) \| 3.760 \| 1 \| 0.052 \| \| \|  \| \| \| \| \| \| \| \| \| \| \| \| \| \|  \| \| **Number of instances excluding patients with outcome prior to the time window** \| \| \| \| \| \| \| \| \| \| \| \|  \| \| \|  \| Cohort \| \| \| Patients in cohort \| Patients with outcome \| Mean \| Standard Deviation \| Median \| \| \|  \| \| \|  \| 1 \| \| DM+AKD+DPP4i heart failure 1 \| 915 \| 304 \| 3.316 \| 7.061 \| 1 \| \| \|  \| \| \|  \| 2 \| \| DM+AKD without DPP4i with heart failure 1 \| 910 \| 298 \| 3.054 \| 10.209 \| 1 \| \| \|  \| \| \| \| \| \| \| \| \| \| \| \| \| \|  \| \| \|  \|  \| \| \| t \| df \| p \|  \|  \|  \| \|  \| \| \|  \| **Test Statistics** \| \| \| 0.367 \| 600 \| 0.714 \|  \|  \|  \|   **Without heart failure**   \| **1 Mortality** \| \| \| \| \| \| \| \| \| \| \| \| \| --- \| --- \| --- \| --- \| --- \| --- \| --- \| --- \| --- \| --- \| --- \| --- \| \|  \| \| **Risk analysis excluding patients with outcome prior to the time window** \| \| \| \| \| \| \| \| \| \| \|  \|  \| \| \| Cohort \| \| Patients in cohort \| Patients with outcome \| Risk \| \| \| \| \|  \| \| \|  \| 1 \| DM+AKD+DPP4i without heart failure \| 5,532 \| 716 \| 0.129 \| \| \| \| \|  \| \| \|  \| 2 \| DM+AKD without DPP4i without heart failure \| 5,458 \| 817 \| 0.150 \| \| \| \| \|  \| \| \| \| \| \| \| \| \| \| \| \| \|  \| \| \|  \|  \| \|  \| 95% CI \| z \| p \|  \|  \| \|  \| \| \|  \| **Risk Difference** \| \| -0.020 \| (-0.033, -0.007) \| -3.065 \| 0.002 \|  \|  \| \|  \| \| \|  \| **Risk Ratio** \| \| 0.865 \| (0.788, 0.949) \| N/A \| N/A \|  \|  \| \|  \| \| \|  \| **Odds Ratio** \| \| 0.845 \| (0.758, 0.941) \| N/A \| N/A \|  \|  \| \|  \| \| \| \| \| \| \| \| \| \| \| \| \|  \| \| \|  \| Cohort \| \| Patients in cohort \| Patients with outcome \| Median survival (days) \| Survival probability at end of time window \| \| \| \|  \| \| \|  \| 1 \| DM+AKD+DPP4i without heart failure \| 5,532 \| 716 \| -- \| 77.59% \| \| \| \|  \| \| \|  \| 2 \| DM+AKD without DPP4i without heart failure \| 5,458 \| 817 \| -- \| 73.69% \| \| \| \|  \| \| \| \| \| \| \| \| \| \| \| \| \|  \| \| \|  \|  \| \| χ^2^ \| df \| p \|  \|  \|  \| \|  \| \| \|  \| **Log-Rank Test** \| \| 23.008 \| 1 \| 0.000 \|  \|  \|  \| \|  \| \| \| \| \| \| \| \| \| \| \| \| \|  \| \| \|  \|  \| \| Hazard Ratio \| 95% CI \| χ^2^ \| df \| p \| \| \|  \| \| \|  \| **Hazard Ratio and Proportionality** \| \| 0.783 \| (0.708, 0.865) \| 8.522 \| 1 \| 0.004 \| \| \|  \| \| \| \| \| \| \| \| \| \| \| \| \|  \| \| **Number of instances excluding patients with outcome prior to the time window** \| \| \| \| \| \| \| \| \| \| \|  \| \| \|  \| Cohort \| \| Patients in cohort \| Patients with outcome \| Mean \| Standard Deviation \| Median \| \| \|  \| \| \|  \| 1 \| DM+AKD+DPP4i without heart failure \| 5,532 \| 716 \| 1.200 \| 0.548 \| 1 \| \| \|  \| \| \|  \| 2 \| DM+AKD without DPP4i without heart failure \| 5,458 \| 817 \| 1.196 \| 0.465 \| 1 \| \| \|  \| \| \| \| \| \| \| \| \| \| \| \| \|  \| \| \|  \|  \| \| t \| df \| p \|  \|  \|  \| \|  \| \| \|  \| **Test Statistics** \| \| 0.150 \| 1531 \| 0.881 \|  \|  \|  \| \|  \| \| \| \| \| \| \| \| \| \| \| \| \| **2 MAKE** \| \| \| \| \| \| \| \| \| \| \| \| \|  \| \| **Risk analysis** \| \| \| \| \| \| \| \| \| \| \|  \|  \| \| \| Cohort \| \| Patients in cohort \| Patients with outcome \| Risk \| \| \| \| \|  \| \| \|  \| 1 \| DM+AKD+DPP4i without heart failure \| 5,822 \| 793 \| 0.136 \| \| \| \| \|  \| \| \|  \| 2 \| DM+AKD without DPP4i without heart failure \| 5,822 \| 910 \| 0.156 \| \| \| \| \|  \| \| \| \| \| \| \| \| \| \| \| \| \|  \| \| \|  \|  \| \|  \| 95% CI \| z \| p \|  \|  \| \|  \| \| \|  \| **Risk Difference** \| \| -0.020 \| (-0.033, -0.007) \| -3.068 \| 0.002 \|  \|  \| \|  \| \| \|  \| **Risk Ratio** \| \| 0.871 \| (0.798, 0.952) \| N/A \| N/A \|  \|  \| \|  \| \| \|  \| **Odds Ratio** \| \| 0.851 \| (0.768, 0.944) \| N/A \| N/A \|  \|  \| \|  \| \| \| \| \| \| \| \| \| \| \| \| \|  \| \| **Kaplan - Meier survival analysis** \| \| \| \| \| \| \| \| \| \| \|  \| \| \|  \| Cohort \| \| Patients in cohort \| Patients with outcome \| Median survival (days) \| Survival probability at end of time window \| \| \| \|  \| \| \|  \| 1 \| DM+AKD+DPP4i without heart failure \| 5,822 \| 793 \| -- \| 76.60% \| \| \| \|  \| \| \|  \| 2 \| DM+AKD without DPP4i without heart failure \| 5,822 \| 910 \| -- \| 71.77% \| \| \| \|  \| \| \| \| \| \| \| \| \| \| \| \| \|  \| \| \|  \|  \| \| χ^2^ \| df \| p \|  \|  \|  \| \|  \| \| \|  \| **Log-Rank Test** \| \| 29.093 \| 1 \| 0.000 \|  \|  \|  \| \|  \| \| \| \| \| \| \| \| \| \| \| \| \|  \| \| \|  \|  \| \| Hazard Ratio \| 95% CI \| χ^2^ \| df \| p \| \| \|  \| \| \|  \| **Hazard Ratio and Proportionality** \| \| 0.770 \| (0.700, 0.847) \| 5.020 \| 1 \| 0.025 \| \| \|  \| \| \| \| \| \| \| \| \| \| \| \| \| **3 MACE** \| \| \| \| \| \| \| \| \| \| \| \| \|  \| \| **Risk analysis excluding patients with outcome prior to the time window** \| \| \| \| \| \| \| \| \| \| \|  \|  \| \| \| Cohort \| \| Patients in cohort \| Patients with outcome \| Risk \| \| \| \| \|  \| \| \|  \| 1 \| DM+AKD+DPP4i without heart failure \| 4,129 \| 699 \| 0.169 \| \| \| \| \|  \| \| \|  \| 2 \| DM+AKD without DPP4i without heart failure \| 4,273 \| 780 \| 0.183 \| \| \| \| \|  \| \| \| \| \| \| \| \| \| \| \| \| \|  \| \| \|  \|  \| \|  \| 95% CI \| z \| p \|  \|  \| \|  \| \| \|  \| **Risk Difference** \| \| -0.013 \| (-0.030, 0.003) \| -1.594 \| 0.111 \|  \|  \| \|  \| \| \|  \| **Risk Ratio** \| \| 0.927 \| (0.845, 1.017) \| N/A \| N/A \|  \|  \| \|  \| \| \|  \| **Odds Ratio** \| \| 0.913 \| (0.816, 1.021) \| N/A \| N/A \|  \|  \| \|  \| \| \| \| \| \| \| \| \| \| \| \| \|  \| \| **Kaplan - Meier survival analysis excluding patients with outcome prior to the time window** \| \| \| \| \| \| \| \| \| \| \|  \| \| \|  \| Cohort \| \| Patients in cohort \| Patients with outcome \| Median survival (days) \| Survival probability at end of time window \| \| \| \|  \| \| \|  \| 1 \| DM+AKD+DPP4i without heart failure \| 4,129 \| 699 \| -- \| 71.83% \| \| \| \|  \| \| \|  \| 2 \| DM+AKD without DPP4i without heart failure \| 4,273 \| 780 \| -- \| 68.85% \| \| \| \|  \| \| \| \| \| \| \| \| \| \| \| \| \|  \| \| \|  \|  \| \| χ^2^ \| df \| p \|  \|  \|  \| \|  \| \| \|  \| **Log-Rank Test** \| \| 11.692 \| 1 \| 0.001 \|  \|  \|  \| \|  \| \| \| \| \| \| \| \| \| \| \| \| \|  \| \| \|  \|  \| \| Hazard Ratio \| 95% CI \| χ^2^ \| df \| p \| \| \|  \| \| \|  \| **Hazard Ratio and Proportionality** \| \| 0.837 \| (0.756, 0.927) \| 6.980 \| 1 \| 0.008 \| \| \|  \| \| \| \| \| \| \| \| \| \| \| \| \|  \| \| **Number of instances excluding patients with outcome prior to the time window** \| \| \| \| \| \| \| \| \| \| \|  \| \| \|  \| Cohort \| \| Patients in cohort \| Patients with outcome \| Mean \| Standard Deviation \| Median \| \| \|  \| \| \|  \| 1 \| DM+AKD+DPP4i without heart failure \| 4,129 \| 699 \| 2.844 \| 8.746 \| 1 \| \| \|  \| \| \|  \| 2 \| DM+AKD without DPP4i without heart failure \| 4,273 \| 780 \| 2.414 \| 4.559 \| 1 \| \| \|  \| \| \| \| \| \| \| \| \| \| \| \| \|  \| \| \|  \|  \| \| t \| df \| p \|  \|  \|  \| \|  \| \| \|  \| **Test Statistics** \| \| 1.203 \| 1477 \| 0.229 \|  \|  \|  \|   **Proteinuria (ICD10CM:R80, R80.9, E11.29)**   \| **1 Mortality** \| \| \| \| \| \| \| \| \| \| \| \| \| \| \| \| \| --- \| --- \| --- \| --- \| --- \| --- \| --- \| --- \| --- \| --- \| --- \| --- \| --- \| --- \| --- \| --- \| \| **Risk analysis excluding patients with outcome prior to the time window** \| \| \| \| \| \| \| \| \| \| \| \| \| \| \|  \| \| Cohort \| \| \| Patients in cohort \| \| Patients with outcome \| \| Risk \| \| \| \| \| \| \|  \|  \| \| 1 \| DM+AKD+DPP4i with proteinuria 1 \| \| 621 \| \| 100 \| \| 0.161 \| \| \| \| \| \| \|  \|  \| \| 2 \| DM+AKD without DPP4i with proteinuria 1 \| \| 608 \| \| 123 \| \| 0.202 \| \| \| \| \| \| \|  \| \| \| \| \| \| \| \| \| \| \| \| \| \| \| \| \|  \|  \| \|  \| \| \|  \| \| 95% CI \| \| z \| p \|  \|  \| \| \| \|  \|  \| \| **Risk Difference** \| \| \| -0.041 \| \| (-0.084, 0.002) \| \| -1.877 \| 0.061 \|  \|  \| \| \| \|  \|  \| \| **Risk Ratio** \| \| \| 0.796 \| \| (0.627, 1.011) \| \| N/A \| N/A \|  \|  \| \| \| \|  \|  \| \| **Odds Ratio** \| \| \| 0.757 \| \| (0.565, 1.013) \| \| N/A \| N/A \|  \|  \| \| \| \|  \| \| \| \| \| \| \| \| \| \| \| \| \| \| \| \| \| **Kaplan - Meier survival analysis excluding patients with outcome prior to the time window** \| \| \| \| \| \| \| \| \| \| \| \| \| \| \|  \|  \| \| Cohort \| \| \| Patients in cohort \| \| Patients with outcome \| \| Median survival (days) \| Survival probability at end of time window \| \| \| \| \| \|  \|  \| \| 1 \| DM+AKD+DPP4i with proteinuria 1 \| \| 621 \| \| 100 \| \| -- \| 74.75% \| \| \| \| \| \|  \|  \| \| 2 \| DM+AKD without DPP4i with proteinuria 1 \| \| 608 \| \| 123 \| \| -- \| 69.10% \| \| \| \| \| \|  \| \| \| \| \| \| \| \| \| \| \| \| \| \| \| \| \|  \|  \| \|  \| \| \| χ^2^ \| \| df \| \| p \|  \|  \|  \| \| \| \|  \|  \| \| **Log-Rank Test** \| \| \| 4.414 \| \| 1 \| \| 0.036 \|  \|  \|  \| \| \| \|  \| \| \| \| \| \| \| \| \| \| \| \| \| \| \| \| \|  \|  \| \|  \| \| \| Hazard Ratio \| \| 95% CI \| \| χ^2^ \| df \| p \| \| \| \| \|  \|  \| \| **Hazard Ratio and Proportionality** \| \| \| 0.754 \| \| (0.579, 0.982) \| \| 0.596 \| 1 \| 0.440 \| \| \| \| \|  \| \| \| \| \| \| \| \| \| \| \| \| \| \| \| \| \| **Number of instances excluding patients with outcome prior to the time window** \| \| \| \| \| \| \| \| \| \| \| \| \| \| \|  \|  \| \| Cohort \| \| \| Patients in cohort \| \| Patients with outcome \| \| Mean \| Standard Deviation \| Median \| \| \| \| \|  \|  \| \| 1 \| DM+AKD+DPP4i with proteinuria 1 \| \| 621 \| \| 100 \| \| 1.160 \| 0.420 \| 1 \| \| \| \| \|  \|  \| \| 2 \| DM+AKD without DPP4i with proteinuria 1 \| \| 608 \| \| 123 \| \| 1.187 \| 0.431 \| 1 \| \| \| \| \|  \| \| \| \| \| \| \| \| \| \| \| \| \| \| \| \| \|  \|  \| \|  \| \| \| t \| \| df \| \| p \|  \|  \|  \| \| \| \|  \|  \| \| **Test Statistics** \| \| \| -0.470 \| \| 221 \| \| 0.639 \|  \|  \|  \| \| \| \|  \| \| \| \| \| \| \| \| \| \| \| \| \| \| \| \| \| **2 MAKE** \| \| \| \| \| \| \| \| \| \| \| \| \| \| \| \| \| **Risk analysis** \| \| \| \| \| \| \| \| \| \| \| \| \| \| \|  \| \| Cohort \| \| \| Patients in cohort \| \| Patients with outcome \| \| Risk \| \| \| \| \| \| \|  \|  \| \| 1 \| DM+AKD+DPP4i with proteinuria 1 \| \| 652 \| \| 118 \| \| 0.181 \| \| \| \| \| \| \|  \|  \| \| 2 \| DM+AKD without DPP4i with proteinuria 1 \| \| 652 \| \| 158 \| \| 0.242 \| \| \| \| \| \| \|  \| \| \| \| \| \| \| \| \| \| \| \| \| \| \| \| \|  \|  \| \|  \| \| \|  \| \| 95% CI \| \| z \| p \|  \|  \| \| \| \|  \|  \| \| **Risk Difference** \| \| \| -0.061 \| \| (-0.106, -0.017) \| \| -2.712 \| 0.007 \|  \|  \| \| \| \|  \|  \| \| **Risk Ratio** \| \| \| 0.747 \| \| (0.604, 0.924) \| \| N/A \| N/A \|  \|  \| \| \| \|  \|  \| \| **Odds Ratio** \| \| \| 0.691 \| \| (0.528, 0.903) \| \| N/A \| N/A \|  \|  \| \| \| \|  \| \| \| \| \| \| \| \| \| \| \| \| \| \| \| \| \| **Kaplan - Meier survival analysis** \| \| \| \| \| \| \| \| \| \| \| \| \| \| \|  \|  \| \| Cohort \| \| \| Patients in cohort \| \| Patients with outcome \| \| Median survival (days) \| Survival probability at end of time window \| \| \| \| \| \|  \|  \| \| 1 \| DM+AKD+DPP4i with proteinuria 1 \| \| 652 \| \| 118 \| \| -- \| 72.02% \| \| \| \| \| \|  \|  \| \| 2 \| DM+AKD without DPP4i with proteinuria 1 \| \| 652 \| \| 158 \| \| -- \| 64.20% \| \| \| \| \| \|  \| \| \| \| \| \| \| \| \| \| \| \| \| \| \| \| \|  \|  \| \|  \| \| \| χ^2^ \| \| df \| \| p \|  \|  \|  \| \| \| \|  \|  \| \| **Log-Rank Test** \| \| \| 10.172 \| \| 1 \| \| 0.001 \|  \|  \|  \| \| \| \|  \| \| \| \| \| \| \| \| \| \| \| \| \| \| \| \| \|  \|  \| \|  \| \| \| Hazard Ratio \| \| 95% CI \| \| χ^2^ \| df \| p \| \| \| \| \|  \|  \| \| **Hazard Ratio and Proportionality** \| \| \| 0.680 \| \| (0.536, 0.863) \| \| 2.625 \| 1 \| 0.105 \| \| \| \| \|  \| \| \| \| \| \| \| \| \| \| \| \| \| \| \| \| \|  \| \| \| \| \| \| \| \| \| \| \| \| \| \| \| \| \| **3 MACE** \| \| \| \| \| \| \| \| \| \| \| \| \| \| \| \| \| **Risk analysis excluding patients with outcome prior to the time window** \| \| \| \| \| \| \| \| \| \| \| \| \| \| \|  \| \| Cohort \| \| \| Patients in cohort \| \| Patients with outcome \| \| Risk \| \| \| \| \| \| \|  \|  \| \| 1 \| DM+AKD+DPP4i with proteinuria 1 \| \| 407 \| \| 109 \| \| 0.268 \| \| \| \| \| \| \|  \|  \| \| 2 \| DM+AKD without DPP4i with proteinuria 1 \| \| 417 \| \| 108 \| \| 0.259 \| \| \| \| \| \| \|  \| \| \| \| \| \| \| \| \| \| \| \| \| \| \| \| \|  \|  \| \|  \| \| \|  \| \| 95% CI \| \| z \| p \|  \|  \| \| \| \|  \|  \| \| **Risk Difference** \| \| \| 0.009 \| \| (-0.051, 0.069) \| \| 0.287 \| 0.774 \|  \|  \| \| \| \|  \|  \| \| **Risk Ratio** \| \| \| 1.034 \| \| (0.823, 1.299) \| \| N/A \| N/A \|  \|  \| \| \| \|  \|  \| \| **Odds Ratio** \| \| \| 1.047 \| \| (0.768, 1.427) \| \| N/A \| N/A \|  \|  \| \| \| \|  \| \| \| \| \| \| \| \| \| \| \| \| \| \| \| \| \| **Kaplan - Meier survival analysis excluding patients with outcome prior to the time window** \| \| \| \| \| \| \| \| \| \| \| \| \| \| \|  \|  \| \| Cohort \| \| \| Patients in cohort \| \| Patients with outcome \| \| Median survival (days) \| Survival probability at end of time window \| \| \| \| \| \|  \|  \| \| 1 \| DM+AKD+DPP4i with proteinuria 1 \| \| 407 \| \| 109 \| \| -- \| 60.93% \| \| \| \| \| \|  \|  \| \| 2 \| DM+AKD without DPP4i with proteinuria 1 \| \| 417 \| \| 108 \| \| -- \| 62.13% \| \| \| \| \| \|  \| \| \| \| \| \| \| \| \| \| \| \| \| \| \| \| \|  \|  \| \|  \| \| \| χ^2^ \| \| df \| \| p \|  \|  \|  \| \| \| \|  \|  \| \| **Log-Rank Test** \| \| \| 0.003 \| \| 1 \| \| 0.960 \|  \|  \|  \| \| \| \|  \| \| \| \| \| \| \| \| \| \| \| \| \| \| \| \| \|  \|  \| \|  \| \| \| Hazard Ratio \| \| 95% CI \| \| χ^2^ \| df \| p \| \| \| \| \|  \|  \| \| **Hazard Ratio and Proportionality** \| \| \| 1.007 \| \| (0.772, 1.314) \| \| 0.493 \| 1 \| 0.483 \| \| \| \| \|  \| \| \| \| \| \| \| \| \| \| \| \| \| \| \| \| \| **Number of instances excluding patients with outcome prior to the time window** \| \| \| \| \| \| \| \| \| \| \| \| \| \| \|  \|  \| \| Cohort \| \| \| Patients in cohort \| \| Patients with outcome \| \| Mean \| Standard Deviation \| Median \| \| \| \| \|  \|  \| \| 1 \| DM+AKD+DPP4i with proteinuria 1 \| \| 407 \| \| 109 \| \| 2.495 \| 3.463 \| 1 \| \| \| \| \|  \|  \| \| 2 \| DM+AKD without DPP4i with proteinuria 1 \| \| 417 \| \| 108 \| \| 2.630 \| 3.940 \| 1 \| \| \| \| \|  \| \| \| \| \| \| \| \| \| \| \| \| \| \| \| \| \|  \|  \| \|  \| \| \| t \| \| df \| \| p \|  \|  \|  \| \| \| \|  \|  \| \| **Test Statistics** \| \| \| -0.267 \| \| 215 \| \| 0.790 \|  \|  \|  \| \| \|   **Without proteinuria**   \| **1 Mortality** \| \| \| \| \| \| \| \| \| \| \| \| \| \| \| \| \| --- \| --- \| --- \| --- \| --- \| --- \| --- \| --- \| --- \| --- \| --- \| --- \| --- \| --- \| --- \| --- \| \| **Risk analysis excluding patients with outcome prior to the time window** \| \| \| \| \| \| \| \| \| \| \| \| \| \| \|  \| \| Cohort \| \| \| Patients in cohort \| \| Patients with outcome \| \| Risk \| \| \| \| \| \| \|  \|  \| \| 1 \| DM+AKD+DPP4i without proteinuria1 \| \| 6,772 \| \| 972 \| \| 0.144 \| \| \| \| \| \| \|  \|  \| \| 2 \| DM+AKD without DPP4i without proteinuria 1 \| \| 6,655 \| \| 1,019 \| \| 0.153 \| \| \| \| \| \| \|  \| \| \| \| \| \| \| \| \| \| \| \| \| \| \| \| \|  \|  \| \|  \| \| \|  \| \| 95% CI \| \| z \| p \|  \|  \| \| \| \|  \|  \| \| **Risk Difference** \| \| \| -0.010 \| \| (-0.022, 0.002) \| \| -1.563 \| 0.118 \|  \|  \| \| \| \|  \|  \| \| **Risk Ratio** \| \| \| 0.937 \| \| (0.864, 1.017) \| \| N/A \| N/A \|  \|  \| \| \| \|  \|  \| \| **Odds Ratio** \| \| \| 0.927 \| \| (0.843, 1.019) \| \| N/A \| N/A \|  \|  \| \| \| \|  \| \| \| \| \| \| \| \| \| \| \| \| \| \| \| \| \| **Kaplan - Meier survival analysis excluding patients with outcome prior to the time window** \| \| \| \| \| \| \| \| \| \| \| \| \| \| \|  \|  \| \| Cohort \| \| \| Patients in cohort \| \| Patients with outcome \| \| Median survival (days) \| Survival probability at end of time window \| \| \| \| \| \|  \|  \| \| 1 \| DM+AKD+DPP4i without proteinuria1 \| \| 6,772 \| \| 972 \| \| -- \| 74.73% \| \| \| \| \| \|  \|  \| \| 2 \| DM+AKD without DPP4i without proteinuria 1 \| \| 6,655 \| \| 1,019 \| \| -- \| 72.13% \| \| \| \| \| \|  \| \| \| \| \| \| \| \| \| \| \| \| \| \| \| \| \|  \|  \| \|  \| \| \| χ^2^ \| \| df \| \| p \|  \|  \|  \| \| \| \|  \|  \| \| **Log-Rank Test** \| \| \| 11.989 \| \| 1 \| \| 0.001 \|  \|  \|  \| \| \| \|  \| \| \| \| \| \| \| \| \| \| \| \| \| \| \| \| \|  \|  \| \|  \| \| \| Hazard Ratio \| \| 95% CI \| \| χ^2^ \| df \| p \| \| \| \| \|  \|  \| \| **Hazard Ratio and Proportionality** \| \| \| 0.856 \| \| (0.784, 0.935) \| \| 5.017 \| 1 \| 0.025 \| \| \| \| \|  \| \| \| \| \| \| \| \| \| \| \| \| \| \| \| \| \| **Number of instances excluding patients with outcome prior to the time window** \| \| \| \| \| \| \| \| \| \| \| \| \| \| \|  \|  \| \| Cohort \| \| \| Patients in cohort \| \| Patients with outcome \| \| Mean \| Standard Deviation \| Median \| \| \| \| \|  \|  \| \| 1 \| DM+AKD+DPP4i without proteinuria1 \| \| 6,772 \| \| 972 \| \| 1.201 \| 0.558 \| 1 \| \| \| \| \|  \|  \| \| 2 \| DM+AKD without DPP4i without proteinuria 1 \| \| 6,655 \| \| 1,019 \| \| 1.204 \| 0.551 \| 1 \| \| \| \| \|  \| \| \| \| \| \| \| \| \| \| \| \| \| \| \| \| \|  \|  \| \|  \| \| \| t \| \| df \| \| p \|  \|  \|  \| \| \| \|  \|  \| \| **Test Statistics** \| \| \| -0.141 \| \| 1989 \| \| 0.888 \|  \|  \|  \| \| \| \|  \| \| \| \| \| \| \| \| \| \| \| \| \| \| \| \| \| **2 MAKE** \| \| \| \| \| \| \| \| \| \| \| \| \| \| \| \| \| **Risk analysis** \| \| \| \| \| \| \| \| \| \| \| \| \| \| \|  \| \| Cohort \| \| \| Patients in cohort \| \| Patients with outcome \| \| Risk \| \| \| \| \| \| \|  \|  \| \| 1 \| DM+AKD+DPP4i without proteinuria1 \| \| 7,114 \| \| 1,082 \| \| 0.152 \| \| \| \| \| \| \|  \|  \| \| 2 \| DM+AKD without DPP4i without proteinuria 1 \| \| 7,114 \| \| 1,121 \| \| 0.158 \| \| \| \| \| \| \|  \| \| \| \| \| \| \| \| \| \| \| \| \| \| \| \| \|  \|  \| \|  \| \| \|  \| \| 95% CI \| \| z \| p \|  \|  \| \| \| \|  \|  \| \| **Risk Difference** \| \| \| -0.005 \| \| (-0.017, 0.006) \| \| -0.904 \| 0.366 \|  \|  \| \| \| \|  \|  \| \| **Risk Ratio** \| \| \| 0.965 \| \| (0.894, 1.042) \| \| N/A \| N/A \|  \|  \| \| \| \|  \|  \| \| **Odds Ratio** \| \| \| 0.959 \| \| (0.876, 1.050) \| \| N/A \| N/A \|  \|  \| \| \| \|  \| \| \| \| \| \| \| \| \| \| \| \| \| \| \| \| \| **Kaplan - Meier survival analysis** \| \| \| \| \| \| \| \| \| \| \| \| \| \| \|  \|  \| \| Cohort \| \| \| Patients in cohort \| \| Patients with outcome \| \| Median survival (days) \| Survival probability at end of time window \| \| \| \| \| \|  \|  \| \| 1 \| DM+AKD+DPP4i without proteinuria1 \| \| 7,114 \| \| 1,082 \| \| -- \| 73.40% \| \| \| \| \| \|  \|  \| \| 2 \| DM+AKD without DPP4i without proteinuria 1 \| \| 7,114 \| \| 1,121 \| \| -- \| 70.67% \| \| \| \| \| \|  \| \| \| \| \| \| \| \| \| \| \| \| \| \| \| \| \|  \|  \| \|  \| \| \| χ^2^ \| \| df \| \| p \|  \|  \|  \| \| \| \|  \|  \| \| **Log-Rank Test** \| \| \| 12.802 \| \| 1 \| \| 0.000 \|  \|  \|  \| \| \| \|  \| \| \| \| \| \| \| \| \| \| \| \| \| \| \| \| \|  \|  \| \|  \| \| \| Hazard Ratio \| \| 95% CI \| \| χ^2^ \| df \| p \| \| \| \| \|  \|  \| \| **Hazard Ratio and Proportionality** \| \| \| 0.859 \| \| (0.790, 0.933) \| \| 4.766 \| 1 \| 0.029 \| \| \| \| \| **3 MACE** \| \| \| \| \| \| \| \| \| \| \| \| \| \| \| \| \| **Risk analysis excluding patients with outcome prior to the time window** \| \| \| \| \| \| \| \| \| \| \| \| \| \| \|  \| \| Cohort \| \| \| Patients in cohort \| \| Patients with outcome \| \| Risk \| \| \| \| \| \| \|  \|  \| \| 1 \| DM+AKD+DPP4i without proteinuria1 \| \| 4,771 \| \| 930 \| \| 0.195 \| \| \| \| \| \| \|  \|  \| \| 2 \| DM+AKD without DPP4i without proteinuria 1 \| \| 4,825 \| \| 968 \| \| 0.201 \| \| \| \| \| \| \|  \| \| \| \| \| \| \| \| \| \| \| \| \| \| \| \| \|  \|  \| \|  \| \| \|  \| \| 95% CI \| \| z \| p \|  \|  \| \| \| \|  \|  \| \| **Risk Difference** \| \| \| -0.006 \| \| (-0.022, 0.010) \| \| -0.700 \| 0.484 \|  \|  \| \| \| \|  \|  \| \| **Risk Ratio** \| \| \| 0.972 \| \| (0.896, 1.053) \| \| N/A \| N/A \|  \|  \| \| \| \|  \|  \| \| **Odds Ratio** \| \| \| 0.965 \| \| (0.873, 1.067) \| \| N/A \| N/A \|  \|  \| \| \| \|  \| \| \| \| \| \| \| \| \| \| \| \| \| \| \| \| \| **Kaplan - Meier survival analysis excluding patients with outcome prior to the time window** \| \| \| \| \| \| \| \| \| \| \| \| \| \| \|  \|  \| \| Cohort \| \| \| Patients in cohort \| \| Patients with outcome \| \| Median survival (days) \| Survival probability at end of time window \| \| \| \| \| \|  \|  \| \| 1 \| DM+AKD+DPP4i without proteinuria1 \| \| 4,771 \| \| 930 \| \| -- \| 67.14% \| \| \| \| \| \|  \|  \| \| 2 \| DM+AKD without DPP4i without proteinuria 1 \| \| 4,825 \| \| 968 \| \| -- \| 64.87% \| \| \| \| \| \|  \| \| \| \| \| \| \| \| \| \| \| \| \| \| \| \| \|  \|  \| \|  \| \| \| χ^2^ \| \| df \| \| p \|  \|  \|  \| \| \| \|  \|  \| \| **Log-Rank Test** \| \| \| 5.481 \| \| 1 \| \| 0.019 \|  \|  \|  \| \| \| \|  \| \| \| \| \| \| \| \| \| \| \| \| \| \| \| \| \|  \|  \| \|  \| \| \| Hazard Ratio \| \| 95% CI \| \| χ^2^ \| df \| p \| \| \| \| \|  \|  \| \| **Hazard Ratio and Proportionality** \| \| \| 0.898 \| \| (0.821, 0.983) \| \| 1.498 \| 1 \| 0.221 \| \| \| \| \|  \| \| \| \| \| \| \| \| \| \| \| \| \| \| \| \| \| **Number of instances excluding patients with outcome prior to the time window** \| \| \| \| \| \| \| \| \| \| \| \| \| \| \|  \|  \| \| Cohort \| \| \| Patients in cohort \| \| Patients with outcome \| \| Mean \| Standard Deviation \| Median \| \| \| \| \|  \|  \| \| 1 \| DM+AKD+DPP4i without proteinuria1 \| \| 4,771 \| \| 930 \| \| 2.847 \| 6.783 \| 1 \| \| \| \| \|  \|  \| \| 2 \| DM+AKD without DPP4i without proteinuria 1 \| \| 4,825 \| \| 968 \| \| 2.419 \| 6.021 \| 1 \| \| \| \| \|  \| \| \| \| \| \| \| \| \| \| \| \| \| \| \| \| \|  \|  \| \|  \| \| \| t \| \| df \| \| p \|  \|  \|  \| \| \| \|  \|  \| \| **Test Statistics** \| \| \| 1.455 \| \| 1896 \| \| 0.146 \|  \|  \|  \| \| \|   **eGFR < 45 ml/min/1.73^2^ (eGFR, TNX:8001)**   \| **1 Mortality** \| \| \| \| \| \| \| \| \| \| \| \| \| --- \| --- \| --- \| --- \| --- \| --- \| --- \| --- \| --- \| --- \| --- \| --- \| \|  \| \| **Risk analysis excluding patients with outcome prior to the time window** \| \| \| \| \| \| \| \| \| \| \|  \|  \| \| \| Cohort \| \| Patients in cohort \| Patients with outcome \| Risk \| \| \| \| \|  \| \| \|  \| 1 \| DM+AKD+DPP4i eGFR < 45 1 \| 1,926 \| 397 \| 0.206 \| \| \| \| \|  \| \| \|  \| 2 \| DM+AKD without DPP4i eGFR < 45 1 \| 1,930 \| 457 \| 0.237 \| \| \| \| \|  \| \| \| \| \| \| \| \| \| \| \| \| \|  \| \| \|  \|  \| \|  \| 95% CI \| z \| p \|  \|  \| \|  \| \| \|  \| **Risk Difference** \| \| -0.031 \| (-0.057, -0.004) \| -2.293 \| 0.022 \|  \|  \| \|  \| \| \|  \| **Risk Ratio** \| \| 0.871 \| (0.773, 0.980) \| N/A \| N/A \|  \|  \| \|  \| \| \|  \| **Odds Ratio** \| \| 0.837 \| (0.719, 0.975) \| N/A \| N/A \|  \|  \| \|  \| \| \| \| \| \| \| \| \| \| \| \| \|  \| \| **Kaplan - Meier survival analysis excluding patients with outcome prior to the time window** \| \| \| \| \| \| \| \| \| \| \|  \| \| \|  \| Cohort \| \| Patients in cohort \| Patients with outcome \| Median survival (days) \| Survival probability at end of time window \| \| \| \|  \| \| \|  \| 1 \| DM+AKD+DPP4i eGFR < 45 1 \| 1,926 \| 397 \| -- \| 67.83% \| \| \| \|  \| \| \|  \| 2 \| DM+AKD without DPP4i eGFR < 45 1 \| 1,930 \| 457 \| -- \| 60.52% \| \| \| \|  \| \| \| \| \| \| \| \| \| \| \| \| \|  \| \| \|  \|  \| \| χ^2^ \| df \| p \|  \|  \|  \| \|  \| \| \|  \| **Log-Rank Test** \| \| 15.420 \| 1 \| 0.000 \|  \|  \|  \| \|  \| \| \| \| \| \| \| \| \| \| \| \| \|  \| \| \|  \|  \| \| Hazard Ratio \| 95% CI \| χ^2^ \| df \| p \| \| \|  \| \| \|  \| **Hazard Ratio and Proportionality** \| \| 0.764 \| (0.668, 0.874) \| 0.246 \| 1 \| 0.620 \| \| \|  \| \| \| \| \| \| \| \| \| \| \| \| \|  \| \| **Number of instances excluding patients with outcome prior to the time window** \| \| \| \| \| \| \| \| \| \| \|  \| \| \|  \| Cohort \| \| Patients in cohort \| Patients with outcome \| Mean \| Standard Deviation \| Median \| \| \|  \| \| \|  \| 1 \| DM+AKD+DPP4i eGFR < 45 1 \| 1,926 \| 397 \| 1.234 \| 0.601 \| 1 \| \| \|  \| \| \|  \| 2 \| DM+AKD without DPP4i eGFR < 45 1 \| 1,930 \| 457 \| 1.210 \| 0.413 \| 1 \| \| \|  \| \| \| \| \| \| \| \| \| \| \| \| \|  \| \| \|  \|  \| \| t \| df \| p \|  \|  \|  \| \|  \| \| \|  \| **Test Statistics** \| \| 0.692 \| 852 \| 0.489 \|  \|  \|  \| \|  \| \| \| \| \| \| \| \| \| \| \| \| \| **2 MAKE** \| \| \| \| \| \| \| \| \| \| \| \| \|  \| \| **Risk analysis** \| \| \| \| \| \| \| \| \| \| \|  \|  \| \| \| Cohort \| \| Patients in cohort \| Patients with outcome \| Risk \| \| \| \| \|  \| \| \|  \| 1 \| DM+AKD+DPP4i eGFR < 45 1 \| 2,038 \| 457 \| 0.224 \| \| \| \| \|  \| \| \|  \| 2 \| DM+AKD without DPP4i eGFR < 45 1 \| 2,038 \| 529 \| 0.260 \| \| \| \| \|  \| \| \| \| \| \| \| \| \| \| \| \| \|  \| \| \|  \|  \| \|  \| 95% CI \| z \| p \|  \|  \| \|  \| \| \|  \| **Risk Difference** \| \| -0.035 \| (-0.062, -0.009) \| -2.633 \| 0.008 \|  \|  \| \|  \| \| \|  \| **Risk Ratio** \| \| 0.864 \| (0.775, 0.963) \| N/A \| N/A \|  \|  \| \|  \| \| \|  \| **Odds Ratio** \| \| 0.825 \| (0.714, 0.952) \| N/A \| N/A \|  \|  \| \|  \| \| \| \| \| \| \| \| \| \| \| \| \|  \| \| **Kaplan - Meier survival analysis** \| \| \| \| \| \| \| \| \| \| \|  \| \| \|  \| Cohort \| \| Patients in cohort \| Patients with outcome \| Median survival (days) \| Survival probability at end of time window \| \| \| \|  \| \| \|  \| 1 \| DM+AKD+DPP4i eGFR < 45 1 \| 2,038 \| 457 \| -- \| 64.93% \| \| \| \|  \| \| \|  \| 2 \| DM+AKD without DPP4i eGFR < 45 1 \| 2,038 \| 529 \| -- \| 57.10% \| \| \| \|  \| \| \| \| \| \| \| \| \| \| \| \| \|  \| \| \|  \|  \| \| χ^2^ \| df \| p \|  \|  \|  \| \|  \| \| \|  \| **Log-Rank Test** \| \| 20.084 \| 1 \| 0.000 \|  \|  \|  \| \|  \| \| \| \| \| \| \| \| \| \| \| \| \|  \| \| \|  \|  \| \| Hazard Ratio \| 95% CI \| χ^2^ \| df \| p \| \| \|  \| \| \|  \| **Hazard Ratio and Proportionality** \| \| 0.752 \| (0.663, 0.852) \| 1.104 \| 1 \| 0.293 \| \| \|  \| \| \| \| \| \| \| \| \| \| \| \| \| **3 MACE** \| \| \| \| \| \| \| \| \| \| \| \| \|  \| \| **Risk analysis excluding patients with outcome prior to the time window** \| \| \| \| \| \| \| \| \| \| \|  \|  \| \| \| Cohort \| \| Patients in cohort \| Patients with outcome \| Risk \| \| \| \| \|  \| \| \|  \| 1 \| DM+AKD+DPP4i eGFR < 45 1 \| 1,279 \| 368 \| 0.288 \| \| \| \| \|  \| \| \|  \| 2 \| DM+AKD without DPP4i eGFR < 45 1 \| 1,304 \| 389 \| 0.298 \| \| \| \| \|  \| \| \| \| \| \| \| \| \| \| \| \| \|  \| \| \|  \|  \| \|  \| 95% CI \| z \| p \|  \|  \| \|  \| \| \|  \| **Risk Difference** \| \| -0.011 \| (-0.046, 0.025) \| -0.591 \| 0.554 \|  \|  \| \|  \| \| \|  \| **Risk Ratio** \| \| 0.965 \| (0.856, 1.087) \| N/A \| N/A \|  \|  \| \|  \| \| \|  \| **Odds Ratio** \| \| 0.950 \| (0.802, 1.126) \| N/A \| N/A \|  \|  \| \|  \| \| \| \| \| \| \| \| \| \| \| \| \|  \| \| **Kaplan - Meier survival analysis excluding patients with outcome prior to the time window** \| \| \| \| \| \| \| \| \| \| \|  \| \| \|  \| Cohort \| \| Patients in cohort \| Patients with outcome \| Median survival (days) \| Survival probability at end of time window \| \| \| \|  \| \| \|  \| 1 \| DM+AKD+DPP4i eGFR < 45 1 \| 1,279 \| 368 \| -- \| 56.99% \| \| \| \|  \| \| \|  \| 2 \| DM+AKD without DPP4i eGFR < 45 1 \| 1,304 \| 389 \| -- \| 53.64% \| \| \| \|  \| \| \| \| \| \| \| \| \| \| \| \| \|  \| \| \|  \|  \| \| χ^2^ \| df \| p \|  \|  \|  \| \|  \| \| \|  \| **Log-Rank Test** \| \| 5.176 \| 1 \| 0.023 \|  \|  \|  \| \|  \| \| \| \| \| \| \| \| \| \| \| \| \|  \| \| \|  \|  \| \| Hazard Ratio \| 95% CI \| χ^2^ \| df \| p \| \| \|  \| \| \|  \| **Hazard Ratio and Proportionality** \| \| 0.848 \| (0.735, 0.978) \| 3.158 \| 1 \| 0.076 \| \| \|  \| \| \| \| \| \| \| \| \| \| \| \| \|  \| \| **Number of instances excluding patients with outcome prior to the time window** \| \| \| \| \| \| \| \| \| \| \|  \| \| \|  \| Cohort \| \| Patients in cohort \| Patients with outcome \| Mean \| Standard Deviation \| Median \| \| \|  \| \| \|  \| 1 \| DM+AKD+DPP4i eGFR < 45 1 \| 1,279 \| 368 \| 2.932 \| 6.382 \| 1 \| \| \|  \| \| \|  \| 2 \| DM+AKD without DPP4i eGFR < 45 1 \| 1,304 \| 389 \| 2.111 \| 3.888 \| 1 \| \| \|  \| \| \| \| \| \| \| \| \| \| \| \| \|  \| \| \|  \|  \| \| t \| df \| p \|  \|  \|  \| \|  \| \| \|  \| **Test Statistics** \| \| 2.152 \| 755 \| 0.032 \|  \|  \|  \|   **eGFR > 45 ml/min/1.73^2^**   \| **1 Mortality** \| \| \| \| \| \| \| \| \| \| \| \| \| \| \| \| \| --- \| --- \| --- \| --- \| --- \| --- \| --- \| --- \| --- \| --- \| --- \| --- \| --- \| --- \| --- \| --- \| \| **Risk analysis excluding patients with outcome prior to the time window** \| \| \| \| \| \| \| \| \| \| \| \| \| \| \|  \| \| Cohort \| \| \| Patients in cohort \| \| Patients with outcome \| \| Risk \| \| \| \| \| \| \|  \|  \| \| 1 \| DM+AKD+DPP4i eGFR > 45 1 \| \| 4,420 \| \| 534 \| \| 0.121 \| \| \| \| \| \| \|  \|  \| \| 2 \| DM+AKD without DPP4i eGFR > 45 1 \| \| 4,437 \| \| 610 \| \| 0.137 \| \| \| \| \| \| \|  \| \| \| \| \| \| \| \| \| \| \| \| \| \| \| \| \|  \|  \| \|  \| \| \|  \| \| 95% CI \| \| z \| p \|  \|  \| \| \| \|  \|  \| \| **Risk Difference** \| \| \| -0.017 \| \| (-0.031, -0.003) \| \| -2.338 \| 0.019 \|  \|  \| \| \| \|  \|  \| \| **Risk Ratio** \| \| \| 0.879 \| \| (0.788, 0.979) \| \| N/A \| N/A \|  \|  \| \| \| \|  \|  \| \| **Odds Ratio** \| \| \| 0.862 \| \| (0.761, 0.976) \| \| N/A \| N/A \|  \|  \| \| \| \|  \| \| \| \| \| \| \| \| \| \| \| \| \| \| \| \| \| **Kaplan - Meier survival analysis excluding patients with outcome prior to the time window** \| \| \| \| \| \| \| \| \| \| \| \| \| \| \|  \|  \| \| Cohort \| \| \| Patients in cohort \| \| Patients with outcome \| \| Median survival (days) \| Survival probability at end of time window \| \| \| \| \| \|  \|  \| \| 1 \| DM+AKD+DPP4i eGFR > 45 1 \| \| 4,420 \| \| 534 \| \| -- \| 79.30% \| \| \| \| \| \|  \|  \| \| 2 \| DM+AKD without DPP4i eGFR > 45 1 \| \| 4,437 \| \| 610 \| \| -- \| 75.83% \| \| \| \| \| \|  \| \| \| \| \| \| \| \| \| \| \| \| \| \| \| \| \|  \|  \| \|  \| \| \| χ^2^ \| \| df \| \| p \|  \|  \|  \| \| \| \|  \|  \| \| **Log-Rank Test** \| \| \| 13.197 \| \| 1 \| \| 0.000 \|  \|  \|  \| \| \| \|  \| \| \| \| \| \| \| \| \| \| \| \| \| \| \| \| \|  \|  \| \|  \| \| \| Hazard Ratio \| \| 95% CI \| \| χ^2^ \| df \| p \| \| \| \| \|  \|  \| \| **Hazard Ratio and Proportionality** \| \| \| 0.807 \| \| (0.718, 0.906) \| \| 3.455 \| 1 \| 0.063 \| \| \| \| \|  \| \| \| \| \| \| \| \| \| \| \| \| \| \| \| \| \| **Number of instances excluding patients with outcome prior to the time window** \| \| \| \| \| \| \| \| \| \| \| \| \| \| \|  \|  \| \| Cohort \| \| \| Patients in cohort \| \| Patients with outcome \| \| Mean \| Standard Deviation \| Median \| \| \| \| \|  \|  \| \| 1 \| DM+AKD+DPP4i eGFR > 45 1 \| \| 4,420 \| \| 534 \| \| 1.223 \| 0.566 \| 1 \| \| \| \| \|  \|  \| \| 2 \| DM+AKD without DPP4i eGFR > 45 1 \| \| 4,437 \| \| 610 \| \| 1.164 \| 0.396 \| 1 \| \| \| \| \|  \| \| \| \| \| \| \| \| \| \| \| \| \| \| \| \| \|  \|  \| \|  \| \| \| t \| \| df \| \| p \|  \|  \|  \| \| \| \|  \|  \| \| **Test Statistics** \| \| \| 2.059 \| \| 1142 \| \| 0.040 \|  \|  \|  \| \| \| \|  \| \| \| \| \| \| \| \| \| \| \| \| \| \| \| \| \| **2 MAKE** \| \| \| \| \| \| \| \| \| \| \| \| \| \| \| \| \| **Risk analysis** \| \| \| \| \| \| \| \| \| \| \| \| \| \| \|  \| \| Cohort \| \| \| Patients in cohort \| \| Patients with outcome \| \| Risk \| \| \| \| \| \| \|  \|  \| \| 1 \| DM+AKD+DPP4i eGFR > 45 1 \| \| 4,685 \| \| 580 \| \| 0.124 \| \| \| \| \| \| \|  \|  \| \| 2 \| DM+AKD without DPP4i eGFR > 45 1 \| \| 4,685 \| \| 644 \| \| 0.137 \| \| \| \| \| \| \|  \| \| \| \| \| \| \| \| \| \| \| \| \| \| \| \| \|  \|  \| \|  \| \| \|  \| \| 95% CI \| \| z \| p \|  \|  \| \| \| \|  \|  \| \| **Risk Difference** \| \| \| -0.014 \| \| (-0.027, -0.000) \| \| -1.962 \| 0.050 \|  \|  \| \| \| \|  \|  \| \| **Risk Ratio** \| \| \| 0.901 \| \| (0.811, 1.000) \| \| N/A \| N/A \|  \|  \| \| \| \|  \|  \| \| **Odds Ratio** \| \| \| 0.887 \| \| (0.786, 1.000) \| \| N/A \| N/A \|  \|  \| \| \| \|  \| \| \| \| \| \| \| \| \| \| \| \| \| \| \| \| \| **Kaplan - Meier survival analysis** \| \| \| \| \| \| \| \| \| \| \| \| \| \| \|  \|  \| \| Cohort \| \| \| Patients in cohort \| \| Patients with outcome \| \| Median survival (days) \| Survival probability at end of time window \| \| \| \| \| \|  \|  \| \| 1 \| DM+AKD+DPP4i eGFR > 45 1 \| \| 4,685 \| \| 580 \| \| -- \| 78.77% \| \| \| \| \| \|  \|  \| \| 2 \| DM+AKD without DPP4i eGFR > 45 1 \| \| 4,685 \| \| 644 \| \| -- \| 75.50% \| \| \| \| \| \|  \| \| \| \| \| \| \| \| \| \| \| \| \| \| \| \| \|  \|  \| \|  \| \| \| χ^2^ \| \| df \| \| p \|  \|  \|  \| \| \| \|  \|  \| \| **Log-Rank Test** \| \| \| 12.698 \| \| 1 \| \| 0.000 \|  \|  \|  \| \| \| \|  \| \| \| \| \| \| \| \| \| \| \| \| \| \| \| \| \|  \|  \| \|  \| \| \| Hazard Ratio \| \| 95% CI \| \| χ^2^ \| df \| p \| \| \| \| \|  \|  \| \| **Hazard Ratio and Proportionality** \| \| \| 0.816 \| \| (0.729, 0.913) \| \| 3.866 \| 1 \| 0.049 \| \| \| \| \|  \| \| \| \| \| \| \| \| \| \| \| \| \| \| \| \| \| **3 MACE** \| \| \| \| \| \| \| \| \| \| \| \| \| \| \| \| \| **Risk analysis excluding patients with outcome prior to the time window** \| \| \| \| \| \| \| \| \| \| \| \| \| \| \|  \| \| Cohort \| \| \| Patients in cohort \| \| Patients with outcome \| \| Risk \| \| \| \| \| \| \|  \|  \| \| 1 \| DM+AKD+DPP4i eGFR > 45 1 \| \| 3,202 \| \| 563 \| \| 0.176 \| \| \| \| \| \| \|  \|  \| \| 2 \| DM+AKD without DPP4i eGFR > 45 1 \| \| 3,328 \| \| 607 \| \| 0.182 \| \| \| \| \| \| \|  \| \| \| \| \| \| \| \| \| \| \| \| \| \| \| \| \|  \|  \| \|  \| \| \|  \| \| 95% CI \| \| z \| p \|  \|  \| \| \| \|  \|  \| \| **Risk Difference** \| \| \| -0.007 \| \| (-0.025, 0.012) \| \| -0.691 \| 0.489 \|  \|  \| \| \| \|  \|  \| \| **Risk Ratio** \| \| \| 0.964 \| \| (0.869, 1.070) \| \| N/A \| N/A \|  \|  \| \| \| \|  \|  \| \| **Odds Ratio** \| \| \| 0.956 \| \| (0.843, 1.085) \| \| N/A \| N/A \|  \|  \| \| \| \|  \| \| \| \| \| \| \| \| \| \| \| \| \| \| \| \| \| **Kaplan - Meier survival analysis excluding patients with outcome prior to the time window** \| \| \| \| \| \| \| \| \| \| \| \| \| \| \|  \|  \| \| Cohort \| \| \| Patients in cohort \| \| Patients with outcome \| \| Median survival (days) \| Survival probability at end of time window \| \| \| \| \| \|  \|  \| \| 1 \| DM+AKD+DPP4i eGFR > 45 1 \| \| 3,202 \| \| 563 \| \| -- \| 71.39% \| \| \| \| \| \|  \|  \| \| 2 \| DM+AKD without DPP4i eGFR > 45 1 \| \| 3,328 \| \| 607 \| \| -- \| 68.32% \| \| \| \| \| \|  \| \| \| \| \| \| \| \| \| \| \| \| \| \| \| \| \|  \|  \| \|  \| \| \| χ^2^ \| \| df \| \| p \|  \|  \|  \| \| \| \|  \|  \| \| **Log-Rank Test** \| \| \| 4.738 \| \| 1 \| \| 0.030 \|  \|  \|  \| \| \| \|  \| \| \| \| \| \| \| \| \| \| \| \| \| \| \| \| \|  \|  \| \|  \| \| \| Hazard Ratio \| \| 95% CI \| \| χ^2^ \| df \| p \| \| \| \| \|  \|  \| \| **Hazard Ratio and Proportionality** \| \| \| 0.880 \| \| (0.785, 0.987) \| \| 0.098 \| 1 \| 0.754 \| \| \| \| \|  \| \| \| \| \| \| \| \| \| \| \| \| \| \| \| \| \| **Number of instances excluding patients with outcome prior to the time window** \| \| \| \| \| \| \| \| \| \| \| \| \| \| \|  \|  \| \| Cohort \| \| \| Patients in cohort \| \| Patients with outcome \| \| Mean \| Standard Deviation \| Median \| \| \| \| \|  \|  \| \| 1 \| DM+AKD+DPP4i eGFR > 45 1 \| \| 3,202 \| \| 563 \| \| 3.432 \| 10.011 \| 1 \| \| \| \| \|  \|  \| \| 2 \| DM+AKD without DPP4i eGFR > 45 1 \| \| 3,328 \| \| 607 \| \| 2.326 \| 3.937 \| 1 \| \| \| \| \|  \| \| \| \| \| \| \| \| \| \| \| \| \| \| \| \| \|  \|  \| \|  \| \| \| t \| \| df \| \| p \|  \|  \|  \| \| \| \|  \|  \| \| **Test Statistics** \| \| \| 2.519 \| \| 1168 \| \| 0.012 \|  \|  \|  \| \| \|   **eGFR < 30 ml/min/1.73^2^**   \| **1 Mortality** \| \| \| \| \| \| \| \| \| \| \| \| \| \| \| \| \| --- \| --- \| --- \| --- \| --- \| --- \| --- \| --- \| --- \| --- \| --- \| --- \| --- \| --- \| --- \| --- \| \| **Risk analysis excluding patients with outcome prior to the time window** \| \| \| \| \| \| \| \| \| \| \| \| \| \| \|  \| \| Cohort \| \| \| Patients in cohort \| \| Patients with outcome \| \| Risk \| \| \| \| \| \| \|  \|  \| \| 1 \| DM+AKD+DPP4i eGFR < 30 1 \| \| 970 \| \| 222 \| \| 0.229 \| \| \| \| \| \| \|  \|  \| \| 2 \| DM+AKD without DPP4i eGFR < 30 1 \| \| 975 \| \| 243 \| \| 0.249 \| \| \| \| \| \| \|  \| \| \| \| \| \| \| \| \| \| \| \| \| \| \| \| \|  \|  \| \|  \| \| \|  \| \| 95% CI \| \| z \| p \|  \|  \| \| \| \|  \|  \| \| **Risk Difference** \| \| \| -0.020 \| \| (-0.058, 0.018) \| \| -1.053 \| 0.292 \|  \|  \| \| \| \|  \|  \| \| **Risk Ratio** \| \| \| 0.918 \| \| (0.783, 1.076) \| \| N/A \| N/A \|  \|  \| \| \| \|  \|  \| \| **Odds Ratio** \| \| \| 0.894 \| \| (0.726, 1.101) \| \| N/A \| N/A \|  \|  \| \| \| \|  \| \| \| \| \| \| \| \| \| \| \| \| \| \| \| \| \| **Kaplan - Meier survival analysis excluding patients with outcome prior to the time window** \| \| \| \| \| \| \| \| \| \| \| \| \| \| \|  \|  \| \| Cohort \| \| \| Patients in cohort \| \| Patients with outcome \| \| Median survival (days) \| Survival probability at end of time window \| \| \| \| \| \|  \|  \| \| 1 \| DM+AKD+DPP4i eGFR < 30 1 \| \| 970 \| \| 222 \| \| -- \| 63.55% \| \| \| \| \| \|  \|  \| \| 2 \| DM+AKD without DPP4i eGFR < 30 1 \| \| 975 \| \| 243 \| \| -- \| 54.81% \| \| \| \| \| \|  \| \| \| \| \| \| \| \| \| \| \| \| \| \| \| \| \|  \|  \| \|  \| \| \| χ^2^ \| \| df \| \| p \|  \|  \|  \| \| \| \|  \|  \| \| **Log-Rank Test** \| \| \| 6.454 \| \| 1 \| \| 0.011 \|  \|  \|  \| \| \| \|  \| \| \| \| \| \| \| \| \| \| \| \| \| \| \| \| \|  \|  \| \|  \| \| \| Hazard Ratio \| \| 95% CI \| \| χ^2^ \| df \| p \| \| \| \| \|  \|  \| \| **Hazard Ratio and Proportionality** \| \| \| 0.790 \| \| (0.659, 0.948) \| \| 1.193 \| 1 \| 0.275 \| \| \| \| \|  \| \| \| \| \| \| \| \| \| \| \| \| \| \| \| \| \| **Number of instances excluding patients with outcome prior to the time window** \| \| \| \| \| \| \| \| \| \| \| \| \| \| \|  \|  \| \| Cohort \| \| \| Patients in cohort \| \| Patients with outcome \| \| Mean \| Standard Deviation \| Median \| \| \| \| \|  \|  \| \| 1 \| DM+AKD+DPP4i eGFR < 30 1 \| \| 970 \| \| 222 \| \| 1.198 \| 0.453 \| 1 \| \| \| \| \|  \|  \| \| 2 \| DM+AKD without DPP4i eGFR < 30 1 \| \| 975 \| \| 243 \| \| 1.198 \| 0.409 \| 1 \| \| \| \| \|  \| \| \| \| \| \| \| \| \| \| \| \| \| \| \| \| \|  \|  \| \|  \| \| \| t \| \| df \| \| p \|  \|  \|  \| \| \| \|  \|  \| \| **Test Statistics** \| \| \| 0.017 \| \| 463 \| \| 0.987 \|  \|  \|  \| \| \| \|  \| \| \| \| \| \| \| \| \| \| \| \| \| \| \| \| \| **2 MAKE** \| \| \| \| \| \| \| \| \| \| \| \| \| \| \| \| \| **Risk analysis** \| \| \| \| \| \| \| \| \| \| \| \| \| \| \|  \| \| Cohort \| \| \| Patients in cohort \| \| Patients with outcome \| \| Risk \| \| \| \| \| \| \|  \|  \| \| 1 \| DM+AKD+DPP4i eGFR < 30 1 \| \| 1,029 \| \| 262 \| \| 0.255 \| \| \| \| \| \| \|  \|  \| \| 2 \| DM+AKD without DPP4i eGFR < 30 1 \| \| 1,029 \| \| 283 \| \| 0.275 \| \| \| \| \| \| \|  \| \| \| \| \| \| \| \| \| \| \| \| \| \| \| \| \|  \|  \| \|  \| \| \|  \| \| 95% CI \| \| z \| p \|  \|  \| \| \| \|  \|  \| \| **Risk Difference** \| \| \| -0.020 \| \| (-0.059, 0.018) \| \| -1.049 \| 0.294 \|  \|  \| \| \| \|  \|  \| \| **Risk Ratio** \| \| \| 0.926 \| \| (0.802, 1.069) \| \| N/A \| N/A \|  \|  \| \| \| \|  \|  \| \| **Odds Ratio** \| \| \| 0.900 \| \| (0.740, 1.095) \| \| N/A \| N/A \|  \|  \| \| \| \|  \| \| \| \| \| \| \| \| \| \| \| \| \| \| \| \| \| **Kaplan - Meier survival analysis** \| \| \| \| \| \| \| \| \| \| \| \| \| \| \|  \|  \| \| Cohort \| \| \| Patients in cohort \| \| Patients with outcome \| \| Median survival (days) \| Survival probability at end of time window \| \| \| \| \| \|  \|  \| \| 1 \| DM+AKD+DPP4i eGFR < 30 1 \| \| 1,029 \| \| 262 \| \| -- \| 59.99% \| \| \| \| \| \|  \|  \| \| 2 \| DM+AKD without DPP4i eGFR < 30 1 \| \| 1,029 \| \| 283 \| \| -- \| 50.84% \| \| \| \| \| \|  \| \| \| \| \| \| \| \| \| \| \| \| \| \| \| \| \|  \|  \| \|  \| \| \| χ^2^ \| \| df \| \| p \|  \|  \|  \| \| \| \|  \|  \| \| **Log-Rank Test** \| \| \| 7.544 \| \| 1 \| \| 0.006 \|  \|  \|  \| \| \| \|  \| \| \| \| \| \| \| \| \| \| \| \| \| \| \| \| \|  \|  \| \|  \| \| \| Hazard Ratio \| \| 95% CI \| \| χ^2^ \| df \| p \| \| \| \| \|  \|  \| \| **Hazard Ratio and Proportionality** \| \| \| 0.791 \| \| (0.668, 0.935) \| \| 0.863 \| 1 \| 0.353 \| \| \| \| \| **3 MACE** \| \| \| \| \| \| \| \| \| \| \| \| \| \| \| \| \| **Risk analysis excluding patients with outcome prior to the time window** \| \| \| \| \| \| \| \| \| \| \| \| \| \| \|  \| \| Cohort \| \| \| Patients in cohort \| \| Patients with outcome \| \| Risk \| \| \| \| \| \| \|  \|  \| \| 1 \| DM+AKD+DPP4i eGFR < 30 1 \| \| 635 \| \| 191 \| \| 0.301 \| \| \| \| \| \| \|  \|  \| \| 2 \| DM+AKD without DPP4i eGFR < 30 1 \| \| 628 \| \| 193 \| \| 0.307 \| \| \| \| \| \| \|  \| \| \| \| \| \| \| \| \| \| \| \| \| \| \| \| \|  \|  \| \|  \| \| \|  \| \| 95% CI \| \| z \| p \|  \|  \| \| \| \|  \|  \| \| **Risk Difference** \| \| \| -0.007 \| \| (-0.057, 0.044) \| \| -0.253 \| 0.801 \|  \|  \| \| \| \|  \|  \| \| **Risk Ratio** \| \| \| 0.979 \| \| (0.828, 1.156) \| \| N/A \| N/A \|  \|  \| \| \| \|  \|  \| \| **Odds Ratio** \| \| \| 0.970 \| \| (0.763, 1.232) \| \| N/A \| N/A \|  \|  \| \| \| \|  \| \| \| \| \| \| \| \| \| \| \| \| \| \| \| \| \| **Kaplan - Meier survival analysis excluding patients with outcome prior to the time window** \| \| \| \| \| \| \| \| \| \| \| \| \| \| \|  \|  \| \| Cohort \| \| \| Patients in cohort \| \| Patients with outcome \| \| Median survival (days) \| Survival probability at end of time window \| \| \| \| \| \|  \|  \| \| 1 \| DM+AKD+DPP4i eGFR < 30 1 \| \| 635 \| \| 191 \| \| -- \| 52.61% \| \| \| \| \| \|  \|  \| \| 2 \| DM+AKD without DPP4i eGFR < 30 1 \| \| 628 \| \| 193 \| \| 1780 \| 47.67% \| \| \| \| \| \|  \| \| \| \| \| \| \| \| \| \| \| \| \| \| \| \| \|  \|  \| \|  \| \| \| χ^2^ \| \| df \| \| p \|  \|  \|  \| \| \| \|  \|  \| \| **Log-Rank Test** \| \| \| 1.854 \| \| 1 \| \| 0.173 \|  \|  \|  \| \| \| \|  \| \| \| \| \| \| \| \| \| \| \| \| \| \| \| \| \|  \|  \| \|  \| \| \| Hazard Ratio \| \| 95% CI \| \| χ^2^ \| df \| p \| \| \| \| \|  \|  \| \| **Hazard Ratio and Proportionality** \| \| \| 0.870 \| \| (0.713, 1.063) \| \| 0.011 \| 1 \| 0.916 \| \| \| \| \|  \| \| \| \| \| \| \| \| \| \| \| \| \| \| \| \| \| **Number of instances excluding patients with outcome prior to the time window** \| \| \| \| \| \| \| \| \| \| \| \| \| \| \|  \|  \| \| Cohort \| \| \| Patients in cohort \| \| Patients with outcome \| \| Mean \| Standard Deviation \| Median \| \| \| \| \|  \|  \| \| 1 \| DM+AKD+DPP4i eGFR < 30 1 \| \| 635 \| \| 191 \| \| 2.225 \| 2.735 \| 1 \| \| \| \| \|  \|  \| \| 2 \| DM+AKD without DPP4i eGFR < 30 1 \| \| 628 \| \| 193 \| \| 1.891 \| 3.146 \| 1 \| \| \| \| \|  \| \| \| \| \| \| \| \| \| \| \| \| \| \| \| \| \|  \|  \| \|  \| \| \| t \| \| df \| \| p \|  \|  \|  \| \| \| \|  \|  \| \| **Test Statistics** \| \| \| 1.109 \| \| 382 \| \| 0.268 \|  \|  \|  \| \| \|   **eGFR > 30 ml/min/1.73^2^**   \| **1 Mortality** \| \| \| \| \| \| \| \| \| \| \| \| \| \| --- \| --- \| --- \| --- \| --- \| --- \| --- \| --- \| --- \| --- \| --- \| --- \| --- \| \|  \| \| **Risk analysis excluding patients with outcome prior to the time window** \| \| \| \| \| \| \| \| \| \| \| \|  \|  \| \| \| Cohort \| \| \| Patients in cohort \| Patients with outcome \| Risk \| \| \| \| \|  \| \| \|  \| 1 \| \| DM+AKD+DPP4i eGFR > 30 1 \| 4,324 \| 664 \| 0.154 \| \| \| \| \|  \| \| \|  \| 2 \| \| DM+AKD without DPP4i eGFR > 30 1 \| 4,367 \| 788 \| 0.180 \| \| \| \| \|  \| \| \| \| \| \| \| \| \| \| \| \| \| \|  \| \| \|  \|  \| \| \|  \| 95% CI \| z \| p \|  \|  \| \|  \| \| \|  \| **Risk Difference** \| \| \| -0.027 \| (-0.043, -0.011) \| -3.359 \| 0.001 \|  \|  \| \|  \| \| \|  \| **Risk Ratio** \| \| \| 0.851 \| (0.774, 0.935) \| N/A \| N/A \|  \|  \| \|  \| \| \|  \| **Odds Ratio** \| \| \| 0.824 \| (0.736, 0.923) \| N/A \| N/A \|  \|  \| \|  \| \| \| \| \| \| \| \| \| \| \| \| \| \|  \| \| **Kaplan - Meier survival analysis excluding patients with outcome prior to the time window** \| \| \| \| \| \| \| \| \| \| \| \|  \| \| \|  \| Cohort \| \| \| Patients in cohort \| Patients with outcome \| Median survival (days) \| Survival probability at end of time window \| \| \| \|  \| \| \|  \| 1 \| \| DM+AKD+DPP4i eGFR > 30 1 \| 4,324 \| 664 \| -- \| 76.22% \| \| \| \|  \| \| \|  \| 2 \| \| DM+AKD without DPP4i eGFR > 30 1 \| 4,367 \| 788 \| -- \| 70.91% \| \| \| \|  \| \| \| \| \| \| \| \| \| \| \| \| \| \|  \| \| \|  \|  \| \| \| χ^2^ \| df \| p \|  \|  \|  \| \|  \| \| \|  \| **Log-Rank Test** \| \| \| 24.839 \| 1 \| 0.000 \|  \|  \|  \| \|  \| \| \| \| \| \| \| \| \| \| \| \| \| \|  \| \| \|  \|  \| \| \| Hazard Ratio \| 95% CI \| χ^2^ \| df \| p \| \| \|  \| \| \|  \| **Hazard Ratio and Proportionality** \| \| \| 0.770 \| (0.694, 0.853) \| 1.625 \| 1 \| 0.202 \| \| \|  \| \| \| \| \| \| \| \| \| \| \| \| \| \|  \| \| **Number of instances excluding patients with outcome prior to the time window** \| \| \| \| \| \| \| \| \| \| \| \|  \| \| \|  \| Cohort \| \| \| Patients in cohort \| Patients with outcome \| Mean \| Standard Deviation \| Median \| \| \|  \| \| \|  \| 1 \| \| DM+AKD+DPP4i eGFR > 30 1 \| 4,324 \| 664 \| 1.221 \| 0.525 \| 1 \| \| \|  \| \| \|  \| 2 \| \| DM+AKD without DPP4i eGFR > 30 1 \| 4,367 \| 788 \| 1.226 \| 0.645 \| 1 \| \| \|  \| \| \| \| \| \| \| \| \| \| \| \| \| \|  \| \| \|  \|  \| \| \| t \| df \| p \|  \|  \|  \| \|  \| \| \|  \| **Test Statistics** \| \| \| -0.144 \| 1450 \| 0.885 \|  \|  \|  \| \|  \| \| \| \| \| \| \| \| \| \| \| \| \| \|  \| \|  \| \| \|  \| \| \| \| \| \| \| \| \| **2 MAKE** \| \| \| \| \| \| \| \| \| \| \| \| \| \|  \| \| **Risk analysis** \| \| \| \| \| \| \| \| \| \| \| \|  \|  \| \| \| Cohort \| \| \| Patients in cohort \| Patients with outcome \| Risk \| \| \| \| \|  \| \| \|  \| 1 \| \| DM+AKD+DPP4i eGFR > 30 1 \| 4,560 \| 735 \| 0.161 \| \| \| \| \|  \| \| \|  \| 2 \| \| DM+AKD without DPP4i eGFR > 30 1 \| 4,560 \| 847 \| 0.186 \| \| \| \| \|  \| \| \| \| \| \| \| \| \| \| \| \| \| \|  \| \| \|  \|  \| \| \|  \| 95% CI \| z \| p \|  \|  \| \|  \| \| \|  \| **Risk Difference** \| \| \| -0.025 \| (-0.040, -0.009) \| -3.097 \| 0.002 \|  \|  \| \|  \| \| \|  \| **Risk Ratio** \| \| \| 0.868 \| (0.793, 0.949) \| N/A \| N/A \|  \|  \| \|  \| \| \|  \| **Odds Ratio** \| \| \| 0.842 \| (0.756, 0.939) \| N/A \| N/A \|  \|  \| \|  \| \| \| \| \| \| \| \| \| \| \| \| \| \|  \| \| **Kaplan - Meier survival analysis** \| \| \| \| \| \| \| \| \| \| \| \|  \| \| \|  \| Cohort \| \| \| Patients in cohort \| Patients with outcome \| Median survival (days) \| Survival probability at end of time window \| \| \| \|  \| \| \|  \| 1 \| \| DM+AKD+DPP4i eGFR > 30 1 \| 4,560 \| 735 \| -- \| 74.93% \| \| \| \|  \| \| \|  \| 2 \| \| DM+AKD without DPP4i eGFR > 30 1 \| 4,560 \| 847 \| -- \| 70.11% \| \| \| \|  \| \| \| \| \| \| \| \| \| \| \| \| \| \|  \| \| \|  \|  \| \| \| χ^2^ \| df \| p \|  \|  \|  \| \|  \| \| \|  \| **Log-Rank Test** \| \| \| 21.416 \| 1 \| 0.000 \|  \|  \|  \| \|  \| \| \| \| \| \| \| \| \| \| \| \| \| \|  \| \| \|  \|  \| \| \| Hazard Ratio \| 95% CI \| χ^2^ \| df \| p \| \| \|  \| \| \|  \| **Hazard Ratio and Proportionality** \| \| \| 0.792 \| (0.718, 0.875) \| 1.804 \| 1 \| 0.179 \| \| \|  \| \| \| \| \| \| \| \| \| \| \| \| \| \| **3 MACE** \| \| \| \| \| \| \| \| \| \| \| \| \| \|  \| \| **Risk analysis excluding patients with outcome prior to the time window** \| \| \| \| \| \| \| \| \| \| \| \|  \|  \| \| \| Cohort \| \| \| Patients in cohort \| Patients with outcome \| Risk \| \| \| \| \|  \| \| \|  \| 1 \| \| DM+AKD+DPP4i eGFR > 30 1 \| 3,057 \| 693 \| 0.227 \| \| \| \| \|  \| \| \|  \| 2 \| \| DM+AKD without DPP4i eGFR > 30 1 \| 3,077 \| 727 \| 0.236 \| \| \| \| \|  \| \| \| \| \| \| \| \| \| \| \| \| \| \|  \| \| \|  \|  \| \| \|  \| 95% CI \| z \| p \|  \|  \| \|  \| \| \|  \| **Risk Difference** \| \| \| -0.010 \| (-0.031, 0.012) \| -0.889 \| 0.374 \|  \|  \| \|  \| \| \|  \| **Risk Ratio** \| \| \| 0.959 \| (0.876, 1.051) \| N/A \| N/A \|  \|  \| \|  \| \| \|  \| **Odds Ratio** \| \| \| 0.948 \| (0.842, 1.067) \| N/A \| N/A \|  \|  \| \|  \| \| \| \| \| \| \| \| \| \| \| \| \| \|  \| \| **Kaplan - Meier survival analysis excluding patients with outcome prior to the time window** \| \| \| \| \| \| \| \| \| \| \| \|  \| \| \|  \| Cohort \| \| \| Patients in cohort \| Patients with outcome \| Median survival (days) \| Survival probability at end of time window \| \| \| \|  \| \| \|  \| 1 \| \| DM+AKD+DPP4i eGFR > 30 1 \| 3,057 \| 693 \| -- \| 66.84% \| \| \| \|  \| \| \|  \| 2 \| \| DM+AKD without DPP4i eGFR > 30 1 \| 3,077 \| 727 \| -- \| 62.84% \| \| \| \|  \| \| \| \| \| \| \| \| \| \| \| \| \| \|  \| \| \|  \|  \| \| \| χ^2^ \| df \| p \|  \|  \|  \| \|  \| \| \|  \| **Log-Rank Test** \| \| \| 6.826 \| 1 \| 0.009 \|  \|  \|  \| \|  \| \| \| \| \| \| \| \| \| \| \| \| \| \|  \| \| \|  \|  \| \| \| Hazard Ratio \| 95% CI \| χ^2^ \| df \| p \| \| \|  \| \| \|  \| **Hazard Ratio and Proportionality** \| \| \| 0.871 \| (0.785, 0.966) \| 0.103 \| 1 \| 0.749 \| \| \|  \| \| \| \| \| \| \| \| \| \| \| \| \| \|  \| \| **Number of instances excluding patients with outcome prior to the time window** \| \| \| \| \| \| \| \| \| \| \| \|  \| \| \|  \| Cohort \| \| \| Patients in cohort \| Patients with outcome \| Mean \| Standard Deviation \| Median \| \| \|  \| \| \|  \| 1 \| \| DM+AKD+DPP4i eGFR > 30 1 \| 3,057 \| 693 \| 3.623 \| 7.743 \| 1 \| \| \|  \| \| \|  \| 2 \| \| DM+AKD without DPP4i eGFR > 30 1 \| 3,077 \| 727 \| 2.648 \| 5.788 \| 1 \| \| \|  \| \| \| \| \| \| \| \| \| \| \| \| \| \|  \| \| \|  \|  \| \| \| t \| df \| p \|  \|  \|  \| \|  \| \| \|  \| **Test Statistics** \| \| \| 2.697 \| 1418 \| 0.007 \|  \|  \|  \|   **BMI < 30 kg/m^2^ (BMI, TNX:9083)**   \| **1 Mortality** \| \| \| \| \| \| \| \| \| \| \| \| \| --- \| --- \| --- \| --- \| --- \| --- \| --- \| --- \| --- \| --- \| --- \| --- \| \|  \| \| **Risk analysis excluding patients with outcome prior to the time window** \| \| \| \| \| \| \| \| \| \| \|  \|  \| \| \| Cohort \| \| Patients in cohort \| Patients with outcome \| Risk \| \| \| \| \|  \| \| \|  \| 1 \| DM+AKD+DPP4i BMI < 30 1 \| 1,371 \| 254 \| 0.185 \| \| \| \| \|  \| \| \|  \| 2 \| DM+AKD without DPP4i BMI < 30 1 \| 1,377 \| 297 \| 0.216 \| \| \| \| \|  \| \| \| \| \| \| \| \| \| \| \| \| \|  \| \| \|  \|  \| \|  \| 95% CI \| z \| p \|  \|  \| \|  \| \| \|  \| **Risk Difference** \| \| -0.030 \| (-0.060, -0.001) \| -1.991 \| 0.046 \|  \|  \| \|  \| \| \|  \| **Risk Ratio** \| \| 0.859 \| (0.739, 0.998) \| N/A \| N/A \|  \|  \| \|  \| \| \|  \| **Odds Ratio** \| \| 0.827 \| (0.686, 0.997) \| N/A \| N/A \|  \|  \| \|  \| \| \| \| \| \| \| \| \| \| \| \| \|  \| \| **Kaplan - Meier survival analysis excluding patients with outcome prior to the time window** \| \| \| \| \| \| \| \| \| \| \|  \| \| \|  \| Cohort \| \| Patients in cohort \| Patients with outcome \| Median survival (days) \| Survival probability at end of time window \| \| \| \|  \| \| \|  \| 1 \| DM+AKD+DPP4i BMI < 30 1 \| 1,371 \| 254 \| -- \| 70.42% \| \| \| \|  \| \| \|  \| 2 \| DM+AKD without DPP4i BMI < 30 1 \| 1,377 \| 297 \| -- \| 65.86% \| \| \| \|  \| \| \| \| \| \| \| \| \| \| \| \| \|  \| \| \|  \|  \| \| χ^2^ \| df \| p \|  \|  \|  \| \|  \| \| \|  \| **Log-Rank Test** \| \| 7.628 \| 1 \| 0.006 \|  \|  \|  \| \|  \| \| \| \| \| \| \| \| \| \| \| \| \|  \| \| \|  \|  \| \| Hazard Ratio \| 95% CI \| χ^2^ \| df \| p \| \| \|  \| \| \|  \| **Hazard Ratio and Proportionality** \| \| 0.790 \| (0.668, 0.934) \| 3.330 \| 1 \| 0.068 \| \| \|  \| \| \| \| \| \| \| \| \| \| \| \| \|  \| \| **Number of instances excluding patients with outcome prior to the time window** \| \| \| \| \| \| \| \| \| \| \|  \| \| \|  \| Cohort \| \| Patients in cohort \| Patients with outcome \| Mean \| Standard Deviation \| Median \| \| \|  \| \| \|  \| 1 \| DM+AKD+DPP4i BMI < 30 1 \| 1,371 \| 254 \| 1.181 \| 0.443 \| 1 \| \| \|  \| \| \|  \| 2 \| DM+AKD without DPP4i BMI < 30 1 \| 1,377 \| 297 \| 1.229 \| 0.611 \| 1 \| \| \|  \| \| \| \| \| \| \| \| \| \| \| \| \|  \| \| \|  \|  \| \| t \| df \| p \|  \|  \|  \| \|  \| \| \|  \| **Test Statistics** \| \| -1.037 \| 549 \| 0.300 \|  \|  \|  \| \|  \| \| \| \| \| \| \| \| \| \| \| \| \| **2 MAKE** \| \| \| \| \| \| \| \| \| \| \| \| \|  \| \| **Risk analysis** \| \| \| \| \| \| \| \| \| \| \|  \|  \| \| \| Cohort \| \| Patients in cohort \| Patients with outcome \| Risk \| \| \| \| \|  \| \| \|  \| 1 \| DM+AKD+DPP4i BMI < 30 1 \| 1,482 \| 288 \| 0.194 \| \| \| \| \|  \| \| \|  \| 2 \| DM+AKD without DPP4i BMI < 30 1 \| 1,482 \| 333 \| 0.225 \| \| \| \| \|  \| \| \| \| \| \| \| \| \| \| \| \| \|  \| \| \|  \|  \| \|  \| 95% CI \| z \| p \|  \|  \| \|  \| \| \|  \| **Risk Difference** \| \| -0.030 \| (-0.060, -0.001) \| -2.031 \| 0.042 \|  \|  \| \|  \| \| \|  \| **Risk Ratio** \| \| 0.865 \| (0.752, 0.995) \| N/A \| N/A \|  \|  \| \|  \| \| \|  \| **Odds Ratio** \| \| 0.832 \| (0.697, 0.994) \| N/A \| N/A \|  \|  \| \|  \| \| \| \| \| \| \| \| \| \| \| \| \|  \| \| **Kaplan - Meier survival analysis** \| \| \| \| \| \| \| \| \| \| \|  \| \| \|  \| Cohort \| \| Patients in cohort \| Patients with outcome \| Median survival (days) \| Survival probability at end of time window \| \| \| \|  \| \| \|  \| 1 \| DM+AKD+DPP4i BMI < 30 1 \| 1,482 \| 288 \| -- \| 69.14% \| \| \| \|  \| \| \|  \| 2 \| DM+AKD without DPP4i BMI < 30 1 \| 1,482 \| 333 \| -- \| 64.50% \| \| \| \|  \| \| \| \| \| \| \| \| \| \| \| \| \|  \| \| \|  \|  \| \| χ^2^ \| df \| p \|  \|  \|  \| \|  \| \| \|  \| **Log-Rank Test** \| \| 7.029 \| 1 \| 0.008 \|  \|  \|  \| \|  \| \| \| \| \| \| \| \| \| \| \| \| \|  \| \| \|  \|  \| \| Hazard Ratio \| 95% CI \| χ^2^ \| df \| p \| \| \|  \| \| \|  \| **Hazard Ratio and Proportionality** \| \| 0.808 \| (0.690, 0.946) \| 1.656 \| 1 \| 0.198 \| \| \|  \| \| \| \| \| \| \| \| \| \| \| \| \| **3 MACE** \| \| \| \| \| \| \| \| \| \| \| \| \|  \| \| **Risk analysis excluding patients with outcome prior to the time window** \| \| \| \| \| \| \| \| \| \| \|  \|  \| \| \| Cohort \| \| Patients in cohort \| Patients with outcome \| Risk \| \| \| \| \|  \| \| \|  \| 1 \| DM+AKD+DPP4i BMI < 30 1 \| 968 \| 235 \| 0.243 \| \| \| \| \|  \| \| \|  \| 2 \| DM+AKD without DPP4i BMI < 30 1 \| 1,000 \| 278 \| 0.278 \| \| \| \| \|  \| \| \| \| \| \| \| \| \| \| \| \| \|  \| \| \|  \|  \| \|  \| 95% CI \| z \| p \|  \|  \| \|  \| \| \|  \| **Risk Difference** \| \| -0.035 \| (-0.074, 0.004) \| -1.780 \| 0.075 \|  \|  \| \|  \| \| \|  \| **Risk Ratio** \| \| 0.873 \| (0.752, 1.014) \| N/A \| N/A \|  \|  \| \|  \| \| \|  \| **Odds Ratio** \| \| 0.833 \| (0.680, 1.019) \| N/A \| N/A \|  \|  \| \|  \| \| \| \| \| \| \| \| \| \| \| \| \|  \| \| **Kaplan - Meier survival analysis excluding patients with outcome prior to the time window** \| \| \| \| \| \| \| \| \| \| \|  \| \| \|  \| Cohort \| \| Patients in cohort \| Patients with outcome \| Median survival (days) \| Survival probability at end of time window \| \| \| \|  \| \| \|  \| 1 \| DM+AKD+DPP4i BMI < 30 1 \| 968 \| 235 \| -- \| 62.89% \| \| \| \|  \| \| \|  \| 2 \| DM+AKD without DPP4i BMI < 30 1 \| 1,000 \| 278 \| -- \| 58.10% \| \| \| \|  \| \| \| \| \| \| \| \| \| \| \| \| \|  \| \| \|  \|  \| \| χ^2^ \| df \| p \|  \|  \|  \| \|  \| \| \|  \| **Log-Rank Test** \| \| 4.927 \| 1 \| 0.026 \|  \|  \|  \| \|  \| \| \| \| \| \| \| \| \| \| \| \| \|  \| \| \|  \|  \| \| Hazard Ratio \| 95% CI \| χ^2^ \| df \| p \| \| \|  \| \| \|  \| **Hazard Ratio and Proportionality** \| \| 0.822 \| (0.691, 0.978) \| 1.084 \| 1 \| 0.298 \| \| \|  \| \| \| \| \| \| \| \| \| \| \| \| \|  \| \| **Number of instances excluding patients with outcome prior to the time window** \| \| \| \| \| \| \| \| \| \| \|  \| \| \|  \| Cohort \| \| Patients in cohort \| Patients with outcome \| Mean \| Standard Deviation \| Median \| \| \|  \| \| \|  \| 1 \| DM+AKD+DPP4i BMI < 30 1 \| 968 \| 235 \| 4.460 \| 11.201 \| 1 \| \| \|  \| \| \|  \| 2 \| DM+AKD without DPP4i BMI < 30 1 \| 1,000 \| 278 \| 2.788 \| 6.419 \| 1 \| \| \|  \| \| \| \| \| \| \| \| \| \| \| \| \|  \| \| \|  \|  \| \| t \| df \| p \|  \|  \|  \| \|  \| \| \|  \| **Test Statistics** \| \| 2.112 \| 511 \| 0.035 \|  \|  \|  \|   **BMI > 30 kg/m^2^**   \| **1 Mortality** \| \| \| \| \| \| \| \| \| \| \| \| \| \| \| \| \| --- \| --- \| --- \| --- \| --- \| --- \| --- \| --- \| --- \| --- \| --- \| --- \| --- \| --- \| --- \| --- \| \| **Risk analysis excluding patients with outcome prior to the time window** \| \| \| \| \| \| \| \| \| \| \| \| \| \| \|  \| \| Cohort \| \| \| Patients in cohort \| \| Patients with outcome \| \| Risk \| \| \| \| \| \| \|  \|  \| \| 1 \| DM+AKD+DPP4i BMI > 30 1 \| \| 1,198 \| \| 182 \| \| 0.152 \| \| \| \| \| \| \|  \|  \| \| 2 \| DM+AKD without DPP4i BMI > 30 1 \| \| 1,188 \| \| 200 \| \| 0.168 \| \| \| \| \| \| \|  \| \| \| \| \| \| \| \| \| \| \| \| \| \| \| \| \|  \|  \| \|  \| \| \|  \| \| 95% CI \| \| z \| p \|  \|  \| \| \| \|  \|  \| \| **Risk Difference** \| \| \| -0.016 \| \| (-0.046, 0.013) \| \| -1.094 \| 0.274 \|  \|  \| \| \| \|  \|  \| \| **Risk Ratio** \| \| \| 0.902 \| \| (0.751, 1.085) \| \| N/A \| N/A \|  \|  \| \| \| \|  \|  \| \| **Odds Ratio** \| \| \| 0.885 \| \| (0.711, 1.102) \| \| N/A \| N/A \|  \|  \| \| \| \|  \| \| \| \| \| \| \| \| \| \| \| \| \| \| \| \| \| **Kaplan - Meier survival analysis excluding patients with outcome prior to the time window** \| \| \| \| \| \| \| \| \| \| \| \| \| \| \|  \|  \| \| Cohort \| \| \| Patients in cohort \| \| Patients with outcome \| \| Median survival (days) \| Survival probability at end of time window \| \| \| \| \| \|  \|  \| \| 1 \| DM+AKD+DPP4i BMI > 30 1 \| \| 1,198 \| \| 182 \| \| -- \| 77.65% \| \| \| \| \| \|  \|  \| \| 2 \| DM+AKD without DPP4i BMI > 30 1 \| \| 1,188 \| \| 200 \| \| -- \| 73.58% \| \| \| \| \| \|  \| \| \| \| \| \| \| \| \| \| \| \| \| \| \| \| \|  \|  \| \|  \| \| \| χ^2^ \| \| df \| \| p \|  \|  \|  \| \| \| \|  \|  \| \| **Log-Rank Test** \| \| \| 4.591 \| \| 1 \| \| 0.032 \|  \|  \|  \| \| \| \|  \| \| \| \| \| \| \| \| \| \| \| \| \| \| \| \| \|  \|  \| \|  \| \| \| Hazard Ratio \| \| 95% CI \| \| χ^2^ \| df \| p \| \| \| \| \|  \|  \| \| **Hazard Ratio and Proportionality** \| \| \| 0.803 \| \| (0.657, 0.982) \| \| 0.526 \| 1 \| 0.469 \| \| \| \| \|  \| \| \| \| \| \| \| \| \| \| \| \| \| \| \| \| \| **Number of instances excluding patients with outcome prior to the time window** \| \| \| \| \| \| \| \| \| \| \| \| \| \| \|  \|  \| \| Cohort \| \| \| Patients in cohort \| \| Patients with outcome \| \| Mean \| Standard Deviation \| Median \| \| \| \| \|  \|  \| \| 1 \| DM+AKD+DPP4i BMI > 30 1 \| \| 1,198 \| \| 182 \| \| 1.214 \| 0.411 \| 1 \| \| \| \| \|  \|  \| \| 2 \| DM+AKD without DPP4i BMI > 30 1 \| \| 1,188 \| \| 200 \| \| 1.210 \| 0.554 \| 1 \| \| \| \| \|  \| \| \| \| \| \| \| \| \| \| \| \| \| \| \| \| \|  \|  \| \|  \| \| \| t \| \| df \| \| p \|  \|  \|  \| \| \| \|  \|  \| \| **Test Statistics** \| \| \| 0.085 \| \| 380 \| \| 0.932 \|  \|  \|  \| \| \| \|  \| \| \| \| \| \| \| \| \| \| \| \| \| \| \| \| \| **2 MAKE** \| \| \| \| \| \| \| \| \| \| \| \| \| \| \| \| \| **Risk analysis** \| \| \| \| \| \| \| \| \| \| \| \| \| \| \|  \| \| Cohort \| \| \| Patients in cohort \| \| Patients with outcome \| \| Risk \| \| \| \| \| \| \|  \|  \| \| 1 \| DM+AKD+DPP4i BMI > 30 1 \| \| 1,275 \| \| 204 \| \| 0.160 \| \| \| \| \| \| \|  \|  \| \| 2 \| DM+AKD without DPP4i BMI > 30 1 \| \| 1,275 \| \| 228 \| \| 0.179 \| \| \| \| \| \| \|  \| \| \| \| \| \| \| \| \| \| \| \| \| \| \| \| \|  \|  \| \|  \| \| \|  \| \| 95% CI \| \| z \| p \|  \|  \| \| \| \|  \|  \| \| **Risk Difference** \| \| \| -0.019 \| \| (-0.048, 0.010) \| \| -1.267 \| 0.205 \|  \|  \| \| \| \|  \|  \| \| **Risk Ratio** \| \| \| 0.895 \| \| (0.753, 1.063) \| \| N/A \| N/A \|  \|  \| \| \| \|  \|  \| \| **Odds Ratio** \| \| \| 0.875 \| \| (0.711, 1.076) \| \| N/A \| N/A \|  \|  \| \| \| \|  \| \| \| \| \| \| \| \| \| \| \| \| \| \| \| \| \| **Kaplan - Meier survival analysis** \| \| \| \| \| \| \| \| \| \| \| \| \| \| \|  \|  \| \| Cohort \| \| \| Patients in cohort \| \| Patients with outcome \| \| Median survival (days) \| Survival probability at end of time window \| \| \| \| \| \|  \|  \| \| 1 \| DM+AKD+DPP4i BMI > 30 1 \| \| 1,275 \| \| 204 \| \| -- \| 76.57% \| \| \| \| \| \|  \|  \| \| 2 \| DM+AKD without DPP4i BMI > 30 1 \| \| 1,275 \| \| 228 \| \| -- \| 72.38% \| \| \| \| \| \|  \| \| \| \| \| \| \| \| \| \| \| \| \| \| \| \| \|  \|  \| \|  \| \| \| χ^2^ \| \| df \| \| p \|  \|  \|  \| \| \| \|  \|  \| \| **Log-Rank Test** \| \| \| 5.643 \| \| 1 \| \| 0.018 \|  \|  \|  \| \| \| \|  \| \| \| \| \| \| \| \| \| \| \| \| \| \| \| \| \|  \|  \| \|  \| \| \| Hazard Ratio \| \| 95% CI \| \| χ^2^ \| df \| p \| \| \| \| \|  \|  \| \| **Hazard Ratio and Proportionality** \| \| \| 0.796 \| \| (0.659, 0.961) \| \| 1.373 \| 1 \| 0.241 \| \| \| \| \|  \| \| \| \| \| \| \| \| \| \| \| \| \| \| \| \| \| **3 MACE** \| \| \| \| \| \| \| \| \| \| \| \| \| \| \| \| \| **Risk analysis excluding patients with outcome prior to the time window** \| \| \| \| \| \| \| \| \| \| \| \| \| \| \|  \| \| Cohort \| \| \| Patients in cohort \| \| Patients with outcome \| \| Risk \| \| \| \| \| \| \|  \|  \| \| 1 \| DM+AKD+DPP4i BMI > 30 1 \| \| 896 \| \| 198 \| \| 0.221 \| \| \| \| \| \| \|  \|  \| \| 2 \| DM+AKD without DPP4i BMI > 30 1 \| \| 924 \| \| 198 \| \| 0.214 \| \| \| \| \| \| \|  \| \| \| \| \| \| \| \| \| \| \| \| \| \| \| \| \|  \|  \| \|  \| \| \|  \| \| 95% CI \| \| z \| p \|  \|  \| \| \| \|  \|  \| \| **Risk Difference** \| \| \| 0.007 \| \| (-0.031, 0.045) \| \| 0.346 \| 0.729 \|  \|  \| \| \| \|  \|  \| \| **Risk Ratio** \| \| \| 1.031 \| \| (0.866, 1.228) \| \| N/A \| N/A \|  \|  \| \| \| \|  \|  \| \| **Odds Ratio** \| \| \| 1.040 \| \| (0.832, 1.300) \| \| N/A \| N/A \|  \|  \| \| \| \|  \| \| \| \| \| \| \| \| \| \| \| \| \| \| \| \| \| **Kaplan - Meier survival analysis excluding patients with outcome prior to the time window** \| \| \| \| \| \| \| \| \| \| \| \| \| \| \|  \|  \| \| Cohort \| \| \| Patients in cohort \| \| Patients with outcome \| \| Median survival (days) \| Survival probability at end of time window \| \| \| \| \| \|  \|  \| \| 1 \| DM+AKD+DPP4i BMI > 30 1 \| \| 896 \| \| 198 \| \| -- \| 68.87% \| \| \| \| \| \|  \|  \| \| 2 \| DM+AKD without DPP4i BMI > 30 1 \| \| 924 \| \| 198 \| \| -- \| 67.25% \| \| \| \| \| \|  \| \| \| \| \| \| \| \| \| \| \| \| \| \| \| \| \|  \|  \| \|  \| \| \| χ^2^ \| \| df \| \| p \|  \|  \|  \| \| \| \|  \|  \| \| **Log-Rank Test** \| \| \| 0.419 \| \| 1 \| \| 0.517 \|  \|  \|  \| \| \| \|  \| \| \| \| \| \| \| \| \| \| \| \| \| \| \| \| \|  \|  \| \|  \| \| \| Hazard Ratio \| \| 95% CI \| \| χ^2^ \| df \| p \| \| \| \| \|  \|  \| \| **Hazard Ratio and Proportionality** \| \| \| 0.937 \| \| (0.769, 1.141) \| \| 0.002 \| 1 \| 0.962 \| \| \| \| \|  \| \| \| \| \| \| \| \| \| \| \| \| \| \| \| \| \| **Number of instances excluding patients with outcome prior to the time window** \| \| \| \| \| \| \| \| \| \| \| \| \| \| \|  \|  \| \| Cohort \| \| \| Patients in cohort \| \| Patients with outcome \| \| Mean \| Standard Deviation \| Median \| \| \| \| \|  \|  \| \| 1 \| DM+AKD+DPP4i BMI > 30 1 \| \| 896 \| \| 198 \| \| 4.495 \| 9.887 \| 1 \| \| \| \| \|  \|  \| \| 2 \| DM+AKD without DPP4i BMI > 30 1 \| \| 924 \| \| 198 \| \| 3.288 \| 5.870 \| 1 \| \| \| \| \|  \| \| \| \| \| \| \| \| \| \| \| \| \| \| \| \| \|  \|  \| \|  \| \| \| t \| \| df \| \| p \|  \|  \|  \| \| \| \|  \|  \| \| **Test Statistics** \| \| \| 1.477 \| \| 394 \| \| 0.140 \|  \|  \|  \| \| \|   **ACEi/ARB concurrent usage (ACEi, NLM:VA:CV800;** **ARB, NLM:VA:CV805 )**   \| **1 Mortality** \| \| \| \| \| \| \| \| \| \| \| \| \| --- \| --- \| --- \| --- \| --- \| --- \| --- \| --- \| --- \| --- \| --- \| --- \| \|  \| \| **Risk analysis excluding patients with outcome prior to the time window** \| \| \| \| \| \| \| \| \| \| \|  \|  \| \| \| Cohort \| \| Patients in cohort \| Patients with outcome \| Risk \| \| \| \| \|  \| \| \|  \| 1 \| DM+AKD+DPP4i with ACEi/ARB 1 \| 3,453 \| 519 \| 0.150 \| \| \| \| \|  \| \| \|  \| 2 \| DM+AKD without DPP4i with ACEi/ARB 1 \| 3,490 \| 573 \| 0.164 \| \| \| \| \|  \| \| \| \| \| \| \| \| \| \| \| \| \|  \| \| \|  \|  \| \|  \| 95% CI \| z \| p \|  \|  \| \|  \| \| \|  \| **Risk Difference** \| \| -0.014 \| (-0.031, 0.003) \| -1.588 \| 0.112 \|  \|  \| \|  \| \| \|  \| **Risk Ratio** \| \| 0.915 \| (0.821, 1.021) \| N/A \| N/A \|  \|  \| \|  \| \| \|  \| **Odds Ratio** \| \| 0.901 \| (0.791, 1.025) \| N/A \| N/A \|  \|  \| \|  \| \| \| \| \| \| \| \| \| \| \| \| \|  \| \| **Kaplan - Meier survival analysis excluding patients with outcome prior to the time window** \| \| \| \| \| \| \| \| \| \| \|  \| \| \|  \| Cohort \| \| Patients in cohort \| Patients with outcome \| Median survival (days) \| Survival probability at end of time window \| \| \| \|  \| \| \|  \| 1 \| DM+AKD+DPP4i with ACEi/ARB 1 \| 3,453 \| 519 \| -- \| 76.23% \| \| \| \|  \| \| \|  \| 2 \| DM+AKD without DPP4i with ACEi/ARB 1 \| 3,490 \| 573 \| -- \| 73.96% \| \| \| \|  \| \| \| \| \| \| \| \| \| \| \| \| \|  \| \| \|  \|  \| \| χ^2^ \| df \| p \|  \|  \|  \| \|  \| \| \|  \| **Log-Rank Test** \| \| 4.542 \| 1 \| 0.033 \|  \|  \|  \| \|  \| \| \| \| \| \| \| \| \| \| \| \| \|  \| \| \|  \|  \| \| Hazard Ratio \| 95% CI \| χ^2^ \| df \| p \| \| \|  \| \| \|  \| **Hazard Ratio and Proportionality** \| \| 0.879 \| (0.781, 0.990) \| 1.263 \| 1 \| 0.261 \| \| \|  \| \| \| \| \| \| \| \| \| \| \| \| \|  \| \| **Number of instances excluding patients with outcome prior to the time window** \| \| \| \| \| \| \| \| \| \| \|  \| \| \|  \| Cohort \| \| Patients in cohort \| Patients with outcome \| Mean \| Standard Deviation \| Median \| \| \|  \| \| \|  \| 1 \| DM+AKD+DPP4i with ACEi/ARB 1 \| 3,453 \| 519 \| 1.222 \| 0.476 \| 1 \| \| \|  \| \| \|  \| 2 \| DM+AKD without DPP4i with ACEi/ARB 1 \| 3,490 \| 573 \| 1.253 \| 0.546 \| 1 \| \| \|  \| \| \| \| \| \| \| \| \| \| \| \| \|  \| \| \|  \|  \| \| t \| df \| p \|  \|  \|  \| \|  \| \| \|  \| **Test Statistics** \| \| -1.011 \| 1090 \| 0.312 \|  \|  \|  \| \|  \| \| \| \| \| \| \| \| \| \| \| \| \| **2 MAKE** \| \| \| \| \| \| \| \| \| \| \| \| \|  \| \| **Risk analysis** \| \| \| \| \| \| \| \| \| \| \|  \|  \| \| \| Cohort \| \| Patients in cohort \| Patients with outcome \| Risk \| \| \| \| \|  \| \| \|  \| 1 \| DM+AKD+DPP4i with ACEi/ARB 1 \| 3,639 \| 582 \| 0.160 \| \| \| \| \|  \| \| \|  \| 2 \| DM+AKD without DPP4i with ACEi/ARB 1 \| 3,639 \| 639 \| 0.176 \| \| \| \| \|  \| \| \| \| \| \| \| \| \| \| \| \| \|  \| \| \|  \|  \| \|  \| 95% CI \| z \| p \|  \|  \| \|  \| \| \|  \| **Risk Difference** \| \| -0.016 \| (-0.033, 0.002) \| -1.788 \| 0.074 \|  \|  \| \|  \| \| \|  \| **Risk Ratio** \| \| 0.911 \| (0.822, 1.009) \| N/A \| N/A \|  \|  \| \|  \| \| \|  \| **Odds Ratio** \| \| 0.894 \| (0.790, 1.011) \| N/A \| N/A \|  \|  \| \|  \| \| \| \| \| \| \| \| \| \| \| \| \|  \| \| **Kaplan - Meier survival analysis** \| \| \| \| \| \| \| \| \| \| \|  \| \| \|  \| Cohort \| \| Patients in cohort \| Patients with outcome \| Median survival (days) \| Survival probability at end of time window \| \| \| \|  \| \| \|  \| 1 \| DM+AKD+DPP4i with ACEi/ARB 1 \| 3,639 \| 582 \| -- \| 74.67% \| \| \| \|  \| \| \|  \| 2 \| DM+AKD without DPP4i with ACEi/ARB 1 \| 3,639 \| 639 \| -- \| 72.09% \| \| \| \|  \| \| \| \| \| \| \| \| \| \| \| \| \|  \| \| \|  \|  \| \| χ^2^ \| df \| p \|  \|  \|  \| \|  \| \| \|  \| **Log-Rank Test** \| \| 5.800 \| 1 \| 0.016 \|  \|  \|  \| \|  \| \| \| \| \| \| \| \| \| \| \| \| \|  \| \| \|  \|  \| \| Hazard Ratio \| 95% CI \| χ^2^ \| df \| p \| \| \|  \| \| \|  \| **Hazard Ratio and Proportionality** \| \| 0.871 \| (0.779, 0.975) \| 1.601 \| 1 \| 0.206 \| \| \|  \| \| \| \| \| \| \| \| \| \| \| \| \| **3 MACE** \| \| \| \| \| \| \| \| \| \| \| \| \|  \| \| **Risk analysis excluding patients with outcome prior to the time window** \| \| \| \| \| \| \| \| \| \| \|  \|  \| \| \| Cohort \| \| Patients in cohort \| Patients with outcome \| Risk \| \| \| \| \|  \| \| \|  \| 1 \| DM+AKD+DPP4i with ACEi/ARB 1 \| 2,322 \| 520 \| 0.224 \| \| \| \| \|  \| \| \|  \| 2 \| DM+AKD without DPP4i with ACEi/ARB 1 \| 2,331 \| 549 \| 0.236 \| \| \| \| \|  \| \| \| \| \| \| \| \| \| \| \| \| \|  \| \| \|  \|  \| \|  \| 95% CI \| z \| p \|  \|  \| \|  \| \| \|  \| **Risk Difference** \| \| -0.012 \| (-0.036, 0.013) \| -0.939 \| 0.348 \|  \|  \| \|  \| \| \|  \| **Risk Ratio** \| \| 0.951 \| (0.856, 1.056) \| N/A \| N/A \|  \|  \| \|  \| \| \|  \| **Odds Ratio** \| \| 0.937 \| (0.817, 1.074) \| N/A \| N/A \|  \|  \| \|  \| \| \| \| \| \| \| \| \| \| \| \| \|  \| \| **Kaplan - Meier survival analysis excluding patients with outcome prior to the time window** \| \| \| \| \| \| \| \| \| \| \|  \| \| \|  \| Cohort \| \| Patients in cohort \| Patients with outcome \| Median survival (days) \| Survival probability at end of time window \| \| \| \|  \| \| \|  \| 1 \| DM+AKD+DPP4i with ACEi/ARB 1 \| 2,322 \| 520 \| -- \| 65.78% \| \| \| \|  \| \| \|  \| 2 \| DM+AKD without DPP4i with ACEi/ARB 1 \| 2,331 \| 549 \| -- \| 64.00% \| \| \| \|  \| \| \| \| \| \| \| \| \| \| \| \| \|  \| \| \|  \|  \| \| χ^2^ \| df \| p \|  \|  \|  \| \|  \| \| \|  \| **Log-Rank Test** \| \| 3.020 \| 1 \| 0.082 \|  \|  \|  \| \|  \| \| \| \| \| \| \| \| \| \| \| \| \|  \| \| \|  \|  \| \| Hazard Ratio \| 95% CI \| χ^2^ \| df \| p \| \| \|  \| \| \|  \| **Hazard Ratio and Proportionality** \| \| 0.899 \| (0.798, 1.014) \| 3.864 \| 1 \| 0.049 \| \| \|  \| \| \| \| \| \| \| \| \| \| \| \| \|  \| \| **Number of instances excluding patients with outcome prior to the time window** \| \| \| \| \| \| \| \| \| \| \|  \| \| \|  \| Cohort \| \| Patients in cohort \| Patients with outcome \| Mean \| Standard Deviation \| Median \| \| \|  \| \| \|  \| 1 \| DM+AKD+DPP4i with ACEi/ARB 1 \| 2,322 \| 520 \| 3.688 \| 9.020 \| 1 \| \| \|  \| \| \|  \| 2 \| DM+AKD without DPP4i with ACEi/ARB 1 \| 2,331 \| 549 \| 2.787 \| 4.962 \| 1 \| \| \|  \| \| \| \| \| \| \| \| \| \| \| \| \|  \| \| \|  \|  \| \| t \| df \| p \|  \|  \|  \| \|  \| \| \|  \| **Test Statistics** \| \| 2.039 \| 1067 \| 0.042 \|  \|  \|  \| \|  \| \| \| \| \| \| \| \| \| \| \| \|   **Without ACEi/ARB concurrent usage**   \| **1 Mortality** \| \| \| \| \| \| \| \| \| \| \| \| \| \| \| \| \| --- \| --- \| --- \| --- \| --- \| --- \| --- \| --- \| --- \| --- \| --- \| --- \| --- \| --- \| --- \| --- \| \| **Risk analysis excluding patients with outcome prior to the time window** \| \| \| \| \| \| \| \| \| \| \| \| \| \| \|  \| \| Cohort \| \| \| Patients in cohort \| \| Patients with outcome \| \| Risk \| \| \| \| \| \| \|  \|  \| \| 1 \| DM+AKD + DPP4i without ACEi/ARB 1 \| \| 2,750 \| \| 413 \| \| 0.150 \| \| \| \| \| \| \|  \|  \| \| 2 \| DM+AKD without DPP4i without ACEi/ARB 1 \| \| 2,743 \| \| 461 \| \| 0.168 \| \| \| \| \| \| \|  \| \| \| \| \| \| \| \| \| \| \| \| \| \| \| \| \|  \|  \| \|  \| \| \|  \| \| 95% CI \| \| z \| p \|  \|  \| \| \| \|  \|  \| \| **Risk Difference** \| \| \| -0.018 \| \| (-0.037, 0.001) \| \| -1.812 \| 0.070 \|  \|  \| \| \| \|  \|  \| \| **Risk Ratio** \| \| \| 0.894 \| \| (0.791, 1.009) \| \| N/A \| N/A \|  \|  \| \| \| \|  \|  \| \| **Odds Ratio** \| \| \| 0.875 \| \| (0.757, 1.011) \| \| N/A \| N/A \|  \|  \| \| \| \|  \| \| \| \| \| \| \| \| \| \| \| \| \| \| \| \| \| **Kaplan - Meier survival analysis excluding patients with outcome prior to the time window** \| \| \| \| \| \| \| \| \| \| \| \| \| \| \|  \|  \| \| Cohort \| \| \| Patients in cohort \| \| Patients with outcome \| \| Median survival (days) \| Survival probability at end of time window \| \| \| \| \| \|  \|  \| \| 1 \| DM+AKD + DPP4i without ACEi/ARB 1 \| \| 2,750 \| \| 413 \| \| -- \| 72.51% \| \| \| \| \| \|  \|  \| \| 2 \| DM+AKD without DPP4i without ACEi/ARB 1 \| \| 2,743 \| \| 461 \| \| -- \| 68.17% \| \| \| \| \| \|  \| \| \| \| \| \| \| \| \| \| \| \| \| \| \| \| \|  \|  \| \|  \| \| \| χ^2^ \| \| df \| \| p \|  \|  \|  \| \| \| \|  \|  \| \| **Log-Rank Test** \| \| \| 9.330 \| \| 1 \| \| 0.002 \|  \|  \|  \| \| \| \|  \| \| \| \| \| \| \| \| \| \| \| \| \| \| \| \| \|  \|  \| \|  \| \| \| Hazard Ratio \| \| 95% CI \| \| χ^2^ \| df \| p \| \| \| \| \|  \|  \| \| **Hazard Ratio and Proportionality** \| \| \| 0.813 \| \| (0.712, 0.929) \| \| 1.217 \| 1 \| 0.270 \| \| \| \| \|  \| \| \| \| \| \| \| \| \| \| \| \| \| \| \| \| \| **Number of instances excluding patients with outcome prior to the time window** \| \| \| \| \| \| \| \| \| \| \| \| \| \| \|  \|  \| \| Cohort \| \| \| Patients in cohort \| \| Patients with outcome \| \| Mean \| Standard Deviation \| Median \| \| \| \| \|  \|  \| \| 1 \| DM+AKD + DPP4i without ACEi/ARB 1 \| \| 2,750 \| \| 413 \| \| 1.213 \| 0.564 \| 1 \| \| \| \| \|  \|  \| \| 2 \| DM+AKD without DPP4i without ACEi/ARB 1 \| \| 2,743 \| \| 461 \| \| 1.171 \| 0.416 \| 1 \| \| \| \| \|  \| \| \| \| \| \| \| \| \| \| \| \| \| \| \| \| \|  \|  \| \|  \| \| \| t \| \| df \| \| p \|  \|  \|  \| \| \| \|  \|  \| \| **Test Statistics** \| \| \| 1.252 \| \| 872 \| \| 0.211 \|  \|  \|  \| \| \| \|  \| \| \| \| \| \| \| \| \| \| \| \| \| \| \| \| \| **2 MAKE** \| \| \| \| \| \| \| \| \| \| \| \| \| \| \| \| \| **Risk analysis** \| \| \| \| \| \| \| \| \| \| \| \| \| \| \|  \| \| Cohort \| \| \| Patients in cohort \| \| Patients with outcome \| \| Risk \| \| \| \| \| \| \|  \|  \| \| 1 \| DM+AKD + DPP4i without ACEi/ARB 1 \| \| 2,904 \| \| 463 \| \| 0.159 \| \| \| \| \| \| \|  \|  \| \| 2 \| DM+AKD without DPP4i without ACEi/ARB 1 \| \| 2,904 \| \| 493 \| \| 0.170 \| \| \| \| \| \| \|  \| \| \| \| \| \| \| \| \| \| \| \| \| \| \| \| \|  \|  \| \|  \| \| \|  \| \| 95% CI \| \| z \| p \|  \|  \| \| \| \|  \|  \| \| **Risk Difference** \| \| \| -0.010 \| \| (-0.029, 0.009) \| \| -1.062 \| 0.288 \|  \|  \| \| \| \|  \|  \| \| **Risk Ratio** \| \| \| 0.939 \| \| (0.836, 1.055) \| \| N/A \| N/A \|  \|  \| \| \| \|  \|  \| \| **Odds Ratio** \| \| \| 0.928 \| \| (0.807, 1.066) \| \| N/A \| N/A \|  \|  \| \| \| \|  \| \| \| \| \| \| \| \| \| \| \| \| \| \| \| \| \| **Kaplan - Meier survival analysis** \| \| \| \| \| \| \| \| \| \| \| \| \| \| \|  \|  \| \| Cohort \| \| \| Patients in cohort \| \| Patients with outcome \| \| Median survival (days) \| Survival probability at end of time window \| \| \| \| \| \|  \|  \| \| 1 \| DM+AKD + DPP4i without ACEi/ARB 1 \| \| 2,904 \| \| 463 \| \| -- \| 70.98% \| \| \| \| \| \|  \|  \| \| 2 \| DM+AKD without DPP4i without ACEi/ARB 1 \| \| 2,904 \| \| 493 \| \| -- \| 67.38% \| \| \| \| \| \|  \| \| \| \| \| \| \| \| \| \| \| \| \| \| \| \| \|  \|  \| \|  \| \| \| χ^2^ \| \| df \| \| p \|  \|  \|  \| \| \| \|  \|  \| \| **Log-Rank Test** \| \| \| 7.567 \| \| 1 \| \| 0.006 \|  \|  \|  \| \| \| \|  \| \| \| \| \| \| \| \| \| \| \| \| \| \| \| \| \|  \|  \| \|  \| \| \| Hazard Ratio \| \| 95% CI \| \| χ^2^ \| df \| p \| \| \| \| \|  \|  \| \| **Hazard Ratio and Proportionality** \| \| \| 0.837 \| \| (0.737, 0.950) \| \| 1.620 \| 1 \| 0.203 \| \| \| \| \|  \| \| \| \| \| \| \| \| \| \| \| \| \| \| \| \| \| **3 MACE** \| \| \| \| \| \| \| \| \| \| \| \| \| \| \| \| \| **Risk analysis excluding patients with outcome prior to the time window** \| \| \| \| \| \| \| \| \| \| \| \| \| \| \|  \| \| Cohort \| \| \| Patients in cohort \| \| Patients with outcome \| \| Risk \| \| \| \| \| \| \|  \|  \| \| 1 \| DM+AKD + DPP4i without ACEi/ARB 1 \| \| 2,138 \| \| 392 \| \| 0.183 \| \| \| \| \| \| \|  \|  \| \| 2 \| DM+AKD without DPP4i without ACEi/ARB 1 \| \| 2,197 \| \| 436 \| \| 0.198 \| \| \| \| \| \| \|  \| \| \| \| \| \| \| \| \| \| \| \| \| \| \| \| \|  \|  \| \|  \| \| \|  \| \| 95% CI \| \| z \| p \|  \|  \| \| \| \|  \|  \| \| **Risk Difference** \| \| \| -0.015 \| \| (-0.038, 0.008) \| \| -1.265 \| 0.206 \|  \|  \| \| \| \|  \|  \| \| **Risk Ratio** \| \| \| 0.924 \| \| (0.817, 1.045) \| \| N/A \| N/A \|  \|  \| \| \| \|  \|  \| \| **Odds Ratio** \| \| \| 0.907 \| \| (0.779, 1.055) \| \| N/A \| N/A \|  \|  \| \| \| \|  \| \| \| \| \| \| \| \| \| \| \| \| \| \| \| \| \| **Kaplan - Meier survival analysis excluding patients with outcome prior to the time window** \| \| \| \| \| \| \| \| \| \| \| \| \| \| \|  \|  \| \| Cohort \| \| \| Patients in cohort \| \| Patients with outcome \| \| Median survival (days) \| Survival probability at end of time window \| \| \| \| \| \|  \|  \| \| 1 \| DM+AKD + DPP4i without ACEi/ARB 1 \| \| 2,138 \| \| 392 \| \| -- \| 67.02% \| \| \| \| \| \|  \|  \| \| 2 \| DM+AKD without DPP4i without ACEi/ARB 1 \| \| 2,197 \| \| 436 \| \| -- \| 64.34% \| \| \| \| \| \|  \| \| \| \| \| \| \| \| \| \| \| \| \| \| \| \| \|  \|  \| \|  \| \| \| χ^2^ \| \| df \| \| p \|  \|  \|  \| \| \| \|  \|  \| \| **Log-Rank Test** \| \| \| 6.604 \| \| 1 \| \| 0.010 \|  \|  \|  \| \| \| \|  \| \| \| \| \| \| \| \| \| \| \| \| \| \| \| \| \|  \|  \| \|  \| \| \| Hazard Ratio \| \| 95% CI \| \| χ^2^ \| df \| p \| \| \| \| \|  \|  \| \| **Hazard Ratio and Proportionality** \| \| \| 0.836 \| \| (0.730, 0.959) \| \| 5.767 \| 1 \| 0.016 \| \| \| \| \|  \| \| \| \| \| \| \| \| \| \| \| \| \| \| \| \| \| **Number of instances excluding patients with outcome prior to the time window** \| \| \| \| \| \| \| \| \| \| \| \| \| \| \|  \|  \| \| Cohort \| \| \| Patients in cohort \| \| Patients with outcome \| \| Mean \| Standard Deviation \| Median \| \| \| \| \|  \|  \| \| 1 \| DM+AKD + DPP4i without ACEi/ARB 1 \| \| 2,138 \| \| 392 \| \| 2.314 \| 4.708 \| 1 \| \| \| \| \|  \|  \| \| 2 \| DM+AKD without DPP4i without ACEi/ARB 1 \| \| 2,197 \| \| 436 \| \| 2.167 \| 3.922 \| 1 \| \| \| \| \|  \| \| \| \| \| \| \| \| \| \| \| \| \| \| \| \| \|  \|  \| \|  \| \| \| t \| \| df \| \| p \|  \|  \|  \| \| \| \|  \|  \| \| **Test Statistics** \| \| \| 0.488 \| \| 826 \| \| 0.626 \|  \|  \|  \| \| \| \|  \| \| \| \| \| \| \| \| \| \| \| \| \| \| \| \|   **ACEi concurrent usage only**   \| **1 Mortality** \| \| \| \| \| \| \| \| \| \| \| \| \| \| \| \| \| --- \| --- \| --- \| --- \| --- \| --- \| --- \| --- \| --- \| --- \| --- \| --- \| --- \| --- \| --- \| --- \| \| **Risk analysis excluding patients with outcome prior to the time window** \| \| \| \| \| \| \| \| \| \| \| \| \| \| \|  \| \| Cohort \| \| \| Patients in cohort \| \| Patients with outcome \| \| Risk \| \| \| \| \| \| \|  \|  \| \| 1 \| DM+AKD+DPP4i with ACEi without ARB 1 \| \| 2,116 \| \| 334 \| \| 0.158 \| \| \| \| \| \| \|  \|  \| \| 2 \| DM+AKD without DPP4i with ACEi without ARB 1 \| \| 2,120 \| \| 383 \| \| 0.181 \| \| \| \| \| \| \|  \| \| \| \| \| \| \| \| \| \| \| \| \| \| \| \| \|  \|  \| \|  \| \| \|  \| \| 95% CI \| \| z \| p \|  \|  \| \| \| \|  \|  \| \| **Risk Difference** \| \| \| -0.023 \| \| (-0.045, -0.000) \| \| -1.980 \| 0.048 \|  \|  \| \| \| \|  \|  \| \| **Risk Ratio** \| \| \| 0.874 \| \| (0.764, 0.999) \| \| N/A \| N/A \|  \|  \| \| \| \|  \|  \| \| **Odds Ratio** \| \| \| 0.850 \| \| (0.724, 0.998) \| \| N/A \| N/A \|  \|  \| \| \| \|  \| \| \| \| \| \| \| \| \| \| \| \| \| \| \| \| \| **Kaplan - Meier survival analysis excluding patients with outcome prior to the time window** \| \| \| \| \| \| \| \| \| \| \| \| \| \| \|  \|  \| \| Cohort \| \| \| Patients in cohort \| \| Patients with outcome \| \| Median survival (days) \| Survival probability at end of time window \| \| \| \| \| \|  \|  \| \| 1 \| DM+AKD+DPP4i with ACEi without ARB 1 \| \| 2,116 \| \| 334 \| \| -- \| 75.59% \| \| \| \| \| \|  \|  \| \| 2 \| DM+AKD without DPP4i with ACEi without ARB 1 \| \| 2,120 \| \| 383 \| \| -- \| 71.57% \| \| \| \| \| \|  \| \| \| \| \| \| \| \| \| \| \| \| \| \| \| \| \|  \|  \| \|  \| \| \| χ^2^ \| \| df \| \| p \|  \|  \|  \| \| \| \|  \|  \| \| **Log-Rank Test** \| \| \| 5.039 \| \| 1 \| \| 0.025 \|  \|  \|  \| \| \| \|  \| \| \| \| \| \| \| \| \| \| \| \| \| \| \| \| \|  \|  \| \|  \| \| \| Hazard Ratio \| \| 95% CI \| \| χ^2^ \| df \| p \| \| \| \| \|  \|  \| \| **Hazard Ratio and Proportionality** \| \| \| 0.845 \| \| (0.730, 0.979) \| \| 0.082 \| 1 \| 0.775 \| \| \| \| \|  \| \| \| \| \| \| \| \| \| \| \| \| \| \| \| \| \| **Number of instances excluding patients with outcome prior to the time window** \| \| \| \| \| \| \| \| \| \| \| \| \| \| \|  \|  \| \| Cohort \| \| \| Patients in cohort \| \| Patients with outcome \| \| Mean \| Standard Deviation \| Median \| \| \| \| \|  \|  \| \| 1 \| DM+AKD+DPP4i with ACEi without ARB 1 \| \| 2,116 \| \| 334 \| \| 1.177 \| 0.412 \| 1 \| \| \| \| \|  \|  \| \| 2 \| DM+AKD without DPP4i with ACEi without ARB 1 \| \| 2,120 \| \| 383 \| \| 1.232 \| 0.698 \| 1 \| \| \| \| \|  \| \| \| \| \| \| \| \| \| \| \| \| \| \| \| \| \|  \|  \| \|  \| \| \| t \| \| df \| \| p \|  \|  \|  \| \| \| \|  \|  \| \| **Test Statistics** \| \| \| -1.277 \| \| 715 \| \| 0.202 \|  \|  \|  \| \| \| \|  \| \| \| \| \| \| \| \| \| \| \| \| \| \| \| \| \| **2 MAKE** \| \| \| \| \| \| \| \| \| \| \| \| \| \| \| \| \| **Risk analysis** \| \| \| \| \| \| \| \| \| \| \| \| \| \| \|  \| \| Cohort \| \| \| Patients in cohort \| \| Patients with outcome \| \| Risk \| \| \| \| \| \| \|  \|  \| \| 1 \| DM+AKD+DPP4i with ACEi without ARB 1 \| \| 2,210 \| \| 368 \| \| 0.167 \| \| \| \| \| \| \|  \|  \| \| 2 \| DM+AKD without DPP4i with ACEi without ARB 1 \| \| 2,210 \| \| 420 \| \| 0.190 \| \| \| \| \| \| \|  \| \| \| \| \| \| \| \| \| \| \| \| \| \| \| \| \|  \|  \| \|  \| \| \|  \| \| 95% CI \| \| z \| p \|  \|  \| \| \| \|  \|  \| \| **Risk Difference** \| \| \| -0.024 \| \| (-0.046, -0.001) \| \| -2.044 \| 0.041 \|  \|  \| \| \| \|  \|  \| \| **Risk Ratio** \| \| \| 0.876 \| \| (0.772, 0.995) \| \| N/A \| N/A \|  \|  \| \| \| \|  \|  \| \| **Odds Ratio** \| \| \| 0.851 \| \| (0.730, 0.994) \| \| N/A \| N/A \|  \|  \| \| \| \|  \| \| \| \| \| \| \| \| \| \| \| \| \| \| \| \| \| **Kaplan - Meier survival analysis** \| \| \| \| \| \| \| \| \| \| \| \| \| \| \|  \|  \| \| Cohort \| \| \| Patients in cohort \| \| Patients with outcome \| \| Median survival (days) \| Survival probability at end of time window \| \| \| \| \| \|  \|  \| \| 1 \| DM+AKD+DPP4i with ACEi without ARB 1 \| \| 2,210 \| \| 368 \| \| -- \| 74.18% \| \| \| \| \| \|  \|  \| \| 2 \| DM+AKD without DPP4i with ACEi without ARB 1 \| \| 2,210 \| \| 420 \| \| -- \| 70.13% \| \| \| \| \| \|  \| \| \| \| \| \| \| \| \| \| \| \| \| \| \| \| \|  \|  \| \|  \| \| \| χ^2^ \| \| df \| \| p \|  \|  \|  \| \| \| \|  \|  \| \| **Log-Rank Test** \| \| \| 5.017 \| \| 1 \| \| 0.025 \|  \|  \|  \| \| \| \|  \| \| \| \| \| \| \| \| \| \| \| \| \| \| \| \| \|  \|  \| \|  \| \| \| Hazard Ratio \| \| 95% CI \| \| χ^2^ \| df \| p \| \| \| \| \|  \|  \| \| **Hazard Ratio and Proportionality** \| \| \| 0.852 \| \| (0.741, 0.980) \| \| 0.147 \| 1 \| 0.701 \| \| \| \| \| **3 MACE** \| \| \| \| \| \| \| \| \| \| \| \| \| \| \| \| \| **Risk analysis excluding patients with outcome prior to the time window** \| \| \| \| \| \| \| \| \| \| \| \| \| \| \|  \| \| Cohort \| \| \| Patients in cohort \| \| Patients with outcome \| \| Risk \| \| \| \| \| \| \|  \|  \| \| 1 \| DM+AKD+DPP4i with ACEi without ARB 1 \| \| 1,409 \| \| 322 \| \| 0.229 \| \| \| \| \| \| \|  \|  \| \| 2 \| DM+AKD without DPP4i with ACEi without ARB 1 \| \| 1,465 \| \| 348 \| \| 0.238 \| \| \| \| \| \| \|  \| \| \| \| \| \| \| \| \| \| \| \| \| \| \| \| \|  \|  \| \|  \| \| \|  \| \| 95% CI \| \| z \| p \|  \|  \| \| \| \|  \|  \| \| **Risk Difference** \| \| \| -0.009 \| \| (-0.040, 0.022) \| \| -0.571 \| 0.568 \|  \|  \| \| \| \|  \|  \| \| **Risk Ratio** \| \| \| 0.962 \| \| (0.842, 1.099) \| \| N/A \| N/A \|  \|  \| \| \| \|  \|  \| \| **Odds Ratio** \| \| \| 0.951 \| \| (0.800, 1.130) \| \| N/A \| N/A \|  \|  \| \| \| \|  \| \| \| \| \| \| \| \| \| \| \| \| \| \| \| \| \| **Kaplan - Meier survival analysis excluding patients with outcome prior to the time window** \| \| \| \| \| \| \| \| \| \| \| \| \| \| \|  \|  \| \| Cohort \| \| \| Patients in cohort \| \| Patients with outcome \| \| Median survival (days) \| Survival probability at end of time window \| \| \| \| \| \|  \|  \| \| 1 \| DM+AKD+DPP4i with ACEi without ARB 1 \| \| 1,409 \| \| 322 \| \| -- \| 65.82% \| \| \| \| \| \|  \|  \| \| 2 \| DM+AKD without DPP4i with ACEi without ARB 1 \| \| 1,465 \| \| 348 \| \| -- \| 64.75% \| \| \| \| \| \|  \| \| \| \| \| \| \| \| \| \| \| \| \| \| \| \| \|  \|  \| \|  \| \| \| χ^2^ \| \| df \| \| p \|  \|  \|  \| \| \| \|  \|  \| \| **Log-Rank Test** \| \| \| 0.644 \| \| 1 \| \| 0.422 \|  \|  \|  \| \| \| \|  \| \| \| \| \| \| \| \| \| \| \| \| \| \| \| \| \|  \|  \| \|  \| \| \| Hazard Ratio \| \| 95% CI \| \| χ^2^ \| df \| p \| \| \| \| \|  \|  \| \| **Hazard Ratio and Proportionality** \| \| \| 0.940 \| \| (0.808, 1.094) \| \| 0.585 \| 1 \| 0.444 \| \| \| \| \|  \| \| \| \| \| \| \| \| \| \| \| \| \| \| \| \| \| **Number of instances excluding patients with outcome prior to the time window** \| \| \| \| \| \| \| \| \| \| \| \| \| \| \|  \|  \| \| Cohort \| \| \| Patients in cohort \| \| Patients with outcome \| \| Mean \| Standard Deviation \| Median \| \| \| \| \|  \|  \| \| 1 \| DM+AKD+DPP4i with ACEi without ARB 1 \| \| 1,409 \| \| 322 \| \| 3.398 \| 6.975 \| 1 \| \| \| \| \|  \|  \| \| 2 \| DM+AKD without DPP4i with ACEi without ARB 1 \| \| 1,465 \| \| 348 \| \| 2.885 \| 5.485 \| 1 \| \| \| \| \|  \| \| \| \| \| \| \| \| \| \| \| \| \| \| \| \| \|  \|  \| \|  \| \| \| t \| \| df \| \| p \|  \|  \|  \| \| \| \|  \|  \| \| **Test Statistics** \| \| \| 1.061 \| \| 668 \| \| 0.289 \|  \|  \|  \| \| \| \|  \| \| \| \| \| \| \| \| \| \| \| \| \| \| \| \|   **Metformin concurrent usage (NLM:RXNORM:6809)**   \| **1 Mortality** \| \| \| \| \| \| \| \| \| \| \| \| \| --- \| --- \| --- \| --- \| --- \| --- \| --- \| --- \| --- \| --- \| --- \| --- \| \|  \| \| **Risk analysis excluding patients with outcome prior to the time window** \| \| \| \| \| \| \| \| \| \| \|  \|  \| \| \| Cohort \| \| Patients in cohort \| Patients with outcome \| Risk \| \| \| \| \|  \| \| \|  \| 1 \| DM+AKD+DPP4i with metformin 1 \| 3,006 \| 381 \| 0.127 \| \| \| \| \|  \| \| \|  \| 2 \| DM+AKD without DPP4i with metformin 1 \| 3,013 \| 382 \| 0.127 \| \| \| \| \|  \| \| \| \| \| \| \| \| \| \| \| \| \|  \| \| \|  \|  \| \|  \| 95% CI \| z \| p \|  \|  \| \|  \| \| \|  \| **Risk Difference** \| \| -0.000 \| (-0.017, 0.017) \| -0.004 \| 0.997 \|  \|  \| \|  \| \| \|  \| **Risk Ratio** \| \| 1.000 \| (0.876, 1.141) \| N/A \| N/A \|  \|  \| \|  \| \| \|  \| **Odds Ratio** \| \| 1.000 \| (0.859, 1.164) \| N/A \| N/A \|  \|  \| \|  \| \| \| \| \| \| \| \| \| \| \| \| \|  \| \| **Kaplan - Meier survival analysis excluding patients with outcome prior to the time window** \| \| \| \| \| \| \| \| \| \| \|  \| \| \|  \| Cohort \| \| Patients in cohort \| Patients with outcome \| Median survival (days) \| Survival probability at end of time window \| \| \| \|  \| \| \|  \| 1 \| DM+AKD+DPP4i with metformin 1 \| 3,006 \| 381 \| -- \| 79.54% \| \| \| \|  \| \| \|  \| 2 \| DM+AKD without DPP4i with metformin 1 \| 3,013 \| 382 \| -- \| 78.63% \| \| \| \|  \| \| \| \| \| \| \| \| \| \| \| \| \|  \| \| \|  \|  \| \| χ^2^ \| df \| p \|  \|  \|  \| \|  \| \| \|  \| **Log-Rank Test** \| \| 0.240 \| 1 \| 0.624 \|  \|  \|  \| \|  \| \| \| \| \| \| \| \| \| \| \| \| \|  \| \| \|  \|  \| \| Hazard Ratio \| 95% CI \| χ^2^ \| df \| p \| \| \|  \| \| \|  \| **Hazard Ratio and Proportionality** \| \| 0.965 \| (0.837, 1.112) \| 0.230 \| 1 \| 0.631 \| \| \|  \| \| \| \| \| \| \| \| \| \| \| \| \|  \| \| **Number of instances excluding patients with outcome prior to the time window** \| \| \| \| \| \| \| \| \| \| \|  \| \| \|  \| Cohort \| \| Patients in cohort \| Patients with outcome \| Mean \| Standard Deviation \| Median \| \| \|  \| \| \|  \| 1 \| DM+AKD+DPP4i with metformin 1 \| 3,006 \| 381 \| 1.207 \| 0.595 \| 1 \| \| \|  \| \| \|  \| 2 \| DM+AKD without DPP4i with metformin 1 \| 3,013 \| 382 \| 1.204 \| 0.602 \| 1 \| \| \|  \| \| \| \| \| \| \| \| \| \| \| \| \|  \| \| \|  \|  \| \| t \| df \| p \|  \|  \|  \| \|  \| \| \|  \| **Test Statistics** \| \| 0.073 \| 761 \| 0.942 \|  \|  \|  \| \|  \| \| \| \| \| \| \| \| \| \| \| \| \| **2 MAKE** \| \| \| \| \| \| \| \| \| \| \| \| \|  \| \| **Risk analysis** \| \| \| \| \| \| \| \| \| \| \|  \|  \| \| \| Cohort \| \| Patients in cohort \| Patients with outcome \| Risk \| \| \| \| \|  \| \| \|  \| 1 \| DM+AKD+DPP4i with metformin 1 \| 3,167 \| 418 \| 0.132 \| \| \| \| \|  \| \| \|  \| 2 \| DM+AKD without DPP4i with metformin 1 \| 3,167 \| 412 \| 0.130 \| \| \| \| \|  \| \| \| \| \| \| \| \| \| \| \| \| \|  \| \| \|  \|  \| \|  \| 95% CI \| z \| p \|  \|  \| \|  \| \| \|  \| **Risk Difference** \| \| 0.002 \| (-0.015, 0.019) \| 0.223 \| 0.823 \|  \|  \| \|  \| \| \|  \| **Risk Ratio** \| \| 1.015 \| (0.894, 1.152) \| N/A \| N/A \|  \|  \| \|  \| \| \|  \| **Odds Ratio** \| \| 1.017 \| (0.879, 1.177) \| N/A \| N/A \|  \|  \| \|  \| \| \| \| \| \| \| \| \| \| \| \| \|  \| \| **Kaplan - Meier survival analysis** \| \| \| \| \| \| \| \| \| \| \|  \| \| \|  \| Cohort \| \| Patients in cohort \| Patients with outcome \| Median survival (days) \| Survival probability at end of time window \| \| \| \|  \| \| \|  \| 1 \| DM+AKD+DPP4i with metformin 1 \| 3,167 \| 418 \| -- \| 78.63% \| \| \| \|  \| \| \|  \| 2 \| DM+AKD without DPP4i with metformin 1 \| 3,167 \| 412 \| -- \| 78.09% \| \| \| \|  \| \| \| \| \| \| \| \| \| \| \| \| \|  \| \| \|  \|  \| \| χ^2^ \| df \| p \|  \|  \|  \| \|  \| \| \|  \| **Log-Rank Test** \| \| 0.067 \| 1 \| 0.796 \|  \|  \|  \| \|  \| \| \| \| \| \| \| \| \| \| \| \| \|  \| \| \|  \|  \| \| Hazard Ratio \| 95% CI \| χ^2^ \| df \| p \| \| \|  \| \| \|  \| **Hazard Ratio and Proportionality** \| \| 0.982 \| (0.857, 1.125) \| 0.161 \| 1 \| 0.688 \| \| \|  \| \| \| \| \| \| \| \| \| \| \| \| \| **3 MACE** \| \| \| \| \| \| \| \| \| \| \| \| \|  \| \| **Risk analysis excluding patients with outcome prior to the time window** \| \| \| \| \| \| \| \| \| \| \|  \|  \| \| \| Cohort \| \| Patients in cohort \| Patients with outcome \| Risk \| \| \| \| \|  \| \| \|  \| 1 \| DM+AKD+DPP4i with metformin 1 \| 2,158 \| 409 \| 0.190 \| \| \| \| \|  \| \| \|  \| 2 \| DM+AKD without DPP4i with metformin 1 \| 2,207 \| 408 \| 0.185 \| \| \| \| \|  \| \| \| \| \| \| \| \| \| \| \| \| \|  \| \| \|  \|  \| \|  \| 95% CI \| z \| p \|  \|  \| \|  \| \| \|  \| **Risk Difference** \| \| 0.005 \| (-0.018, 0.028) \| 0.395 \| 0.693 \|  \|  \| \|  \| \| \|  \| **Risk Ratio** \| \| 1.025 \| (0.906, 1.160) \| N/A \| N/A \|  \|  \| \|  \| \| \|  \| **Odds Ratio** \| \| 1.031 \| (0.886, 1.201) \| N/A \| N/A \|  \|  \| \|  \| \| \| \| \| \| \| \| \| \| \| \| \|  \| \| **Kaplan - Meier survival analysis excluding patients with outcome prior to the time window** \| \| \| \| \| \| \| \| \| \| \|  \| \| \|  \| Cohort \| \| Patients in cohort \| Patients with outcome \| Median survival (days) \| Survival probability at end of time window \| \| \| \|  \| \| \|  \| 1 \| DM+AKD+DPP4i with metformin 1 \| 2,158 \| 409 \| -- \| 70.43% \| \| \| \|  \| \| \|  \| 2 \| DM+AKD without DPP4i with metformin 1 \| 2,207 \| 408 \| -- \| 70.32% \| \| \| \|  \| \| \| \| \| \| \| \| \| \| \| \| \|  \| \| \|  \|  \| \| χ^2^ \| df \| p \|  \|  \|  \| \|  \| \| \|  \| **Log-Rank Test** \| \| 0.032 \| 1 \| 0.858 \|  \|  \|  \| \|  \| \| \| \| \| \| \| \| \| \| \| \| \|  \| \| \|  \|  \| \| Hazard Ratio \| 95% CI \| χ^2^ \| df \| p \| \| \|  \| \| \|  \| **Hazard Ratio and Proportionality** \| \| 0.988 \| (0.861, 1.133) \| 0.090 \| 1 \| 0.764 \| \| \|  \| \| \| \| \| \| \| \| \| \| \| \| \|  \| \| **Number of instances excluding patients with outcome prior to the time window** \| \| \| \| \| \| \| \| \| \| \|  \| \| \|  \| Cohort \| \| Patients in cohort \| Patients with outcome \| Mean \| Standard Deviation \| Median \| \| \|  \| \| \|  \| 1 \| DM+AKD+DPP4i with metformin 1 \| 2,158 \| 409 \| 4.235 \| 12.090 \| 1 \| \| \|  \| \| \|  \| 2 \| DM+AKD without DPP4i with metformin 1 \| 2,207 \| 408 \| 3.529 \| 9.770 \| 1 \| \| \|  \| \| \| \| \| \| \| \| \| \| \| \| \|  \| \| \|  \|  \| \| t \| df \| p \|  \|  \|  \| \|  \| \| \|  \| **Test Statistics** \| \| 0.917 \| 815 \| 0.359 \|  \|  \|  \| \|  \| \| \| \| \| \| \| \| \| \| \| \|   **Without metformin concurrent usage**   \| **1 Mortality** \| \| \| \| \| \| \| \| \| \| \| \| \| --- \| --- \| --- \| --- \| --- \| --- \| --- \| --- \| --- \| --- \| --- \| --- \| \|  \| \| **Risk analysis excluding patients with outcome prior to the time window** \| \| \| \| \| \| \| \| \| \| \|  \|  \| \| \| Cohort \| \| Patients in cohort \| Patients with outcome \| Risk \| \| \| \| \|  \| \| \|  \| 1 \| DM+AKD+DPP4i without metformin 1 \| 3,406 \| 584 \| 0.171 \| \| \| \| \|  \| \| \|  \| 2 \| DM+AKD without DPP4i without metformin 1 \| 3,353 \| 609 \| 0.182 \| \| \| \| \|  \| \| \| \| \| \| \| \| \| \| \| \| \|  \| \| \|  \|  \| \|  \| 95% CI \| z \| p \|  \|  \| \|  \| \| \|  \| **Risk Difference** \| \| -0.010 \| (-0.028, 0.008) \| -1.096 \| 0.273 \|  \|  \| \|  \| \| \|  \| **Risk Ratio** \| \| 0.944 \| (0.852, 1.046) \| N/A \| N/A \|  \|  \| \|  \| \| \|  \| **Odds Ratio** \| \| 0.932 \| (0.823, 1.057) \| N/A \| N/A \|  \|  \| \|  \| \| \| \| \| \| \| \| \| \| \| \| \|  \| \| **Kaplan - Meier survival analysis excluding patients with outcome prior to the time window** \| \| \| \| \| \| \| \| \| \| \|  \| \| \|  \| Cohort \| \| Patients in cohort \| Patients with outcome \| Median survival (days) \| Survival probability at end of time window \| \| \| \|  \| \| \|  \| 1 \| DM+AKD+DPP4i without metformin 1 \| 3,406 \| 584 \| -- \| 69.61% \| \| \| \|  \| \| \|  \| 2 \| DM+AKD without DPP4i without metformin 1 \| 3,353 \| 609 \| -- \| 66.18% \| \| \| \|  \| \| \| \| \| \| \| \| \| \| \| \| \|  \| \| \|  \|  \| \| χ^2^ \| df \| p \|  \|  \|  \| \|  \| \| \|  \| **Log-Rank Test** \| \| 6.007 \| 1 \| 0.014 \|  \|  \|  \| \|  \| \| \| \| \| \| \| \| \| \| \| \| \|  \| \| \|  \|  \| \| Hazard Ratio \| 95% CI \| χ^2^ \| df \| p \| \| \|  \| \| \|  \| **Hazard Ratio and Proportionality** \| \| 0.868 \| (0.775, 0.972) \| 0.559 \| 1 \| 0.455 \| \| \|  \| \| \| \| \| \| \| \| \| \| \| \| \|  \| \| **Number of instances excluding patients with outcome prior to the time window** \| \| \| \| \| \| \| \| \| \| \|  \| \| \|  \| Cohort \| \| Patients in cohort \| Patients with outcome \| Mean \| Standard Deviation \| Median \| \| \|  \| \| \|  \| 1 \| DM+AKD+DPP4i without metformin 1 \| 3,406 \| 584 \| 1.216 \| 0.448 \| 1 \| \| \|  \| \| \|  \| 2 \| DM+AKD without DPP4i without metformin 1 \| 3,353 \| 609 \| 1.159 \| 0.392 \| 1 \| \| \|  \| \| \| \| \| \| \| \| \| \| \| \| \|  \| \| \|  \|  \| \| t \| df \| p \|  \|  \|  \| \|  \| \| \|  \| **Test Statistics** \| \| 2.320 \| 1191 \| 0.020 \|  \|  \|  \| \|  \| \| \| \| \| \| \| \| \| \| \| \| \| **2 MAKE** \| \| \| \| \| \| \| \| \| \| \| \| \|  \| \| **Risk analysis** \| \| \| \| \| \| \| \| \| \| \|  \|  \| \| \| Cohort \| \| Patients in cohort \| Patients with outcome \| Risk \| \| \| \| \|  \| \| \|  \| 1 \| DM+AKD+DPP4i without metformin 1 \| 3,583 \| 662 \| 0.185 \| \| \| \| \|  \| \| \|  \| 2 \| DM+AKD without DPP4i without metformin 1 \| 3,583 \| 661 \| 0.184 \| \| \| \| \|  \| \| \| \| \| \| \| \| \| \| \| \| \|  \| \| \|  \|  \| \|  \| 95% CI \| z \| p \|  \|  \| \|  \| \| \|  \| **Risk Difference** \| \| 0.000 \| (-0.018, 0.018) \| 0.030 \| 0.976 \|  \|  \| \|  \| \| \|  \| **Risk Ratio** \| \| 1.002 \| (0.909, 1.104) \| N/A \| N/A \|  \|  \| \|  \| \| \|  \| **Odds Ratio** \| \| 1.002 \| (0.889, 1.129) \| N/A \| N/A \|  \|  \| \|  \| \| \| \| \| \| \| \| \| \| \| \| \|  \| \| **Kaplan - Meier survival analysis** \| \| \| \| \| \| \| \| \| \| \|  \| \| \|  \| Cohort \| \| Patients in cohort \| Patients with outcome \| Median survival (days) \| Survival probability at end of time window \| \| \| \|  \| \| \|  \| 1 \| DM+AKD+DPP4i without metformin 1 \| 3,583 \| 662 \| -- \| 67.51% \| \| \| \|  \| \| \|  \| 2 \| DM+AKD without DPP4i without metformin 1 \| 3,583 \| 661 \| -- \| 65.05% \| \| \| \|  \| \| \| \| \| \| \| \| \| \| \| \| \|  \| \| \|  \|  \| \| χ^2^ \| df \| p \|  \|  \|  \| \|  \| \| \|  \| **Log-Rank Test** \| \| 4.187 \| 1 \| 0.041 \|  \|  \|  \| \|  \| \| \| \| \| \| \| \| \| \| \| \| \|  \| \| \|  \|  \| \| Hazard Ratio \| 95% CI \| χ^2^ \| df \| p \| \| \|  \| \| \|  \| **Hazard Ratio and Proportionality** \| \| 0.894 \| (0.802, 0.995) \| 1.500 \| 1 \| 0.221 \| \| \|  \| \| \| \| \| \| \| \| \| \| \| \| \| **3 MACE** \| \| \| \| \| \| \| \| \| \| \| \| \|  \| \| **Risk analysis excluding patients with outcome prior to the time window** \| \| \| \| \| \| \| \| \| \| \|  \|  \| \| \| Cohort \| \| Patients in cohort \| Patients with outcome \| Risk \| \| \| \| \|  \| \| \|  \| 1 \| DM+AKD+DPP4i without metformin 1 \| 2,413 \| 527 \| 0.218 \| \| \| \| \|  \| \| \|  \| 2 \| DM+AKD without DPP4i without metformin 1 \| 2,466 \| 598 \| 0.242 \| \| \| \| \|  \| \| \| \| \| \| \| \| \| \| \| \| \|  \| \| \|  \|  \| \|  \| 95% CI \| z \| p \|  \|  \| \|  \| \| \|  \| **Risk Difference** \| \| -0.024 \| (-0.048, -0.000) \| -1.998 \| 0.046 \|  \|  \| \|  \| \| \|  \| **Risk Ratio** \| \| 0.901 \| (0.813, 0.998) \| N/A \| N/A \|  \|  \| \|  \| \| \|  \| **Odds Ratio** \| \| 0.873 \| (0.764, 0.997) \| N/A \| N/A \|  \|  \| \|  \| \| \| \| \| \| \| \| \| \| \| \| \|  \| \| **Kaplan - Meier survival analysis excluding patients with outcome prior to the time window** \| \| \| \| \| \| \| \| \| \| \|  \| \| \|  \| Cohort \| \| Patients in cohort \| Patients with outcome \| Median survival (days) \| Survival probability at end of time window \| \| \| \|  \| \| \|  \| 1 \| DM+AKD+DPP4i without metformin 1 \| 2,413 \| 527 \| -- \| 62.53% \| \| \| \|  \| \| \|  \| 2 \| DM+AKD without DPP4i without metformin 1 \| 2,466 \| 598 \| -- \| 56.50% \| \| \| \|  \| \| \| \| \| \| \| \| \| \| \| \| \|  \| \| \|  \|  \| \| χ^2^ \| df \| p \|  \|  \|  \| \|  \| \| \|  \| **Log-Rank Test** \| \| 13.466 \| 1 \| 0.000 \|  \|  \|  \| \|  \| \| \| \| \| \| \| \| \| \| \| \| \|  \| \| \|  \|  \| \| Hazard Ratio \| 95% CI \| χ^2^ \| df \| p \| \| \|  \| \| \|  \| **Hazard Ratio and Proportionality** \| \| 0.803 \| (0.715, 0.903) \| 0.759 \| 1 \| 0.384 \| \| \|  \| \| \| \| \| \| \| \| \| \| \| \| \|  \| \| **Number of instances excluding patients with outcome prior to the time window** \| \| \| \| \| \| \| \| \| \| \|  \| \| \|  \| Cohort \| \| Patients in cohort \| Patients with outcome \| Mean \| Standard Deviation \| Median \| \| \|  \| \| \|  \| 1 \| DM+AKD+DPP4i without metformin 1 \| 2,413 \| 527 \| 2.410 \| 5.563 \| 1 \| \| \|  \| \| \|  \| 2 \| DM+AKD without DPP4i without metformin 1 \| 2,466 \| 598 \| 2.278 \| 4.092 \| 1 \| \| \|  \| \| \| \| \| \| \| \| \| \| \| \| \|  \| \| \|  \|  \| \| t \| df \| p \|  \|  \|  \| \|  \| \| \|  \| **Test Statistics** \| \| 0.458 \| 1123 \| 0.647 \|  \|  \|  \| \|  \| \| \| \| \| \| \| \| \| \| \| \|   **Insulin concurrent usage (NLM:VA:HS501)**   \| **1 Mortality** \| \| \| \| \| \| \| \| \| \| \| \| \| --- \| --- \| --- \| --- \| --- \| --- \| --- \| --- \| --- \| --- \| --- \| --- \| \|  \| \| **Risk analysis excluding patients with outcome prior to the time window** \| \| \| \| \| \| \| \| \| \| \|  \|  \| \| \| Cohort \| \| Patients in cohort \| Patients with outcome \| Risk \| \| \| \| \|  \| \| \|  \| 1 \| DM+AKD+DPP4i with insulin 1 \| 3,587 \| 580 \| 0.162 \| \| \| \| \|  \| \| \|  \| 2 \| DM+AKD without DPP4i with insulin 1 \| 3,585 \| 724 \| 0.202 \| \| \| \| \|  \| \| \| \| \| \| \| \| \| \| \| \| \|  \| \| \|  \|  \| \|  \| 95% CI \| z \| p \|  \|  \| \|  \| \| \|  \| **Risk Difference** \| \| -0.040 \| (-0.058, -0.022) \| -4.420 \| 0.000 \|  \|  \| \|  \| \| \|  \| **Risk Ratio** \| \| 0.801 \| (0.725, 0.884) \| N/A \| N/A \|  \|  \| \|  \| \| \|  \| **Odds Ratio** \| \| 0.762 \| (0.676, 0.860) \| N/A \| N/A \|  \|  \| \|  \| \| \| \| \| \| \| \| \| \| \| \| \|  \| \| **Kaplan - Meier survival analysis excluding patients with outcome prior to the time window** \| \| \| \| \| \| \| \| \| \| \|  \| \| \|  \| Cohort \| \| Patients in cohort \| Patients with outcome \| Median survival (days) \| Survival probability at end of time window \| \| \| \|  \| \| \|  \| 1 \| DM+AKD+DPP4i with insulin 1 \| 3,587 \| 580 \| -- \| 73.58% \| \| \| \|  \| \| \|  \| 2 \| DM+AKD without DPP4i with insulin 1 \| 3,585 \| 724 \| -- \| 66.92% \| \| \| \|  \| \| \| \| \| \| \| \| \| \| \| \| \|  \| \| \|  \|  \| \| χ^2^ \| df \| p \|  \|  \|  \| \|  \| \| \|  \| **Log-Rank Test** \| \| 31.919 \| 1 \| 0.000 \|  \|  \|  \| \|  \| \| \| \| \| \| \| \| \| \| \| \| \|  \| \| \|  \|  \| \| Hazard Ratio \| 95% CI \| χ^2^ \| df \| p \| \| \|  \| \| \|  \| **Hazard Ratio and Proportionality** \| \| 0.731 \| (0.655, 0.815) \| 4.341 \| 1 \| 0.037 \| \| \|  \| \| \| \| \| \| \| \| \| \| \| \| \|  \| \| **Number of instances excluding patients with outcome prior to the time window** \| \| \| \| \| \| \| \| \| \| \|  \| \| \|  \| Cohort \| \| Patients in cohort \| Patients with outcome \| Mean \| Standard Deviation \| Median \| \| \|  \| \| \|  \| 1 \| DM+AKD+DPP4i with insulin 1 \| 3,587 \| 580 \| 1.219 \| 0.426 \| 1 \| \| \|  \| \| \|  \| 2 \| DM+AKD without DPP4i with insulin 1 \| 3,585 \| 724 \| 1.206 \| 0.431 \| 1 \| \| \|  \| \| \| \| \| \| \| \| \| \| \| \| \|  \| \| \|  \|  \| \| t \| df \| p \|  \|  \|  \| \|  \| \| \|  \| **Test Statistics** \| \| 0.551 \| 1302 \| 0.582 \|  \|  \|  \| \|  \| \| \| \| \| \| \| \| \| \| \| \| \| **2 MAKE** \| \| \| \| \| \| \| \| \| \| \| \| \|  \| \| **Risk analysis** \| \| \| \| \| \| \| \| \| \| \|  \|  \| \| \| Cohort \| \| Patients in cohort \| Patients with outcome \| Risk \| \| \| \| \|  \| \| \|  \| 1 \| DM+AKD+DPP4i with insulin 1 \| 3,779 \| 658 \| 0.174 \| \| \| \| \|  \| \| \|  \| 2 \| DM+AKD without DPP4i with insulin 1 \| 3,779 \| 810 \| 0.214 \| \| \| \| \|  \| \| \| \| \| \| \| \| \| \| \| \| \|  \| \| \|  \|  \| \|  \| 95% CI \| z \| p \|  \|  \| \|  \| \| \|  \| **Risk Difference** \| \| -0.040 \| (-0.058, -0.022) \| -4.420 \| 0.000 \|  \|  \| \|  \| \| \|  \| **Risk Ratio** \| \| 0.812 \| (0.741, 0.891) \| N/A \| N/A \|  \|  \| \|  \| \| \|  \| **Odds Ratio** \| \| 0.773 \| (0.689, 0.867) \| N/A \| N/A \|  \|  \| \|  \| \| \| \| \| \| \| \| \| \| \| \| \|  \| \| **Kaplan - Meier survival analysis** \| \| \| \| \| \| \| \| \| \| \|  \| \| \|  \| Cohort \| \| Patients in cohort \| Patients with outcome \| Median survival (days) \| Survival probability at end of time window \| \| \| \|  \| \| \|  \| 1 \| DM+AKD+DPP4i with insulin 1 \| 3,779 \| 658 \| -- \| 71.81% \| \| \| \|  \| \| \|  \| 2 \| DM+AKD without DPP4i with insulin 1 \| 3,779 \| 810 \| -- \| 65.12% \| \| \| \|  \| \| \| \| \| \| \| \| \| \| \| \| \|  \| \| \|  \|  \| \| χ^2^ \| df \| p \|  \|  \|  \| \|  \| \| \|  \| **Log-Rank Test** \| \| 32.489 \| 1 \| 0.000 \|  \|  \|  \| \|  \| \| \| \| \| \| \| \| \| \| \| \| \|  \| \| \|  \|  \| \| Hazard Ratio \| 95% CI \| χ^2^ \| df \| p \| \| \|  \| \| \|  \| **Hazard Ratio and Proportionality** \| \| 0.742 \| (0.670, 0.823) \| 4.155 \| 1 \| 0.042 \| \| \|  \| \| \| \| \| \| \| \| \| \| \| \| \| **3 MACE** \| \| \| \| \| \| \| \| \| \| \| \| \|  \| \| **Risk analysis excluding patients with outcome prior to the time window** \| \| \| \| \| \| \| \| \| \| \|  \|  \| \| \| Cohort \| \| Patients in cohort \| Patients with outcome \| Risk \| \| \| \| \|  \| \| \|  \| 1 \| DM+AKD+DPP4i with insulin 1 \| 2,438 \| 558 \| 0.229 \| \| \| \| \|  \| \| \|  \| 2 \| DM+AKD without DPP4i with insulin 1 \| 2,533 \| 638 \| 0.252 \| \| \| \| \|  \| \| \| \| \| \| \| \| \| \| \| \| \|  \| \| \|  \|  \| \|  \| 95% CI \| z \| p \|  \|  \| \|  \| \| \|  \| **Risk Difference** \| \| -0.023 \| (-0.047, 0.001) \| -1.896 \| 0.058 \|  \|  \| \|  \| \| \|  \| **Risk Ratio** \| \| 0.909 \| (0.823, 1.003) \| N/A \| N/A \|  \|  \| \|  \| \| \|  \| **Odds Ratio** \| \| 0.882 \| (0.774, 1.004) \| N/A \| N/A \|  \|  \| \|  \| \| \| \| \| \| \| \| \| \| \| \| \|  \| \| **Kaplan - Meier survival analysis excluding patients with outcome prior to the time window** \| \| \| \| \| \| \| \| \| \| \|  \| \| \|  \| Cohort \| \| Patients in cohort \| Patients with outcome \| Median survival (days) \| Survival probability at end of time window \| \| \| \|  \| \| \|  \| 1 \| DM+AKD+DPP4i with insulin 1 \| 2,438 \| 558 \| -- \| 64.09% \| \| \| \|  \| \| \|  \| 2 \| DM+AKD without DPP4i with insulin 1 \| 2,533 \| 638 \| -- \| 60.57% \| \| \| \|  \| \| \| \| \| \| \| \| \| \| \| \| \|  \| \| \|  \|  \| \| χ^2^ \| df \| p \|  \|  \|  \| \|  \| \| \|  \| **Log-Rank Test** \| \| 12.090 \| 1 \| 0.001 \|  \|  \|  \| \|  \| \| \| \| \| \| \| \| \| \| \| \| \|  \| \| \|  \|  \| \| Hazard Ratio \| 95% CI \| χ^2^ \| df \| p \| \| \|  \| \| \|  \| **Hazard Ratio and Proportionality** \| \| 0.818 \| (0.730, 0.916) \| 11.714 \| 1 \| 0.001 \| \| \|  \| \| \| \| \| \| \| \| \| \| \| \| \|  \| \| **Number of instances excluding patients with outcome prior to the time window** \| \| \| \| \| \| \| \| \| \| \|  \| \| \|  \| Cohort \| \| Patients in cohort \| Patients with outcome \| Mean \| Standard Deviation \| Median \| \| \|  \| \| \|  \| 1 \| DM+AKD+DPP4i with insulin 1 \| 2,438 \| 558 \| 3.195 \| 10.222 \| 1 \| \| \|  \| \| \|  \| 2 \| DM+AKD without DPP4i with insulin 1 \| 2,533 \| 638 \| 2.361 \| 4.010 \| 1 \| \| \|  \| \| \| \| \| \| \| \| \| \| \| \| \|  \| \| \|  \|  \| \| t \| df \| p \|  \|  \|  \| \|  \| \| \|  \| **Test Statistics** \| \| 1.902 \| 1194 \| 0.057 \|  \|  \|  \|   **Without insulin concurrent usage**   \| **1 Mortality** \| \| \| \| \| \| \| \| \| \| \| \| \| --- \| --- \| --- \| --- \| --- \| --- \| --- \| --- \| --- \| --- \| --- \| --- \| \|  \| \| **Risk analysis excluding patients with outcome prior to the time window** \| \| \| \| \| \| \| \| \| \| \|  \|  \| \| \| Cohort \| \| Patients in cohort \| Patients with outcome \| Risk \| \| \| \| \|  \| \| \|  \| 1 \| DM+AKD+DPP4i without insulin 1 \| 2,380 \| 354 \| 0.149 \| \| \| \| \|  \| \| \|  \| 2 \| DM+AKD without DPP4i without insulin 1 \| 2,324 \| 368 \| 0.158 \| \| \| \| \|  \| \| \| \| \| \| \| \| \| \| \| \| \|  \| \| \|  \|  \| \|  \| 95% CI \| z \| p \|  \|  \| \|  \| \| \|  \| **Risk Difference** \| \| -0.010 \| (-0.030, 0.011) \| -0.914 \| 0.361 \|  \|  \| \|  \| \| \|  \| **Risk Ratio** \| \| 0.939 \| (0.821, 1.074) \| N/A \| N/A \|  \|  \| \|  \| \| \|  \| **Odds Ratio** \| \| 0.929 \| (0.793, 1.088) \| N/A \| N/A \|  \|  \| \|  \| \| \| \| \| \| \| \| \| \| \| \| \|  \| \| **Kaplan - Meier survival analysis excluding patients with outcome prior to the time window** \| \| \| \| \| \| \| \| \| \| \|  \| \| \|  \| Cohort \| \| Patients in cohort \| Patients with outcome \| Median survival (days) \| Survival probability at end of time window \| \| \| \|  \| \| \|  \| 1 \| DM+AKD+DPP4i without insulin 1 \| 2,380 \| 354 \| -- \| 73.43% \| \| \| \|  \| \| \|  \| 2 \| DM+AKD without DPP4i without insulin 1 \| 2,324 \| 368 \| -- \| 69.58% \| \| \| \|  \| \| \| \| \| \| \| \| \| \| \| \| \|  \| \| \|  \|  \| \| χ^2^ \| df \| p \|  \|  \|  \| \|  \| \| \|  \| **Log-Rank Test** \| \| 9.122 \| 1 \| 0.003 \|  \|  \|  \| \|  \| \| \| \| \| \| \| \| \| \| \| \| \|  \| \| \|  \|  \| \| Hazard Ratio \| 95% CI \| χ^2^ \| df \| p \| \| \|  \| \| \|  \| **Hazard Ratio and Proportionality** \| \| 0.799 \| (0.691, 0.925) \| 3.638 \| 1 \| 0.056 \| \| \|  \| \| \| \| \| \| \| \| \| \| \| \| \|  \| \| **Number of instances excluding patients with outcome prior to the time window** \| \| \| \| \| \| \| \| \| \| \|  \| \| \|  \| Cohort \| \| Patients in cohort \| Patients with outcome \| Mean \| Standard Deviation \| Median \| \| \|  \| \| \|  \| 1 \| DM+AKD+DPP4i without insulin 1 \| 2,380 \| 354 \| 1.232 \| 0.645 \| 1 \| \| \|  \| \| \|  \| 2 \| DM+AKD without DPP4i without insulin 1 \| 2,324 \| 368 \| 1.196 \| 0.404 \| 1 \| \| \|  \| \| \| \| \| \| \| \| \| \| \| \| \|  \| \| \|  \|  \| \| t \| df \| p \|  \|  \|  \| \|  \| \| \|  \| **Test Statistics** \| \| 0.902 \| 720 \| 0.368 \|  \|  \|  \| \|  \| \| \| \| \| \| \| \| \| \| \| \| \| **2 MAKE** \| \| \| \| \| \| \| \| \| \| \| \| \|  \| \| **Risk analysis** \| \| \| \| \| \| \| \| \| \| \|  \|  \| \| \| Cohort \| \| Patients in cohort \| Patients with outcome \| Risk \| \| \| \| \|  \| \| \|  \| 1 \| DM+AKD+DPP4i without insulin 1 \| 2,519 \| 389 \| 0.154 \| \| \| \| \|  \| \| \|  \| 2 \| DM+AKD without DPP4i without insulin 1 \| 2,519 \| 419 \| 0.166 \| \| \| \| \|  \| \| \| \| \| \| \| \| \| \| \| \| \|  \| \| \|  \|  \| \|  \| 95% CI \| z \| p \|  \|  \| \|  \| \| \|  \| **Risk Difference** \| \| -0.012 \| (-0.032, 0.008) \| -1.152 \| 0.249 \|  \|  \| \|  \| \| \|  \| **Risk Ratio** \| \| 0.928 \| (0.818, 1.054) \| N/A \| N/A \|  \|  \| \|  \| \| \|  \| **Odds Ratio** \| \| 0.915 \| (0.787, 1.064) \| N/A \| N/A \|  \|  \| \|  \| \| \| \| \| \| \| \| \| \| \| \| \|  \| \| **Kaplan - Meier survival analysis** \| \| \| \| \| \| \| \| \| \| \|  \| \| \|  \| Cohort \| \| Patients in cohort \| Patients with outcome \| Median survival (days) \| Survival probability at end of time window \| \| \| \|  \| \| \|  \| 1 \| DM+AKD+DPP4i without insulin 1 \| 2,519 \| 389 \| -- \| 72.25% \| \| \| \|  \| \| \|  \| 2 \| DM+AKD without DPP4i without insulin 1 \| 2,519 \| 419 \| -- \| 67.56% \| \| \| \|  \| \| \| \| \| \| \| \| \| \| \| \| \|  \| \| \|  \|  \| \| χ^2^ \| df \| p \|  \|  \|  \| \|  \| \| \|  \| **Log-Rank Test** \| \| 14.150 \| 1 \| 0.000 \|  \|  \|  \| \|  \| \| \| \| \| \| \| \| \| \| \| \| \|  \| \| \|  \|  \| \| Hazard Ratio \| 95% CI \| χ^2^ \| df \| p \| \| \|  \| \| \|  \| **Hazard Ratio and Proportionality** \| \| 0.768 \| (0.669, 0.882) \| 5.036 \| 1 \| 0.025 \| \| \|  \| \| \| \| \| \| \| \| \| \| \| \| \| **3 MACE** \| \| \| \| \| \| \| \| \| \| \| \| \|  \| \| **Risk analysis excluding patients with outcome prior to the time window** \| \| \| \| \| \| \| \| \| \| \|  \|  \| \| \| Cohort \| \| Patients in cohort \| Patients with outcome \| Risk \| \| \| \| \|  \| \| \|  \| 1 \| DM+AKD+DPP4i without insulin 1 \| 1,811 \| 315 \| 0.174 \| \| \| \| \|  \| \| \|  \| 2 \| DM+AKD without DPP4i without insulin 1 \| 1,741 \| 350 \| 0.201 \| \| \| \| \|  \| \| \| \| \| \| \| \| \| \| \| \| \|  \| \| \|  \|  \| \|  \| 95% CI \| z \| p \|  \|  \| \|  \| \| \|  \| **Risk Difference** \| \| -0.027 \| (-0.053, -0.001) \| -2.070 \| 0.038 \|  \|  \| \|  \| \| \|  \| **Risk Ratio** \| \| 0.865 \| (0.754, 0.993) \| N/A \| N/A \|  \|  \| \|  \| \| \|  \| **Odds Ratio** \| \| 0.837 \| (0.707, 0.991) \| N/A \| N/A \|  \|  \| \|  \| \| \| \| \| \| \| \| \| \| \| \| \|  \| \| **Kaplan - Meier survival analysis excluding patients with outcome prior to the time window** \| \| \| \| \| \| \| \| \| \| \|  \| \| \|  \| Cohort \| \| Patients in cohort \| Patients with outcome \| Median survival (days) \| Survival probability at end of time window \| \| \| \|  \| \| \|  \| 1 \| DM+AKD+DPP4i without insulin 1 \| 1,811 \| 315 \| -- \| 70.06% \| \| \| \|  \| \| \|  \| 2 \| DM+AKD without DPP4i without insulin 1 \| 1,741 \| 350 \| -- \| 61.86% \| \| \| \|  \| \| \| \| \| \| \| \| \| \| \| \| \|  \| \| \|  \|  \| \| χ^2^ \| df \| p \|  \|  \|  \| \|  \| \| \|  \| **Log-Rank Test** \| \| 15.633 \| 1 \| 0.000 \|  \|  \|  \| \|  \| \| \| \| \| \| \| \| \| \| \| \| \|  \| \| \|  \|  \| \| Hazard Ratio \| 95% CI \| χ^2^ \| df \| p \| \| \|  \| \| \|  \| **Hazard Ratio and Proportionality** \| \| 0.736 \| (0.632, 0.858) \| 0.287 \| 1 \| 0.592 \| \| \|  \| \| \| \| \| \| \| \| \| \| \| \| \|  \| \| **Number of instances excluding patients with outcome prior to the time window** \| \| \| \| \| \| \| \| \| \| \|  \| \| \|  \| Cohort \| \| Patients in cohort \| Patients with outcome \| Mean \| Standard Deviation \| Median \| \| \|  \| \| \|  \| 1 \| DM+AKD+DPP4i without insulin 1 \| 1,811 \| 315 \| 2.457 \| 6.327 \| 1 \| \| \|  \| \| \|  \| 2 \| DM+AKD without DPP4i without insulin 1 \| 1,741 \| 350 \| 1.746 \| 2.735 \| 1 \| \| \|  \| \| \| \| \| \| \| \| \| \| \| \| \|  \| \| \|  \|  \| \| t \| df \| p \|  \|  \|  \| \|  \| \| \|  \| **Test Statistics** \| \| 1.914 \| 663 \| 0.056 \|  \|  \|  \| \|  \| \| \| \| \| \| \| \| \| \| \| \|   **GLP-1 concurrent usage (NLM:ATC:A10BJ)**   \| **1 Mortality** \| \| \| \| \| \| \| \| \| \| \| \| \| \| \| \| \| --- \| --- \| --- \| --- \| --- \| --- \| --- \| --- \| --- \| --- \| --- \| --- \| --- \| --- \| --- \| --- \| \| **Risk analysis excluding patients with outcome prior to the time window** \| \| \| \| \| \| \| \| \| \| \| \| \| \| \|  \| \| Cohort \| \| \| Patients in cohort \| \| Patients with outcome \| \| Risk \| \| \| \| \| \| \|  \|  \| \| 1 \| DM+AKD+DPP4i GLP-1 1 \| \| 404 \| \| 41 \| \| 0.101 \| \| \| \| \| \| \|  \|  \| \| 2 \| DM+AKD without DPP4i with GLP-1 1 \| \| 412 \| \| 41 \| \| 0.100 \| \| \| \| \| \| \|  \| \| \| \| \| \| \| \| \| \| \| \| \| \| \| \| \|  \|  \| \|  \| \| \|  \| \| 95% CI \| \| z \| p \|  \|  \| \| \| \|  \|  \| \| **Risk Difference** \| \| \| 0.002 \| \| (-0.039, 0.043) \| \| 0.094 \| 0.925 \|  \|  \| \| \| \|  \|  \| \| **Risk Ratio** \| \| \| 1.020 \| \| (0.676, 1.538) \| \| N/A \| N/A \|  \|  \| \| \| \|  \|  \| \| **Odds Ratio** \| \| \| 1.022 \| \| (0.648, 1.613) \| \| N/A \| N/A \|  \|  \| \| \| \|  \| \| \| \| \| \| \| \| \| \| \| \| \| \| \| \| \| **Kaplan - Meier survival analysis excluding patients with outcome prior to the time window** \| \| \| \| \| \| \| \| \| \| \| \| \| \| \|  \|  \| \| Cohort \| \| \| Patients in cohort \| \| Patients with outcome \| \| Median survival (days) \| Survival probability at end of time window \| \| \| \| \| \|  \|  \| \| 1 \| DM+AKD+DPP4i GLP-1 1 \| \| 404 \| \| 41 \| \| -- \| 79.72% \| \| \| \| \| \|  \|  \| \| 2 \| DM+AKD without DPP4i with GLP-1 1 \| \| 412 \| \| 41 \| \| -- \| 78.45% \| \| \| \| \| \|  \| \| \| \| \| \| \| \| \| \| \| \| \| \| \| \| \|  \|  \| \|  \| \| \| χ^2^ \| \| df \| \| p \|  \|  \|  \| \| \| \|  \|  \| \| **Log-Rank Test** \| \| \| 2.156 \| \| 1 \| \| 0.142 \|  \|  \|  \| \| \| \|  \| \| \| \| \| \| \| \| \| \| \| \| \| \| \| \| \|  \|  \| \|  \| \| \| Hazard Ratio \| \| 95% CI \| \| χ^2^ \| df \| p \| \| \| \| \|  \|  \| \| **Hazard Ratio and Proportionality** \| \| \| 0.723 \| \| (0.468, 1.117) \| \| 3.233 \| 1 \| 0.072 \| \| \| \| \|  \| \| \| \| \| \| \| \| \| \| \| \| \| \| \| \| \| **Number of instances excluding patients with outcome prior to the time window** \| \| \| \| \| \| \| \| \| \| \| \| \| \| \|  \|  \| \| Cohort \| \| \| Patients in cohort \| \| Patients with outcome \| \| Mean \| Standard Deviation \| Median \| \| \| \| \|  \|  \| \| 1 \| DM+AKD+DPP4i GLP-1 1 \| \| 404 \| \| 41 \| \| 1.220 \| 0.690 \| 1 \| \| \| \| \|  \|  \| \| 2 \| DM+AKD without DPP4i with GLP-1 1 \| \| 412 \| \| 41 \| \| 1.146 \| 0.527 \| 1 \| \| \| \| \|  \| \| \| \| \| \| \| \| \| \| \| \| \| \| \| \| \|  \|  \| \|  \| \| \| t \| \| df \| \| p \|  \|  \|  \| \| \| \|  \|  \| \| **Test Statistics** \| \| \| 0.540 \| \| 80 \| \| 0.591 \|  \|  \|  \| \| \| \|  \| \| \| \| \| \| \| \| \| \| \| \| \| \| \| \| \| **2 MAKE** \| \| \| \| \| \| \| \| \| \| \| \| \| \| \| \| \| **Risk analysis** \| \| \| \| \| \| \| \| \| \| \| \| \| \| \|  \| \| Cohort \| \| \| Patients in cohort \| \| Patients with outcome \| \| Risk \| \| \| \| \| \| \|  \|  \| \| 1 \| DM+AKD+DPP4i GLP-1 1 \| \| 435 \| \| 48 \| \| 0.110 \| \| \| \| \| \| \|  \|  \| \| 2 \| DM+AKD without DPP4i with GLP-1 1 \| \| 435 \| \| 44 \| \| 0.101 \| \| \| \| \| \| \|  \| \| \| \| \| \| \| \| \| \| \| \| \| \| \| \| \|  \|  \| \|  \| \| \|  \| \| 95% CI \| \| z \| p \|  \|  \| \| \| \|  \|  \| \| **Risk Difference** \| \| \| 0.009 \| \| (-0.032, 0.050) \| \| 0.441 \| 0.659 \|  \|  \| \| \| \|  \|  \| \| **Risk Ratio** \| \| \| 1.091 \| \| (0.741, 1.606) \| \| N/A \| N/A \|  \|  \| \| \| \|  \|  \| \| **Odds Ratio** \| \| \| 1.102 \| \| (0.715, 1.699) \| \| N/A \| N/A \|  \|  \| \| \| \|  \| \| \| \| \| \| \| \| \| \| \| \| \| \| \| \| \| **Kaplan - Meier survival analysis** \| \| \| \| \| \| \| \| \| \| \| \| \| \| \|  \|  \| \| Cohort \| \| \| Patients in cohort \| \| Patients with outcome \| \| Median survival (days) \| Survival probability at end of time window \| \| \| \| \| \|  \|  \| \| 1 \| DM+AKD+DPP4i GLP-1 1 \| \| 435 \| \| 48 \| \| -- \| 77.45% \| \| \| \| \| \|  \|  \| \| 2 \| DM+AKD without DPP4i with GLP-1 1 \| \| 435 \| \| 44 \| \| -- \| 78.83% \| \| \| \| \| \|  \| \| \| \| \| \| \| \| \| \| \| \| \| \| \| \| \|  \|  \| \|  \| \| \| χ^2^ \| \| df \| \| p \|  \|  \|  \| \| \| \|  \|  \| \| **Log-Rank Test** \| \| \| 1.370 \| \| 1 \| \| 0.242 \|  \|  \|  \| \| \| \|  \| \| \| \| \| \| \| \| \| \| \| \| \| \| \| \| \|  \|  \| \|  \| \| \| Hazard Ratio \| \| 95% CI \| \| χ^2^ \| df \| p \| \| \| \| \|  \|  \| \| **Hazard Ratio and Proportionality** \| \| \| 0.783 \| \| (0.519, 1.181) \| \| 4.813 \| 1 \| 0.028 \| \| \| \| \| **3 MACE** \| \| \| \| \| \| \| \| \| \| \| \| \| \| \| \| \| **Risk analysis excluding patients with outcome prior to the time window** \| \| \| \| \| \| \| \| \| \| \| \| \| \| \|  \| \| Cohort \| \| \| Patients in cohort \| \| Patients with outcome \| \| Risk \| \| \| \| \| \| \|  \|  \| \| 1 \| DM+AKD+DPP4i GLP-1 1 \| \| 306 \| \| 54 \| \| 0.176 \| \| \| \| \| \| \|  \|  \| \| 2 \| DM+AKD without DPP4i with GLP-1 1 \| \| 293 \| \| 34 \| \| 0.116 \| \| \| \| \| \| \|  \| \| \| \| \| \| \| \| \| \| \| \| \| \| \| \| \|  \|  \| \|  \| \| \|  \| \| 95% CI \| \| z \| p \|  \|  \| \| \| \|  \|  \| \| **Risk Difference** \| \| \| 0.060 \| \| (0.004, 0.117) \| \| 2.088 \| 0.037 \|  \|  \| \| \| \|  \|  \| \| **Risk Ratio** \| \| \| 1.521 \| \| (1.021, 2.264) \| \| N/A \| N/A \|  \|  \| \| \| \|  \|  \| \| **Odds Ratio** \| \| \| 1.632 \| \| (1.028, 2.593) \| \| N/A \| N/A \|  \|  \| \| \| \|  \| \| \| \| \| \| \| \| \| \| \| \| \| \| \| \| \| **Kaplan - Meier survival analysis excluding patients with outcome prior to the time window** \| \| \| \| \| \| \| \| \| \| \| \| \| \| \|  \|  \| \| Cohort \| \| \| Patients in cohort \| \| Patients with outcome \| \| Median survival (days) \| Survival probability at end of time window \| \| \| \| \| \|  \|  \| \| 1 \| DM+AKD+DPP4i GLP-1 1 \| \| 306 \| \| 54 \| \| -- \| 69.10% \| \| \| \| \| \|  \|  \| \| 2 \| DM+AKD without DPP4i with GLP-1 1 \| \| 293 \| \| 34 \| \| -- \| 71.93% \| \| \| \| \| \|  \| \| \| \| \| \| \| \| \| \| \| \| \| \| \| \| \|  \|  \| \|  \| \| \| χ^2^ \| \| df \| \| p \|  \|  \|  \| \| \| \|  \|  \| \| **Log-Rank Test** \| \| \| 0.059 \| \| 1 \| \| 0.809 \|  \|  \|  \| \| \| \|  \| \| \| \| \| \| \| \| \| \| \| \| \| \| \| \| \|  \|  \| \|  \| \| \| Hazard Ratio \| \| 95% CI \| \| χ^2^ \| df \| p \| \| \| \| \|  \|  \| \| **Hazard Ratio and Proportionality** \| \| \| 1.055 \| \| (0.685, 1.624) \| \| 0.322 \| 1 \| 0.571 \| \| \| \| \|  \| \| \| \| \| \| \| \| \| \| \| \| \| \| \| \| \| **Number of instances excluding patients with outcome prior to the time window** \| \| \| \| \| \| \| \| \| \| \| \| \| \| \|  \|  \| \| Cohort \| \| \| Patients in cohort \| \| Patients with outcome \| \| Mean \| Standard Deviation \| Median \| \| \| \| \|  \|  \| \| 1 \| DM+AKD+DPP4i GLP-1 1 \| \| 306 \| \| 54 \| \| 2.852 \| 3.739 \| 1 \| \| \| \| \|  \|  \| \| 2 \| DM+AKD without DPP4i with GLP-1 1 \| \| 293 \| \| 34 \| \| 1.559 \| 1.307 \| 1 \| \| \| \| \|  \| \| \| \| \| \| \| \| \| \| \| \| \| \| \| \| \|  \|  \| \|  \| \| \| t \| \| df \| \| p \|  \|  \|  \| \| \| \|  \|  \| \| **Test Statistics** \| \| \| 1.940 \| \| 86 \| \| 0.056 \|  \|  \|  \| \| \| \|  \| \| \| \| \| \| \| \| \| \| \| \| \| \| \| \|   **Without GLP-1 concurrent usage**   \| **1 Mortality** \| \| \| \| \| \| \| \| \| \| \| \| \| \| \| \| \| --- \| --- \| --- \| --- \| --- \| --- \| --- \| --- \| --- \| --- \| --- \| --- \| --- \| --- \| --- \| --- \| \| **Risk analysis excluding patients with outcome prior to the time window** \| \| \| \| \| \| \| \| \| \| \| \| \| \| \|  \| \| Cohort \| \| \| Patients in cohort \| \| Patients with outcome \| \| Risk \| \| \| \| \| \| \|  \|  \| \| 1 \| DM+AKD+DPP4i without GLP-1 1 \| \| 6,158 \| \| 958 \| \| 0.156 \| \| \| \| \| \| \|  \|  \| \| 2 \| DM+AKD without DPP4i without GLP-1 1 \| \| 6,074 \| \| 1,002 \| \| 0.165 \| \| \| \| \| \| \|  \| \| \| \| \| \| \| \| \| \| \| \| \| \| \| \| \|  \|  \| \|  \| \| \|  \| \| 95% CI \| \| z \| p \|  \|  \| \| \| \|  \|  \| \| **Risk Difference** \| \| \| -0.009 \| \| (-0.022, 0.004) \| \| -1.416 \| 0.157 \|  \|  \| \| \| \|  \|  \| \| **Risk Ratio** \| \| \| 0.943 \| \| (0.870, 1.023) \| \| N/A \| N/A \|  \|  \| \| \| \|  \|  \| \| **Odds Ratio** \| \| \| 0.933 \| \| (0.847, 1.027) \| \| N/A \| N/A \|  \|  \| \| \| \|  \| \| \| \| \| \| \| \| \| \| \| \| \| \| \| \| \| **Kaplan - Meier survival analysis excluding patients with outcome prior to the time window** \| \| \| \| \| \| \| \| \| \| \| \| \| \| \|  \|  \| \| Cohort \| \| \| Patients in cohort \| \| Patients with outcome \| \| Median survival (days) \| Survival probability at end of time window \| \| \| \| \| \|  \|  \| \| 1 \| DM+AKD+DPP4i without GLP-1 1 \| \| 6,158 \| \| 958 \| \| -- \| 73.16% \| \| \| \| \| \|  \|  \| \| 2 \| DM+AKD without DPP4i without GLP-1 1 \| \| 6,074 \| \| 1,002 \| \| -- \| 70.70% \| \| \| \| \| \|  \| \| \| \| \| \| \| \| \| \| \| \| \| \| \| \| \|  \|  \| \|  \| \| \| χ^2^ \| \| df \| \| p \|  \|  \|  \| \| \| \|  \|  \| \| **Log-Rank Test** \| \| \| 11.106 \| \| 1 \| \| 0.001 \|  \|  \|  \| \| \| \|  \| \| \| \| \| \| \| \| \| \| \| \| \| \| \| \| \|  \|  \| \|  \| \| \| Hazard Ratio \| \| 95% CI \| \| χ^2^ \| df \| p \| \| \| \| \|  \|  \| \| **Hazard Ratio and Proportionality** \| \| \| 0.860 \| \| (0.787, 0.940) \| \| 6.884 \| 1 \| 0.009 \| \| \| \| \|  \| \| \| \| \| \| \| \| \| \| \| \| \| \| \| \| \| **Number of instances excluding patients with outcome prior to the time window** \| \| \| \| \| \| \| \| \| \| \| \| \| \| \|  \|  \| \| Cohort \| \| \| Patients in cohort \| \| Patients with outcome \| \| Mean \| Standard Deviation \| Median \| \| \| \| \|  \|  \| \| 1 \| DM+AKD+DPP4i without GLP-1 1 \| \| 6,158 \| \| 958 \| \| 1.211 \| 0.498 \| 1 \| \| \| \| \|  \|  \| \| 2 \| DM+AKD without DPP4i without GLP-1 1 \| \| 6,074 \| \| 1,002 \| \| 1.220 \| 0.616 \| 1 \| \| \| \| \|  \| \| \| \| \| \| \| \| \| \| \| \| \| \| \| \| \|  \|  \| \|  \| \| \| t \| \| df \| \| p \|  \|  \|  \| \| \| \|  \|  \| \| **Test Statistics** \| \| \| -0.343 \| \| 1958 \| \| 0.732 \|  \|  \|  \| \| \| \|  \| \| \| \| \| \| \| \| \| \| \| \| \| \| \| \| \| **2 MAKE** \| \| \| \| \| \| \| \| \| \| \| \| \| \| \| \| \| **Risk analysis** \| \| \| \| \| \| \| \| \| \| \| \| \| \| \|  \| \| Cohort \| \| \| Patients in cohort \| \| Patients with outcome \| \| Risk \| \| \| \| \| \| \|  \|  \| \| 1 \| DM+AKD+DPP4i without GLP-1 1 \| \| 6,483 \| \| 1,065 \| \| 0.164 \| \| \| \| \| \| \|  \|  \| \| 2 \| DM+AKD without DPP4i without GLP-1 1 \| \| 6,483 \| \| 1,130 \| \| 0.174 \| \| \| \| \| \| \|  \| \| \| \| \| \| \| \| \| \| \| \| \| \| \| \| \|  \|  \| \|  \| \| \|  \| \| 95% CI \| \| z \| p \|  \|  \| \| \| \|  \|  \| \| **Risk Difference** \| \| \| -0.010 \| \| (-0.023, 0.003) \| \| -1.522 \| 0.128 \|  \|  \| \| \| \|  \|  \| \| **Risk Ratio** \| \| \| 0.942 \| \| (0.873, 1.017) \| \| N/A \| N/A \|  \|  \| \| \| \|  \|  \| \| **Odds Ratio** \| \| \| 0.931 \| \| (0.849, 1.021) \| \| N/A \| N/A \|  \|  \| \| \| \|  \| \| \| \| \| \| \| \| \| \| \| \| \| \| \| \| \| **Kaplan - Meier survival analysis** \| \| \| \| \| \| \| \| \| \| \| \| \| \| \|  \|  \| \| Cohort \| \| \| Patients in cohort \| \| Patients with outcome \| \| Median survival (days) \| Survival probability at end of time window \| \| \| \| \| \|  \|  \| \| 1 \| DM+AKD+DPP4i without GLP-1 1 \| \| 6,483 \| \| 1,065 \| \| -- \| 71.74% \| \| \| \| \| \|  \|  \| \| 2 \| DM+AKD without DPP4i without GLP-1 1 \| \| 6,483 \| \| 1,130 \| \| -- \| 68.46% \| \| \| \| \| \|  \| \| \| \| \| \| \| \| \| \| \| \| \| \| \| \| \|  \|  \| \|  \| \| \| χ^2^ \| \| df \| \| p \|  \|  \|  \| \| \| \|  \|  \| \| **Log-Rank Test** \| \| \| 16.992 \| \| 1 \| \| 0.000 \|  \|  \|  \| \| \| \|  \| \| \| \| \| \| \| \| \| \| \| \| \| \| \| \| \|  \|  \| \|  \| \| \| Hazard Ratio \| \| 95% CI \| \| χ^2^ \| df \| p \| \| \| \| \|  \|  \| \| **Hazard Ratio and Proportionality** \| \| \| 0.839 \| \| (0.771, 0.912) \| \| 6.720 \| 1 \| 0.010 \| \| \| \| \|  \| \| \| \| \| \| \| \| \| \| \| \| \| \| \| \| \| **3 MACE** \| \| \| \| \| \| \| \| \| \| \| \| \| \| \| \| \| **Risk analysis excluding patients with outcome prior to the time window** \| \| \| \| \| \| \| \| \| \| \| \| \| \| \|  \| \| Cohort \| \| \| Patients in cohort \| \| Patients with outcome \| \| Risk \| \| \| \| \| \| \|  \|  \| \| 1 \| DM+AKD+DPP4i without GLP-1 1 \| \| 4,324 \| \| 890 \| \| 0.206 \| \| \| \| \| \| \|  \|  \| \| 2 \| DM+AKD without DPP4i without GLP-1 1 \| \| 4,455 \| \| 973 \| \| 0.218 \| \| \| \| \| \| \|  \| \| \| \| \| \| \| \| \| \| \| \| \| \| \| \| \|  \|  \| \|  \| \| \|  \| \| 95% CI \| \| z \| p \|  \|  \| \| \| \|  \|  \| \| **Risk Difference** \| \| \| -0.013 \| \| (-0.030, 0.005) \| \| -1.441 \| 0.150 \|  \|  \| \| \| \|  \|  \| \| **Risk Ratio** \| \| \| 0.942 \| \| (0.869, 1.022) \| \| N/A \| N/A \|  \|  \| \| \| \|  \|  \| \| **Odds Ratio** \| \| \| 0.927 \| \| (0.837, 1.027) \| \| N/A \| N/A \|  \|  \| \| \| \|  \| \| \| \| \| \| \| \| \| \| \| \| \| \| \| \| \| **Kaplan - Meier survival analysis excluding patients with outcome prior to the time window** \| \| \| \| \| \| \| \| \| \| \| \| \| \| \|  \|  \| \| Cohort \| \| \| Patients in cohort \| \| Patients with outcome \| \| Median survival (days) \| Survival probability at end of time window \| \| \| \| \| \|  \|  \| \| 1 \| DM+AKD+DPP4i without GLP-1 1 \| \| 4,324 \| \| 890 \| \| -- \| 65.85% \| \| \| \| \| \|  \|  \| \| 2 \| DM+AKD without DPP4i without GLP-1 1 \| \| 4,455 \| \| 973 \| \| -- \| 62.15% \| \| \| \| \| \|  \| \| \| \| \| \| \| \| \| \| \| \| \| \| \| \| \|  \|  \| \|  \| \| \| χ^2^ \| \| df \| \| p \|  \|  \|  \| \| \| \|  \|  \| \| **Log-Rank Test** \| \| \| 12.613 \| \| 1 \| \| 0.000 \|  \|  \|  \| \| \| \|  \| \| \| \| \| \| \| \| \| \| \| \| \| \| \| \| \|  \|  \| \|  \| \| \| Hazard Ratio \| \| 95% CI \| \| χ^2^ \| df \| p \| \| \| \| \|  \|  \| \| **Hazard Ratio and Proportionality** \| \| \| 0.848 \| \| (0.775, 0.929) \| \| 3.255 \| 1 \| 0.071 \| \| \| \| \|  \| \| \| \| \| \| \| \| \| \| \| \| \| \| \| \| \| **Number of instances excluding patients with outcome prior to the time window** \| \| \| \| \| \| \| \| \| \| \| \| \| \| \|  \|  \| \| Cohort \| \| \| Patients in cohort \| \| Patients with outcome \| \| Mean \| Standard Deviation \| Median \| \| \| \| \|  \|  \| \| 1 \| DM+AKD+DPP4i without GLP-1 1 \| \| 4,324 \| \| 890 \| \| 3.074 \| 8.825 \| 1 \| \| \| \| \|  \|  \| \| 2 \| DM+AKD without DPP4i without GLP-1 1 \| \| 4,455 \| \| 973 \| \| 2.329 \| 4.099 \| 1 \| \| \| \| \|  \| \| \| \| \| \| \| \| \| \| \| \| \| \| \| \| \|  \|  \| \|  \| \| \| t \| \| df \| \| p \|  \|  \|  \| \| \| \|  \|  \| \| **Test Statistics** \| \| \| 2.370 \| \| 1861 \| \| 0.018 \|  \|  \|  \| \| \| \|  \| \| \| \| \| \| \| \| \| \| \| \| \| \| \| \|   **SGLT-2 concurrent usage (NLM:ATC:A10BK)**   \| **1 Mortality** \| \| \| \| \| \| \| \| \| \| \| \| \| --- \| --- \| --- \| --- \| --- \| --- \| --- \| --- \| --- \| --- \| --- \| --- \| \|  \| \| **Risk analysis excluding patients with outcome prior to the time window** \| \| \| \| \| \| \| \| \| \| \|  \|  \| \| \| Cohort \| \| Patients in cohort \| Patients with outcome \| Risk \| \| \| \| \|  \| \| \|  \| 1 \| DM+AKD+DPP4i with SGLT-2 1 \| 610 \| 49 \| 0.080 \| \| \| \| \|  \| \| \|  \| 2 \| DM+AKD without DPP4i with SGLT-2 1 \| 627 \| 39 \| 0.062 \| \| \| \| \|  \| \| \| \| \| \| \| \| \| \| \| \| \|  \| \| \|  \|  \| \|  \| 95% CI \| z \| p \|  \|  \| \|  \| \| \|  \| **Risk Difference** \| \| 0.018 \| (-0.011, 0.047) \| 1.240 \| 0.215 \|  \|  \| \|  \| \| \|  \| **Risk Ratio** \| \| 1.291 \| (0.861, 1.937) \| N/A \| N/A \|  \|  \| \|  \| \| \|  \| **Odds Ratio** \| \| 1.317 \| (0.851, 2.037) \| N/A \| N/A \|  \|  \| \|  \| \| \| \| \| \| \| \| \| \| \| \| \|  \| \| **Kaplan - Meier survival analysis excluding patients with outcome prior to the time window** \| \| \| \| \| \| \| \| \| \| \|  \| \| \|  \| Cohort \| \| Patients in cohort \| Patients with outcome \| Median survival (days) \| Survival probability at end of time window \| \| \| \|  \| \| \|  \| 1 \| DM+AKD+DPP4i with SGLT-2 1 \| 610 \| 49 \| -- \| 82.32% \| \| \| \|  \| \| \|  \| 2 \| DM+AKD without DPP4i with SGLT-2 1 \| 627 \| 39 \| -- \| 85.70% \| \| \| \|  \| \| \| \| \| \| \| \| \| \| \| \| \|  \| \| \|  \|  \| \| χ^2^ \| df \| p \|  \|  \|  \| \|  \| \| \|  \| **Log-Rank Test** \| \| 0.050 \| 1 \| 0.823 \|  \|  \|  \| \|  \| \| \| \| \| \| \| \| \| \| \| \| \|  \| \| \|  \|  \| \| Hazard Ratio \| 95% CI \| χ^2^ \| df \| p \| \| \|  \| \| \|  \| **Hazard Ratio and Proportionality** \| \| 0.953 \| (0.623, 1.456) \| 3.827 \| 1 \| 0.050 \| \| \|  \| \| \| \| \| \| \| \| \| \| \| \| \|  \| \| **Number of instances excluding patients with outcome prior to the time window** \| \| \| \| \| \| \| \| \| \| \|  \| \| \|  \| Cohort \| \| Patients in cohort \| Patients with outcome \| Mean \| Standard Deviation \| Median \| \| \|  \| \| \|  \| 1 \| DM+AKD+DPP4i with SGLT-2 1 \| 610 \| 49 \| 1.102 \| 0.306 \| 1 \| \| \|  \| \| \|  \| 2 \| DM+AKD without DPP4i with SGLT-2 1 \| 627 \| 39 \| 1.256 \| 0.442 \| 1 \| \| \|  \| \| \| \| \| \| \| \| \| \| \| \| \|  \| \| \|  \|  \| \| t \| df \| p \|  \|  \|  \| \|  \| \| \|  \| **Test Statistics** \| \| -1.932 \| 86 \| 0.057 \|  \|  \|  \| \|  \| \| \| \| \| \| \| \| \| \| \| \| \| **2 MAKE** \| \| \| \| \| \| \| \| \| \| \| \| \|  \| \| **Risk analysis** \| \| \| \| \| \| \| \| \| \| \|  \|  \| \| \| Cohort \| \| Patients in cohort \| Patients with outcome \| Risk \| \| \| \| \|  \| \| \|  \| 1 \| DM+AKD+DPP4i with SGLT-2 1 \| 667 \| 53 \| 0.079 \| \| \| \| \|  \| \| \|  \| 2 \| DM+AKD without DPP4i with SGLT-2 1 \| 667 \| 41 \| 0.061 \| \| \| \| \|  \| \| \| \| \| \| \| \| \| \| \| \| \|  \| \| \|  \|  \| \|  \| 95% CI \| z \| p \|  \|  \| \|  \| \| \|  \| **Risk Difference** \| \| 0.018 \| (-0.009, 0.045) \| 1.284 \| 0.199 \|  \|  \| \|  \| \| \|  \| **Risk Ratio** \| \| 1.293 \| (0.872, 1.916) \| N/A \| N/A \|  \|  \| \|  \| \| \|  \| **Odds Ratio** \| \| 1.318 \| (0.864, 2.011) \| N/A \| N/A \|  \|  \| \|  \| \| \| \| \| \| \| \| \| \| \| \| \|  \| \| **Kaplan - Meier survival analysis** \| \| \| \| \| \| \| \| \| \| \|  \| \| \|  \| Cohort \| \| Patients in cohort \| Patients with outcome \| Median survival (days) \| Survival probability at end of time window \| \| \| \|  \| \| \|  \| 1 \| DM+AKD+DPP4i with SGLT-2 1 \| 667 \| 53 \| -- \| 81.90% \| \| \| \|  \| \| \|  \| 2 \| DM+AKD without DPP4i with SGLT-2 1 \| 667 \| 41 \| -- \| 85.58% \| \| \| \|  \| \| \| \| \| \| \| \| \| \| \| \| \|  \| \| \|  \|  \| \| χ^2^ \| df \| p \|  \|  \|  \| \|  \| \| \|  \| **Log-Rank Test** \| \| 0.145 \| 1 \| 0.703 \|  \|  \|  \| \|  \| \| \| \| \| \| \| \| \| \| \| \| \|  \| \| \|  \|  \| \| Hazard Ratio \| 95% CI \| χ^2^ \| df \| p \| \| \|  \| \| \|  \| **Hazard Ratio and Proportionality** \| \| 0.923 \| (0.611, 1.394) \| 3.986 \| 1 \| 0.046 \| \| \|  \| \| \| \| \| \| \| \| \| \| \| \| \| **3 MACE** \| \| \| \| \| \| \| \| \| \| \| \| \|  \| \| **Risk analysis excluding patients with outcome prior to the time window** \| \| \| \| \| \| \| \| \| \| \|  \|  \| \| \| Cohort \| \| Patients in cohort \| Patients with outcome \| Risk \| \| \| \| \|  \| \| \|  \| 1 \| DM+AKD+DPP4i with SGLT-2 1 \| 437 \| 60 \| 0.137 \| \| \| \| \|  \| \| \|  \| 2 \| DM+AKD without DPP4i with SGLT-2 1 \| 409 \| 44 \| 0.108 \| \| \| \| \|  \| \| \| \| \| \| \| \| \| \| \| \| \|  \| \| \|  \|  \| \|  \| 95% CI \| z \| p \|  \|  \| \|  \| \| \|  \| **Risk Difference** \| \| 0.030 \| (-0.014, 0.074) \| 1.316 \| 0.188 \|  \|  \| \|  \| \| \|  \| **Risk Ratio** \| \| 1.276 \| (0.886, 1.838) \| N/A \| N/A \|  \|  \| \|  \| \| \|  \| **Odds Ratio** \| \| 1.320 \| (0.872, 1.999) \| N/A \| N/A \|  \|  \| \|  \| \| \| \| \| \| \| \| \| \| \| \| \|  \| \| **Kaplan - Meier survival analysis excluding patients with outcome prior to the time window** \| \| \| \| \| \| \| \| \| \| \|  \| \| \|  \| Cohort \| \| Patients in cohort \| Patients with outcome \| Median survival (days) \| Survival probability at end of time window \| \| \| \|  \| \| \|  \| 1 \| DM+AKD+DPP4i with SGLT-2 1 \| 437 \| 60 \| -- \| 70.47% \| \| \| \|  \| \| \|  \| 2 \| DM+AKD without DPP4i with SGLT-2 1 \| 409 \| 44 \| -- \| 71.93% \| \| \| \|  \| \| \| \| \| \| \| \| \| \| \| \| \|  \| \| \|  \|  \| \| χ^2^ \| df \| p \|  \|  \|  \| \|  \| \| \|  \| **Log-Rank Test** \| \| 0.269 \| 1 \| 0.604 \|  \|  \|  \| \|  \| \| \| \| \| \| \| \| \| \| \| \| \|  \| \| \|  \|  \| \| Hazard Ratio \| 95% CI \| χ^2^ \| df \| p \| \| \|  \| \| \|  \| **Hazard Ratio and Proportionality** \| \| 0.901 \| (0.608, 1.335) \| 2.469 \| 1 \| 0.116 \| \| \|  \| \| \| \| \| \| \| \| \| \| \| \| \|  \| \| **Number of instances excluding patients with outcome prior to the time window** \| \| \| \| \| \| \| \| \| \| \|  \| \| \|  \| Cohort \| \| Patients in cohort \| Patients with outcome \| Mean \| Standard Deviation \| Median \| \| \|  \| \| \|  \| 1 \| DM+AKD+DPP4i with SGLT-2 1 \| 437 \| 60 \| 5.283 \| 13.212 \| 1 \| \| \|  \| \| \|  \| 2 \| DM+AKD without DPP4i with SGLT-2 1 \| 409 \| 44 \| 3.023 \| 4.003 \| 1 \| \| \|  \| \| \| \| \| \| \| \| \| \| \| \| \|  \| \| \|  \|  \| \| t \| df \| p \|  \|  \|  \| \|  \| \| \|  \| **Test Statistics** \| \| 1.097 \| 102 \| 0.275 \|  \|  \|  \|   **Without SGLT-2 concurrent usage**   \| **1 Mortality** \| \| \| \| \| \| \| \| \| \| \| \| \| \| \| \| \| --- \| --- \| --- \| --- \| --- \| --- \| --- \| --- \| --- \| --- \| --- \| --- \| --- \| --- \| --- \| --- \| \| **Risk analysis excluding patients with outcome prior to the time window** \| \| \| \| \| \| \| \| \| \| \| \| \| \| \|  \| \| Cohort \| \| \| Patients in cohort \| \| Patients with outcome \| \| Risk \| \| \| \| \| \| \|  \|  \| \| 1 \| DM+AKD+DPP4i without SGLT-2 1 \| \| 5,959 \| \| 946 \| \| 0.159 \| \| \| \| \| \| \|  \|  \| \| 2 \| DM+AKD without DPP4i without SGLT2 1 \| \| 5,879 \| \| 1,031 \| \| 0.175 \| \| \| \| \| \| \|  \| \| \| \| \| \| \| \| \| \| \| \| \| \| \| \| \|  \|  \| \|  \| \| \|  \| \| 95% CI \| \| z \| p \|  \|  \| \| \| \|  \|  \| \| **Risk Difference** \| \| \| -0.017 \| \| (-0.030, -0.003) \| \| -2.424 \| 0.015 \|  \|  \| \| \| \|  \|  \| \| **Risk Ratio** \| \| \| 0.905 \| \| (0.835, 0.981) \| \| N/A \| N/A \|  \|  \| \| \| \|  \|  \| \| **Odds Ratio** \| \| \| 0.887 \| \| (0.806, 0.977) \| \| N/A \| N/A \|  \|  \| \| \| \|  \| \| \| \| \| \| \| \| \| \| \| \| \| \| \| \| \| **Kaplan - Meier survival analysis excluding patients with outcome prior to the time window** \| \| \| \| \| \| \| \| \| \| \| \| \| \| \|  \|  \| \| Cohort \| \| \| Patients in cohort \| \| Patients with outcome \| \| Median survival (days) \| Survival probability at end of time window \| \| \| \| \| \|  \|  \| \| 1 \| DM+AKD+DPP4i without SGLT-2 1 \| \| 5,959 \| \| 946 \| \| -- \| 73.21% \| \| \| \| \| \|  \|  \| \| 2 \| DM+AKD without DPP4i without SGLT2 1 \| \| 5,879 \| \| 1,031 \| \| -- \| 69.21% \| \| \| \| \| \|  \| \| \| \| \| \| \| \| \| \| \| \| \| \| \| \| \|  \|  \| \|  \| \| \| χ^2^ \| \| df \| \| p \|  \|  \|  \| \| \| \|  \|  \| \| **Log-Rank Test** \| \| \| 18.228 \| \| 1 \| \| 0.000 \|  \|  \|  \| \| \| \|  \| \| \| \| \| \| \| \| \| \| \| \| \| \| \| \| \|  \|  \| \|  \| \| \| Hazard Ratio \| \| 95% CI \| \| χ^2^ \| df \| p \| \| \| \| \|  \|  \| \| **Hazard Ratio and Proportionality** \| \| \| 0.825 \| \| (0.756, 0.902) \| \| 2.155 \| 1 \| 0.142 \| \| \| \| \|  \| \| \| \| \| \| \| \| \| \| \| \| \| \| \| \| \| **Number of instances excluding patients with outcome prior to the time window** \| \| \| \| \| \| \| \| \| \| \| \| \| \| \|  \|  \| \| Cohort \| \| \| Patients in cohort \| \| Patients with outcome \| \| Mean \| Standard Deviation \| Median \| \| \| \| \|  \|  \| \| 1 \| DM+AKD+DPP4i without SGLT-2 1 \| \| 5,959 \| \| 946 \| \| 1.217 \| 0.515 \| 1 \| \| \| \| \|  \|  \| \| 2 \| DM+AKD without DPP4i without SGLT2 1 \| \| 5,879 \| \| 1,031 \| \| 1.177 \| 0.449 \| 1 \| \| \| \| \|  \| \| \| \| \| \| \| \| \| \| \| \| \| \| \| \| \|  \|  \| \|  \| \| \| t \| \| df \| \| p \|  \|  \|  \| \| \| \|  \|  \| \| **Test Statistics** \| \| \| 1.852 \| \| 1975 \| \| 0.064 \|  \|  \|  \| \| \| \|  \| \| \| \| \| \| \| \| \| \| \| \| \| \| \| \| \| **2 MAKE** \| \| \| \| \| \| \| \| \| \| \| \| \| \| \| \| \| **Risk analysis** \| \| \| \| \| \| \| \| \| \| \| \| \| \| \|  \| \| Cohort \| \| \| Patients in cohort \| \| Patients with outcome \| \| Risk \| \| \| \| \| \| \|  \|  \| \| 1 \| DM+AKD+DPP4i without SGLT-2 1 \| \| 6,259 \| \| 1,056 \| \| 0.169 \| \| \| \| \| \| \|  \|  \| \| 2 \| DM+AKD without DPP4i without SGLT2 1 \| \| 6,259 \| \| 1,153 \| \| 0.184 \| \| \| \| \| \| \|  \| \| \| \| \| \| \| \| \| \| \| \| \| \| \| \| \|  \|  \| \|  \| \| \|  \| \| 95% CI \| \| z \| p \|  \|  \| \| \| \|  \|  \| \| **Risk Difference** \| \| \| -0.015 \| \| (-0.029, -0.002) \| \| -2.274 \| 0.023 \|  \|  \| \| \| \|  \|  \| \| **Risk Ratio** \| \| \| 0.916 \| \| (0.849, 0.988) \| \| N/A \| N/A \|  \|  \| \| \| \|  \|  \| \| **Odds Ratio** \| \| \| 0.899 \| \| (0.820, 0.985) \| \| N/A \| N/A \|  \|  \| \| \| \|  \| \| \| \| \| \| \| \| \| \| \| \| \| \| \| \| \| **Kaplan - Meier survival analysis** \| \| \| \| \| \| \| \| \| \| \| \| \| \| \|  \|  \| \| Cohort \| \| \| Patients in cohort \| \| Patients with outcome \| \| Median survival (days) \| Survival probability at end of time window \| \| \| \| \| \|  \|  \| \| 1 \| DM+AKD+DPP4i without SGLT-2 1 \| \| 6,259 \| \| 1,056 \| \| -- \| 71.60% \| \| \| \| \| \|  \|  \| \| 2 \| DM+AKD without DPP4i without SGLT2 1 \| \| 6,259 \| \| 1,153 \| \| -- \| 67.38% \| \| \| \| \| \|  \| \| \| \| \| \| \| \| \| \| \| \| \| \| \| \| \|  \|  \| \|  \| \| \| χ^2^ \| \| df \| \| p \|  \|  \|  \| \| \| \|  \|  \| \| **Log-Rank Test** \| \| \| 22.545 \| \| 1 \| \| 0.000 \|  \|  \|  \| \| \| \|  \| \| \| \| \| \| \| \| \| \| \| \| \| \| \| \| \|  \|  \| \|  \| \| \| Hazard Ratio \| \| 95% CI \| \| χ^2^ \| df \| p \| \| \| \| \|  \|  \| \| **Hazard Ratio and Proportionality** \| \| \| 0.817 \| \| (0.752, 0.888) \| \| 4.139 \| 1 \| 0.042 \| \| \| \| \|  \| \| \| \| \| \| \| \| \| \| \| \| \| \| \| \| \| **3 MACE** \| \| \| \| \| \| \| \| \| \| \| \| \| \| \| \| \| **Risk analysis excluding patients with outcome prior to the time window** \| \| \| \| \| \| \| \| \| \| \| \| \| \| \|  \| \| Cohort \| \| \| Patients in cohort \| \| Patients with outcome \| \| Risk \| \| \| \| \| \| \|  \|  \| \| 1 \| DM+AKD+DPP4i without SGLT-2 1 \| \| 4,199 \| \| 882 \| \| 0.210 \| \| \| \| \| \| \|  \|  \| \| 2 \| DM+AKD without DPP4i without SGLT2 1 \| \| 4,339 \| \| 1,006 \| \| 0.232 \| \| \| \| \| \| \|  \| \| \| \| \| \| \| \| \| \| \| \| \| \| \| \| \|  \|  \| \|  \| \| \|  \| \| 95% CI \| \| z \| p \|  \|  \| \| \| \|  \|  \| \| **Risk Difference** \| \| \| -0.022 \| \| (-0.039, -0.004) \| \| -2.427 \| 0.015 \|  \|  \| \| \| \|  \|  \| \| **Risk Ratio** \| \| \| 0.906 \| \| (0.836, 0.981) \| \| N/A \| N/A \|  \|  \| \| \| \|  \|  \| \| **Odds Ratio** \| \| \| 0.881 \| \| (0.795, 0.976) \| \| N/A \| N/A \|  \|  \| \| \| \|  \| \| \| \| \| \| \| \| \| \| \| \| \| \| \| \| \| **Kaplan - Meier survival analysis excluding patients with outcome prior to the time window** \| \| \| \| \| \| \| \| \| \| \| \| \| \| \|  \|  \| \| Cohort \| \| \| Patients in cohort \| \| Patients with outcome \| \| Median survival (days) \| Survival probability at end of time window \| \| \| \| \| \|  \|  \| \| 1 \| DM+AKD+DPP4i without SGLT-2 1 \| \| 4,199 \| \| 882 \| \| -- \| 65.93% \| \| \| \| \| \|  \|  \| \| 2 \| DM+AKD without DPP4i without SGLT2 1 \| \| 4,339 \| \| 1,006 \| \| -- \| 60.70% \| \| \| \| \| \|  \| \| \| \| \| \| \| \| \| \| \| \| \| \| \| \| \|  \|  \| \|  \| \| \| χ^2^ \| \| df \| \| p \|  \|  \|  \| \| \| \|  \|  \| \| **Log-Rank Test** \| \| \| 19.814 \| \| 1 \| \| 0.000 \|  \|  \|  \| \| \| \|  \| \| \| \| \| \| \| \| \| \| \| \| \| \| \| \| \|  \|  \| \|  \| \| \| Hazard Ratio \| \| 95% CI \| \| χ^2^ \| df \| p \| \| \| \| \|  \|  \| \| **Hazard Ratio and Proportionality** \| \| \| 0.815 \| \| (0.744, 0.892) \| \| 1.597 \| 1 \| 0.206 \| \| \| \| \|  \| \| \| \| \| \| \| \| \| \| \| \| \| \| \| \| \| **Number of instances excluding patients with outcome prior to the time window** \| \| \| \| \| \| \| \| \| \| \| \| \| \| \|  \|  \| \| Cohort \| \| \| Patients in cohort \| \| Patients with outcome \| \| Mean \| Standard Deviation \| Median \| \| \| \| \|  \|  \| \| 1 \| DM+AKD+DPP4i without SGLT-2 1 \| \| 4,199 \| \| 882 \| \| 2.927 \| 8.421 \| 1 \| \| \| \| \|  \|  \| \| 2 \| DM+AKD without DPP4i without SGLT2 1 \| \| 4,339 \| \| 1,006 \| \| 2.202 \| 3.657 \| 1 \| \| \| \| \|  \| \| \| \| \| \| \| \| \| \| \| \| \| \| \| \| \|  \|  \| \|  \| \| \| t \| \| df \| \| p \|  \|  \|  \| \| \| \|  \|  \| \| **Test Statistics** \| \| \| 2.480 \| \| 1886 \| \| 0.013 \|  \|  \|  \| \| \|   **Table S7. Logistic regression analysis of covariates associated with major adverse outcomes.**   \|  \| **Univariate** \| \| \| **Muti-variate** \| \| \| \| --- \| --- \| --- \| --- \| --- \| --- \| --- \| \| **All- cause mortality** \|  \|  \|  \|  \|  \|  \| \| Covariates \| Odds ratio \| 95% CI \| p- value \| Adjusted Odds ratio \| 95% CI \| p-value \| \| Age \| 1.034 \| 1.033-1.034 \| < 0.001 \| 1.029 \| 1.028-1.029 \| < 0.001 \| \| Male \| 1.09 \| 1.07-1.10 \| < 0.001 \| 1.25 \| 1.21-1.29 \| < 0.001 \| \| Female \| 0.96 \| 0.95-0.98 \| < 0.001 \| 1.14 \| 1.11-1.18 \| < 0.001 \| \| RAS acting agents \| 1.05 \| 1.03-1.06 \| < 0.001 \| 0.97 \| 0.96-0.99 \| 0.03 \| \| ARB \| 1.03 \| 1.01-1.04 \| 0.002 \| 0.92 \| 0.90-0.94 \| < 0.001 \| \| DPP4i \| 1.02 \| 0.99-1.05 \| 0.12 \| 0.91 \| 0.89-0.94 \| < 0.001 \| \| GLP-1 analogues \| 0.64 \| 0.61-0.67 \| < 0.001 \| 0.69 \| 0.65-0.72 \| < 0.001 \| \| Insulin \| 0.97 \| 0.96-0.99 \| < 0.001 \| 1.08 \| 1.07-1.10 \| < 0.001 \| \| EH \| 1.25 \| 1.23-1.27 \| < 0.001 \| 1.14 \| 1.12-1.16 \| < 0.001 \| \| CAD \| 1.61 \| 1.59-1.63 \| < 0.001 \| 1.26 \| 1.25-1.28 \| < 0.001 \| \| Heart failure \| 1.83 \| 1.81-1.86 \| < 0.001 \| 1.53 \| 1.51-1.55 \| < 0.001 \| \| **MAKE** \|  \|  \|  \|  \|  \|  \| \| Age \| 1.039 \| 1.038-1.040 \| < 0.001 \| 1.031 \| 1.030-1.031 \| < 0.001 \| \| Male \| 1.13 \| 1.11-1.15 \| < 0.001 \| 0.98 \| 0.94-1.03 \| 0.5 \| \| Female \| 0.95 \| 0.93-0.97 \| < 0.001 \| 0.87 \| 0.83-0.91 \| < 0.001 \| \| RAS acting agents \| 1.05 \| 1.02-1.07 \| < 0.001 \| 0.92 \| 0.90-0.94 \| < 0.001 \| \| ARB \| 1.03 \| 1.00-1.06 \| 0.05 \| 0.89 \| 0.86-0.92 \| < 0.001 \| \| DPP4i \| 1.02 \| 0.97-1.07 \| 0.52 \| 0.93 \| 0.89-0.98 \| < 0.01 \| \| GLP-1 analogues \| 0.62 \| 0.56-0.68 \| < 0.001 \| 0.74 \| 0.68-0.80 \| < 0.001 \| \| Insulin \| 1.06 \| 1.03-1.08 \| < 0.001 \| 1.09 \| 1.06-1.12 \| < 0.001 \| \| EH \| 1.20 \| 1.17-1.22 \| < 0.001 \| 1.02 \| 0.99-1.05 \| 0.07 \| \| CAD \| 1.75 \| 1.71-1.78 \| < 0.001 \| 1.30 \| 1.27-1.33 \| < 0.001 \| \| Heart failure \| 2.04 \| 2.00-2.09 \| < 0.001 \| 1.58 \| 1.55-1.62 \| < 0.001 \| \| **MACE** \|  \|  \|  \|  \|  \|  \| \| Age \| 1.033 \| 1.032-1.034 \| < 0.001 \| 1.028 \| 1.027-1.029 \| < 0.001 \| \| Male \| 1.07 \| 1.05-1.09 \| < 0.001 \| 1.18 \| 1.13-1.23 \| < 0.001 \| \| Female \| 0.97 \| 0.95-0.98 \| < 0.001 \| 1.10 \| 1.05-1.15 \| < 0.001 \| \| RAS acting agents \| 1.36 \| 1.33-1.38 \| < 0.001 \| 1.15 \| 1.13-1.18 \| < 0.001 \| \| ARB \| 1.19 \| 1.17-1.22 \| < 0.001 \| 0.91 \| 0.89-0.94 \| < 0.001 \| \| DPP4i \| 1.08 \| 1.04-1.13 \| < 0.001 \| 0.94 \| 0.90-0.98 \| < 0.001 \| \| GLP-1 analogues \| 0.82 \| 0.77-0.87 \| < 0.001 \| 0.86 \| 0.81-0.92 \| < 0.001 \| \| Insulin \| 0.97 \| 0.95-0.99 \| < 0.001 \| 0.89 \| 0.87-0.92 \| < 0.001 \| \| EH \| 1.53 \| 1.50-1.56 \| < 0.001 \| 1.27 \| 1.24-1.30 \| < 0.001 \| \| CAD \| 2.1 \| 2.06-2.14 \| < 0.001 \| 1.50 \| 1.47-1.54 \| < 0.001 \| \| Heart failure \| 1.77 \| 1.73-1.80 \| < 0.001 \| 1.38 \| 1.35-1.41 \| < 0.001 \|   Abbreviations: ARB, angiotensin II receptor blockers; CAD, coronary artery disease; DPP4i, dipeptidyl peptidase-4 inhibitors; EH, essential hypertension; GLP-1 analogues, glucagon like peptide-1-receptor analogues; RAS acting agents, renin-angiotensin system acting agents.  **Table S8. Heart failure**   \| **5 Heart failure** \| \| \| \| \| \| \| \| \| \| \| \| \| \| \| \| \| --- \| --- \| --- \| --- \| --- \| --- \| --- \| --- \| --- \| --- \| --- \| --- \| --- \| --- \| --- \| --- \| \| **Risk analysis excluding patients with outcome prior to the time window** \| \| \| \| \| \| \| \| \| \| \| \| \| \| \|  \| \| Cohort \| \| \| Patients in cohort \| \| Patients with outcome \| \| Risk \| \| \| \| \| \| \|  \|  \| \| 1 \| DM+AKD+DPP4i 1 \| \| 4,736 \| \| 594 \| \| 0.125 \| \| \| \| \| \| \|  \|  \| \| 2 \| DM+AKD without DPP4i 1 \| \| 4,848 \| \| 532 \| \| 0.110 \| \| \| \| \| \| \|  \| \| \| \| \| \| \| \| \| \| \| \| \| \| \| \| \|  \|  \| \|  \| \| \|  \| \| 95% CI \| \| z \| p \|  \|  \| \| \| \|  \|  \| \| **Risk Difference** \| \| \| 0.016 \| \| (0.003, 0.029) \| \| 2.384 \| 0.017 \|  \|  \| \| \| \|  \|  \| \| **Risk Ratio** \| \| \| 1.143 \| \| (1.024, 1.276) \| \| N/A \| N/A \|  \|  \| \| \| \|  \|  \| \| **Odds Ratio** \| \| \| 1.163 \| \| (1.027, 1.318) \| \| N/A \| N/A \|  \|  \| \| \| \|  \| \| \| \| \| \| \| \| \| \| \| \| \| \| \| \| \| **Kaplan - Meier survival analysis excluding patients with outcome prior to the time window** \| \| \| \| \| \| \| \| \| \| \| \| \| \| \|  \|  \| \| Cohort \| \| \| Patients in cohort \| \| Patients with outcome \| \| Median survival (days) \| Survival probability at end of time window \| \| \| \| \| \|  \|  \| \| 1 \| DM+AKD+DPP4i 1 \| \| 4,736 \| \| 594 \| \| -- \| 77.40% \| \| \| \| \| \|  \|  \| \| 2 \| DM+AKD without DPP4i 1 \| \| 4,848 \| \| 532 \| \| -- \| 78.12% \| \| \| \| \| \|  \| \| \| \| \| \| \| \| \| \| \| \| \| \| \| \| \|  \|  \| \|  \| \| \| χ^2^ \| \| df \| \| p \|  \|  \|  \| \| \| \|  \|  \| \| **Log-Rank Test** \| \| \| 0.484 \| \| 1 \| \| 0.487 \|  \|  \|  \| \| \| \|  \| \| \| \| \| \| \| \| \| \| \| \| \| \| \| \| \|  \|  \| \|  \| \| \| Hazard Ratio \| \| 95% CI \| \| χ^2^ \| df \| p \| \| \| \| \|  \|  \| \| **Hazard Ratio and Proportionality** \| \| \| 1.042 \| \| (0.927, 1.172) \| \| 0.063 \| 1 \| 0.801 \| \| \| \| \|  \| \| \| \| \| \| \| \| \| \| \| \| \| \| \| \| \| **Number of instances excluding patients with outcome prior to the time window** \| \| \| \| \| \| \| \| \| \| \| \| \| \| \|  \|  \| \| Cohort \| \| \| Patients in cohort \| \| Patients with outcome \| \| Mean \| Standard Deviation \| Median \| \| \| \| \|  \|  \| \| 1 \| DM+AKD+DPP4i 1 \| \| 4,736 \| \| 594 \| \| 7.094 \| 11.473 \| 3 \| \| \| \| \|  \|  \| \| 2 \| DM+AKD without DPP4i 1 \| \| 4,848 \| \| 532 \| \| 5.526 \| 8.523 \| 2 \| \| \| \| \|  \| \| \| \| \| \| \| \| \| \| \| \| \| \| \| \| \|  \|  \| \|  \| \| \| t \| \| df \| \| p \|  \|  \|  \| \| \| \|  \|  \| \| **Test Statistics** \| \| \| 2.579 \| \| 1124 \| \| 0.010 \|  \|  \|  \| \| \| \|  \| \| \| \| \| \| \| \| \| \| \| \| \| \| \| \| | | | | | | |  |  |

1. **Supplementary figures**

**Figure S1. Cohort Construction Flowchart.** The flowchart illustrated the construction of the cohort using the TriNetX platform.


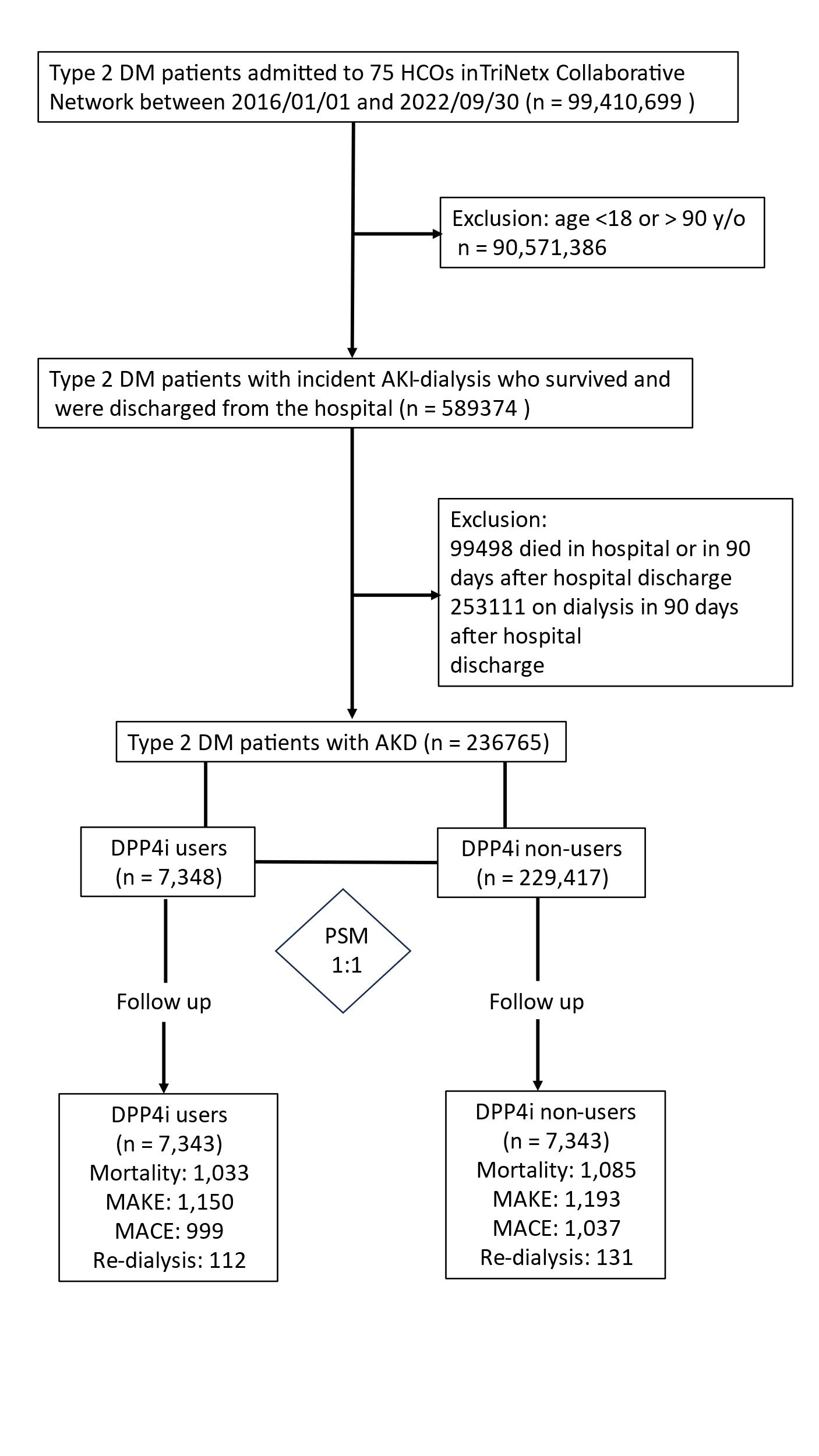


**Figure S2. The inclusion criteria for the study.**


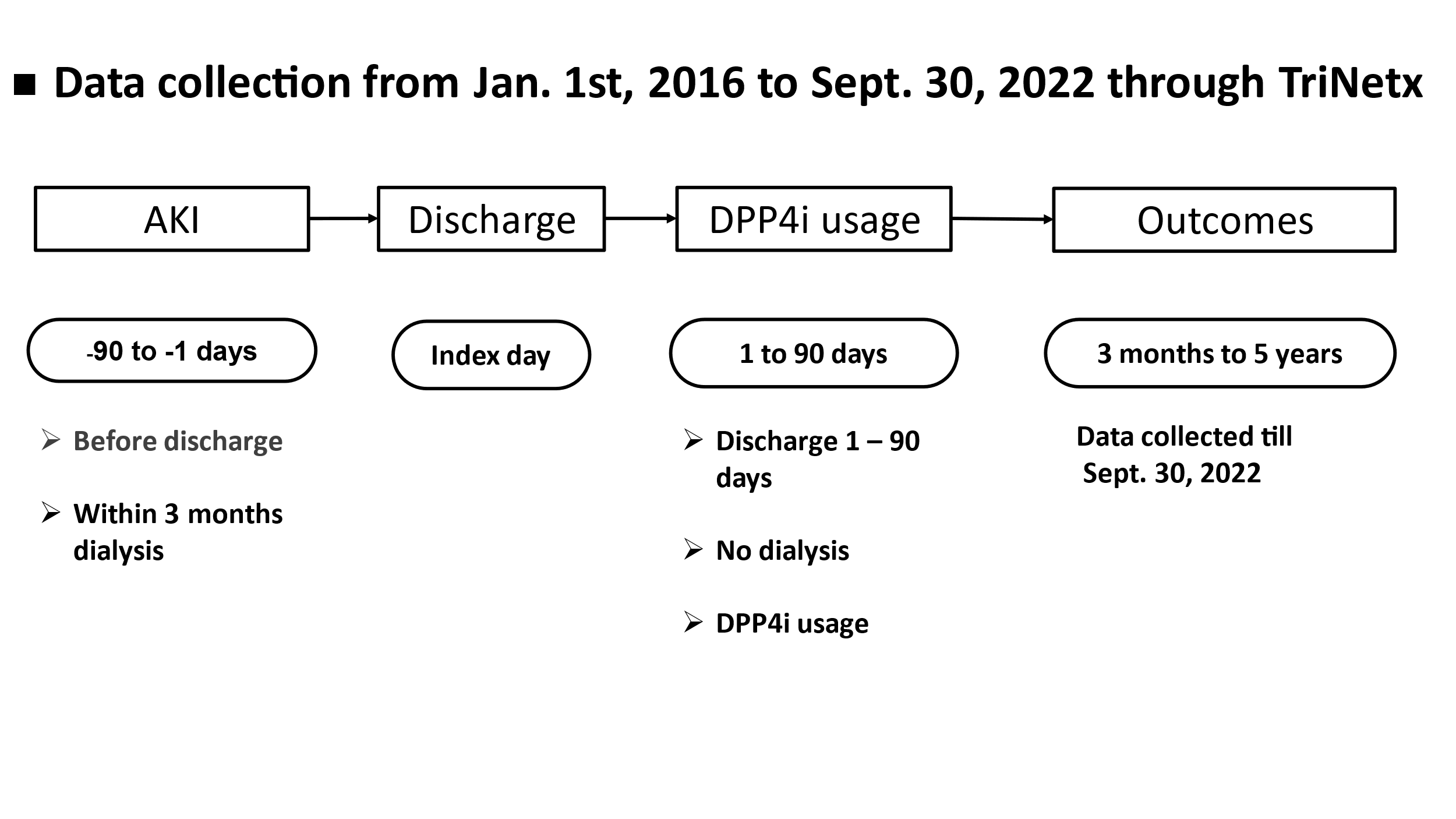


**Figure S3. Pre-specified outcomes of DPP4i users compared to the control group in the external validation.** The forest plot illustrated the hazard ratio of prespecified outcomes in the Chang Gang Research Database. The DPP4i users in DM patients had favorable outcomes compared to DPP4i non-users in the all-cause mortality, MAKE, MACE, and re-dialysis.

**Abbreviations:** AKI, acute kidney injury; CI, confidence interval; DM, diabetes mellitus; DPP4i, dipeptidyl peptidase 4 inhibitor; HR, hazard ratio; MACE, major adverse cardiovascular events; MAKE, major adverse kidney events.


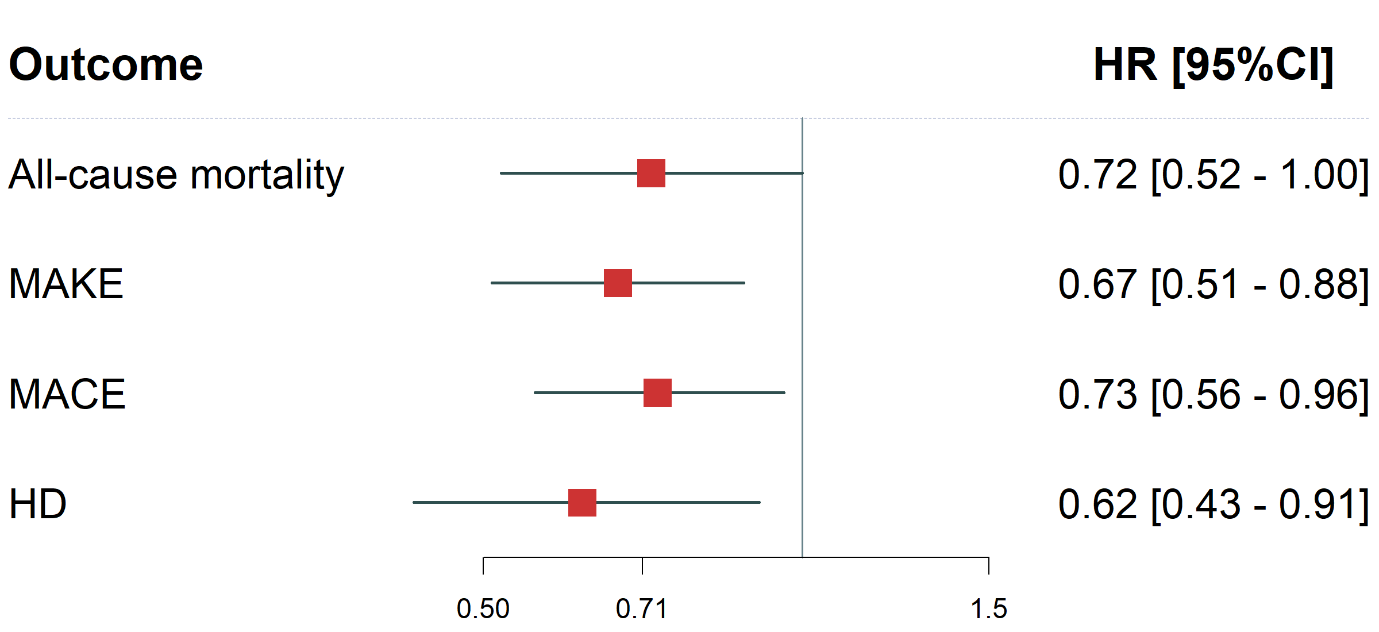


**Figure S4. Kaplan-Meier curves of the pre-specified long-term outcomes by external validation.** Kaplan-Meier curves for the pre-specified 5-year outcomes of DPP4i users and the control group identified in the Chang Gung Research Database[7] were illustrated as follows: (A) all-cause mortality (log-rank P = 0.047), (B) MAKE (log-rank P = 0.004), (C) MACE (log-rank P = 0.022), (D) re-dialysis (log-rank P = 0.013). The yellow line corresponded to DPP4i users, while the blue line represented DPP4i non-users.


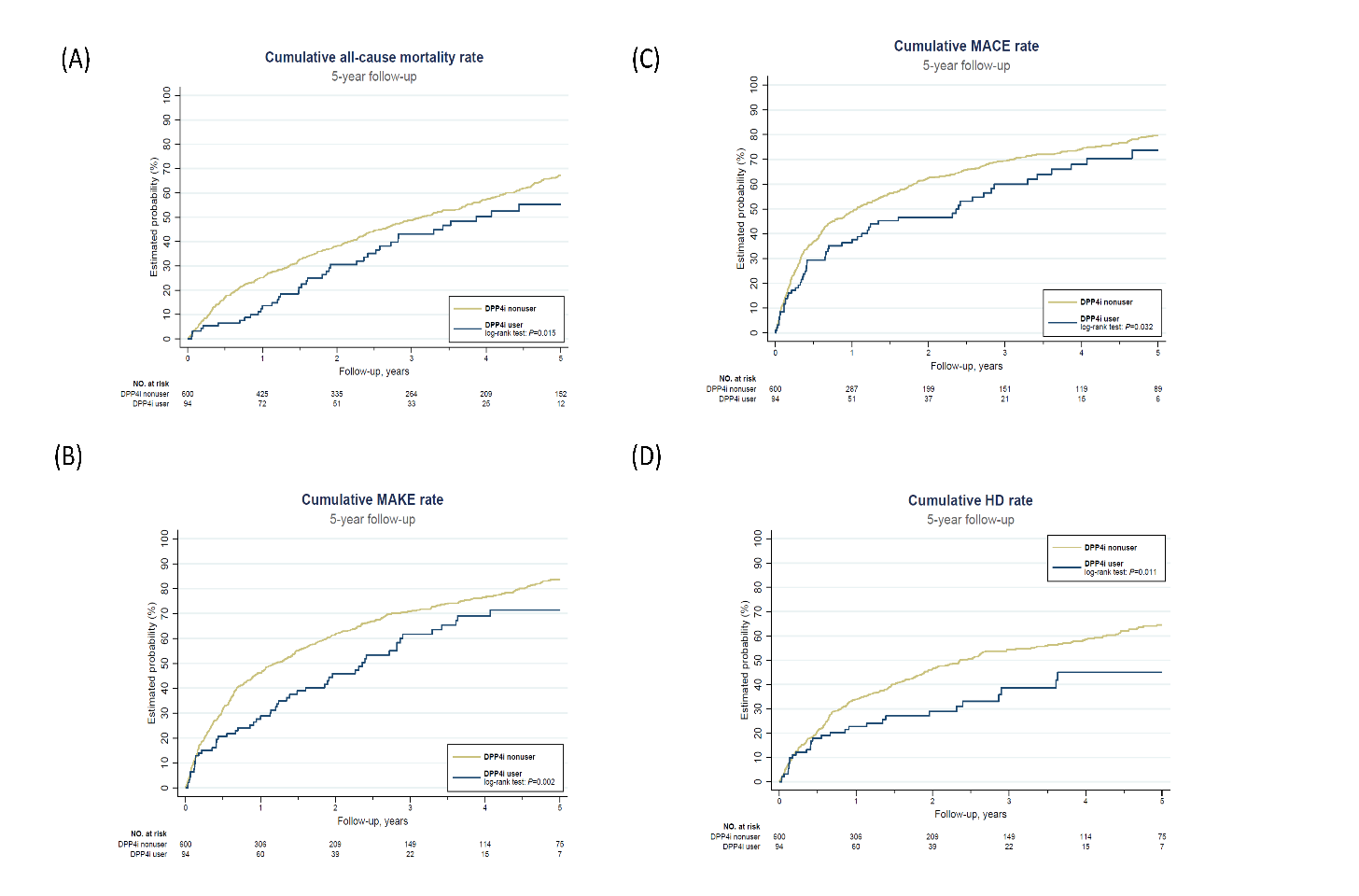


**Abbreviations:** DPP4i, dipeptidyl peptidase 4 inhibitor; HD, re-dialysis; MACE, major adverse cardiovascular event; MAKE, major adverse kidney event.

**Supplemental reference section**

1. Topaloglu U, Palchuk MB: **Using a Federated Network of Real-World Data to Optimize Clinical Trials Operations**. *JCO Clin Cancer Inform* 2018, **2**:1-10.

2. Palchuk MB, London JW, Perez-Rey D, Drebert ZJ, Winer-Jones JP, Thompson CN, Esposito J, Claerhout B: **A global federated real-world data and analytics platform for research**. *JAMIA Open* 2023, **6**(2):ooad035.

3. MacKenzie SL, Wyatt MC, Schuff R, Tenenbaum JD, Anderson N: **Practices and perspectives on building integrated data repositories: results from a 2010 CTSA survey**. *J Am Med Inform Assoc* 2012, **19**(e1):e119-124.

4. Hudson CL, Topaloglu U, Bian J, Hogan W, Kieber-Emmons T: **Automated Tools for Clinical Research Data Quality Control using NCI Common Data Elements**. *AMIA Jt Summits Transl Sci Proc* 2014, **2014**:60-69.

5. Kahn MG, Brown JS, Chun AT, Davidson BN, Meeker D, Ryan PB, Schilling LM, Weiskopf NG, Williams AE, Zozus MN: **Transparent reporting of data quality in distributed data networks**. *EGEMS (Wash DC)* 2015, **3**(1):1052.

6. Weiskopf NG, Hripcsak G, Swaminathan S, Weng C: **Defining and measuring completeness of electronic health records for secondary use**. *J Biomed Inform* 2013, **46**(5):830-836.

7. Shao SC, Chan YY, Kao Yang YH, Lin SJ, Hung MJ, Chien RN, Lai CC, Lai EC: **The Chang Gung Research Database-A multi-institutional electronic medical records database for real-world epidemiological studies in Taiwan**. *Pharmacoepidemiol Drug Saf* 2019, **28**(5):593-600.
